# Metabolomics of dates (*Phoenix dactylifera*) reveals a highly dynamic ripening process accounting for major variation in fruit composition

**DOI:** 10.1186/s12870-015-0672-5

**Published:** 2015-12-16

**Authors:** Ilhame Diboun, Sweety Mathew, Maryam Al-Rayyashi, Mohamed Elrayess, Maria Torres, Anna Halama, Michaël Méret, Robert P. Mohney, Edward D. Karoly, Joel Malek, Karsten Suhre

**Affiliations:** Department of Physiology and Biophysics, Weill Cornell Medical College, Qatar Foundation – Education City, PO Box 24144, Doha, Qatar; Genomics Laboratory, Weill Cornell Medical College, Doha, Qatar; Life sciences research division, ADLQ Doha, Qatar; Department of Genetic Medicine, Weill Cornell Medical College, Doha, Qatar; MetaSysX GmbH, Potsdam, Germany; Metabolon, Inc., Durham, USA

**Keywords:** Date fruit, Ripening, Metabolomics, Date palm, Soft dates varieties, Dry dates varieties, SIMCA, OPLS, PCA, Multivariate

## Abstract

**Background:**

Dates are tropical fruits with appreciable nutritional value. Previous attempts at global metabolic characterization of the date metabolome were constrained by small sample size and limited geographical sampling. In this study, two independent large cohorts of mature dates exhibiting substantial diversity in origin, varieties and fruit processing conditions were measured by metabolomics techniques in order to identify major determinants of the fruit metabolome.

**Results:**

Multivariate analysis revealed a first principal component (PC1) significantly associated with the dates’ countries of production. The availability of a smaller dataset featuring immature dates from different development stages served to build a model of the ripening process in dates, which helped reveal a strong ripening signature in PC1. Analysis revealed enrichment in the dry type of dates amongst fruits with early ripening profiles at one end of PC1 as oppose to an overrepresentation of the soft type of dates with late ripening profiles at the other end of PC1. Dry dates are typical to the North African region whilst soft dates are more popular in the Gulf region, which partly explains the observed association between PC1 and geography. Analysis of the loading values, expressing metabolite correlation levels with PC1, revealed enrichment patterns of a comprehensive range of metabolite classes along PC1. Three distinct metabolic phases corresponding to known stages of date ripening were observed: An early phase enriched in regulatory hormones, amines and polyamines, energy production, tannins, sucrose and anti-oxidant activity, a second phase with on-going phenylpropanoid secondary metabolism, gene expression and phospholipid metabolism and a late phase with marked sugar dehydration activity and degradation reactions leading to increased volatile synthesis.

**Conclusions:**

These data indicate the importance of date ripening as a main driver of variation in the date metabolome responsible for their diverse nutritional and economical values. The biochemistry of the ripening process in dates is consistent with other fruits but natural dryness may prevent degenerative senescence in dates following ripening. Based on the finding that mature dates present varying extents of ripening, our survey of the date metabolome essentially revealed snapshots of interchanging metabolic states during ripening empowering an in-depth characterization of underlying biology.

**Electronic supplementary material:**

The online version of this article (doi:10.1186/s12870-015-0672-5) contains supplementary material, which is available to authorized users.

## Background

Date fruits from the date palm tree (*Phoenix dactylifera*) constitute an iconic and economical asset in the Arab world. Date palm cultivation plays an important role in sustaining the ecological system in the region and is also practiced in many other areas in the world notably Southern Eastern Asia, Southern Europe, Latin America and the USA. Unlike palm trees that can tolerate various types of climates, the quality of the fruit is dependent on the climatic and agricultural conditions [1]. Date composition varies amongst different varieties [2] and within the same variety owing to pre and post-harvest conditions [3]. The ripening and maturation process, in particular, accounts for major variation in date composition [4]. The development of the date fruit occurs in four stages known by their Arabic names as *Kimri, Khalal*, *Rutab* and *Tamr* [1, 5]. In the Kimri stage, the date fruit has a hard green texture and shows a rapid gain in size and moisture as well as elevated levels of acidic substances and astringent tannins [4]. Dates show the highest protein and free amino acid content at the Kimri green stage, which continues to decrease throughout the ripening process [6, 7]. A change in color from green to yellow (or pink in some varieties) caused by the degradation of chlorophyll, marks the transition to the Khalal stage that corresponds to the breaker stage in other fruits including tomato and strawberry [8]. The Khalal stage is also characterized by a steady loss of moisture and a sudden rise in the level of non-reducing sugars, mainly sucrose [9]. Softening of the fruit begins at this stage and reaches its optimum level at the advanced Rutab stage. The latter is characterized by increased aroma [10] and fruit browning [4]. Rutab dates are sold as fresh fruits and are perishable. Only after further loss of moisture to less than 25 % and concurrent buildup of reducing sugars at the Tamr stage does the fruit become dry and storable [11]. The drying process can cause a reduction in the level of certain metabolites such as anthocyanins [11] and vitamin C [1] whilst promoting others including reducing sugars [1], unsaturated fatty acids [12] and Maillard substances [13].

Three main types of date fruits are known as soft, semi-dry and dry. Soft dates present a moisture level as high as 30 % at the end of the ripening process. They are highly susceptible to pathogens and often fail to dry on the trees. Sun drying of soft dates at the Rutab stage is common; however, the delicacy of the fruit at this stage with some cultivars may result in harvesting early Khalal followed by artificial ripening [4]. Importantly, soft dates maintain their soft texture after artificial drying. The semi-dry varieties of dates, of which Deglet Noor is most famous, are more firm, present less moisture and tend to dry naturally [1]. The dry varieties present even firmer texture, are most dry amongst all types featuring less than 20 % moisture content and can be discolored [1]. The dry and semi-dry varieties are sometimes rehydrated following harvest to meet quality standards [4]. At the biochemical level, the semi-dry and dry varieties are characterized by a higher ratio of sucrose to reducing sugars unlike the soft types which contain mostly reducing sugars [14]. Differences between the soft, semi dry and dry types of dates extend beyond composition, phenotype and post-harvest treatment to climatic requirements. Dry dates require hot dry environment for optimal growth and maturation whereas soft dates can tolerate some humidity and necessitate less heat units [15, 16]. Genetic analysis of Tunisian cultivars representative of the soft and dry types revealed a significant between-population genetic separation and a significant association between type and genetic markers [17, 18]. Importantly, date palms producing soft date varieties show different tree phenotypes to those producing dry varieties [18, 19].

Metabolomics techniques have offered a promising approach for bridging the gap between genotype and phenotype [20] and have been successfully deployed to study various aspects of fruit and seed biology [13, 21, 22]. Previous metabolomics measurements of dates were limited by a small number of date varieties and confined geographical sampling [10, 12, 13]. In total, eight varieties of dates, all local to Southern Tunisia, featuring three different development stages were measured by HPLC and GC-MC techniques by El Arem and colleagues [10, 12]. The measured volatile and non-volatile metabolites were found to significantly vary between development stages and cultivars. More recently, Farag et al. used sugars and flavonols to classify twenty one Egyptian date varieties into distinct clusters, using a combined UPLC/GC-MS approach [13]. In this study, a comprehensive UPLC-MS and GC-MS metabolomics measurement of two large cohorts of mature date fruits exhibiting substantial variation in origin, variety and post-harvest treatment was performed. The aim was to assess the factor(s) likely to contribute to variation in the date metabolome; in particular the development effect, which was modelled from a separate dataset of immature dates. We predict that our findings are applicable to the larger date population given the sample size and heterogeneity of fruit conditions.

## Methods

### Collection and phenotypic characterization of date fruits

#### Mature fruits

**Fig. 1:**
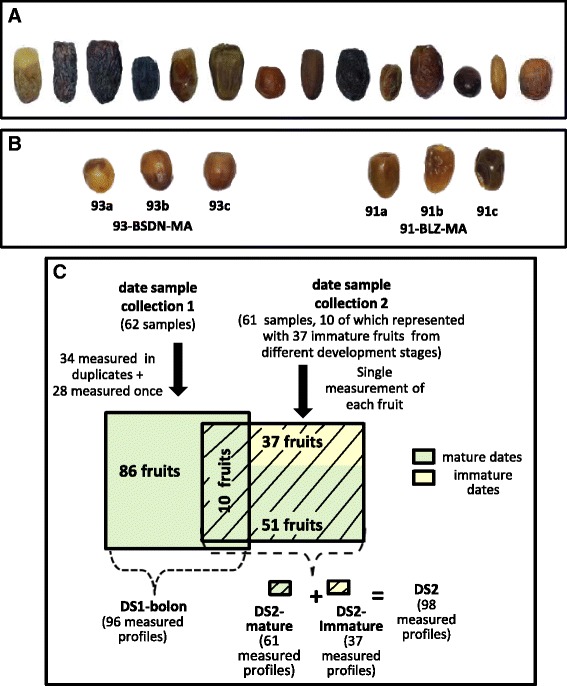
Images of dates. **a** A subset of 14 mature dates representing the 14 countries sampled in this study and reflecting diversity in phenotype. **b** Immature dates from two date samples 93-BSDN-MA and 91-BLZ-MA from the second sample collection. Each fruit is labeled with an ID featuring a letter that indicates its rank by extent of ripening relative to the remaining fruits within the sample (refer to methods). **c** Summary of the date metabolomics datasets measured by Metabolon: 10 fruits from the first sample collection were measured again with fruits from the second sample collection to account for batch measurement effect. All fruits from the first collection were considered mature (shown in green) whilst some fruits from the second sample collection displayed a phenotype indicative of ongoing ripening (refer to methods) and were therefore considered immature (shown in yellow). DS1 has the suffix ‘-bolon’ attached to distinguish it from the MetaSysX measurement of the same fruits from the first sample collection. The second sample collection was only measured by Metabolon

In the present study, 109 unique date varieties (*Phoenix dactylifera*) from 14 countries were collected in two separate occasions: A first collection took part in 2012 and a second one in 2013. The term *variety* is used here to describe a distinct phenotypic class of dates and if the same variety was collected from different countries, a different sample ID was assigned to each collected sample per country. Photos of fruits from 14 date samples collected each from a different country can be found in Fig. 1a. With each date sample, a handful of fruits were selected for pre-processing. Each fruit was weighed and the average weight was recorded for each date sample. Two fruits were halved to get a longitudinal and cross sectional view of the pericarp and seed. An international ColorChecker Color-Rendition Chart (ColorChecker Classic, X-Rite, USA) and a 20 cm ruler were positioned along the fruits on a white background under artificial light and a photograph was taken using a Canon Power Shot S100 USA camera loaded on a pre-set tripod. An example photo can be found in Additional file 1: Figure S1. RGB color values were extracted from all fruits showing on a given photo using Matlab libraries and the results were averaged for each color range separately. Readings from color charts from all processed photos were used to calibrate color measurement across the photos. Further phenotype characterization of the date samples consisted of classification into soft, semi-dry and dry types by reference to the literature as well as moisture content measurement of one representative fruit per date sample. Moisture measurement was performed for a random third of the date samples and was based on calculating the percentage of fruit weight-loss following a 116-h incubation in a 105 °C oven. A full listing of all varieties included in this study together with information on their country of production, collection point and type can be found in Additional file 2. Summary statistics for each sample collection including the count of varieties, samples and the frequency of samples per country of production are shown in Table 1-A. Overall, dates from the first sample collection were mostly from the Gulf region obtained in a fairly dried condition from shops and festivals whilst the second sample collection was dominated by North African dates obtained mostly fresh from the palm trees. For the second collection of dates, field work permissions were obtained verbally from owners of visited oases. The marketed versus fresh nature of dates between the two sample collections implies varying post-harvest conditions. All collected dates with homogenous brown color were further dried by exposing them to open air for two weeks before further processing. In general, dates were considered mature if the low moisture prevented any further change in their appearance. Notably, maturity is attained naturally with the dry class of dates but often artificially with the soft class of dates owing to intrinsically higher moisture levels (refer to background for further details).

**Table 1:**
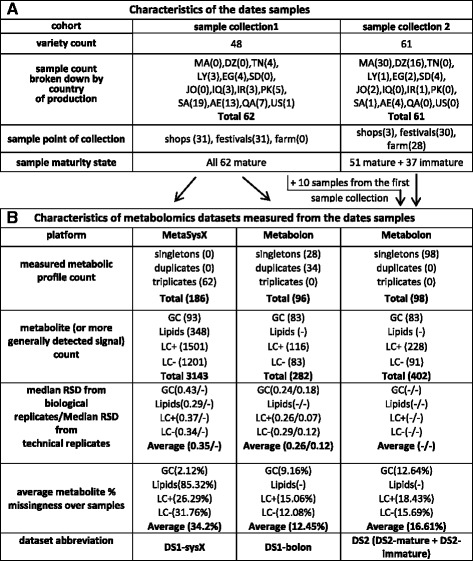
Summary statistics from collected dates and their measured metabolomics data

A) Overview of the date cohorts from the first and second sample collection. Countries are denoted by their international ISO Alpha-2 code as follows: MA (Morocco), DZ (Algeria), TN (Tunisia), LY (Libya), EG (Egypt), SD (Sudan), JO (Jordan), SA (Saudi Arabia), IQ (Iraq), IR (Iran), AE (United Arab Emirates), QA (Qatar), PK (Pakistan), US (United States). Dates from the first sample collection contained largely mature dates. In contrast, 37 immature date fruits corresponding to 10 varieties were included in the second sample collection. B) Summary statistics of the metabolomics data measured from the first and second sample collection. To account for batch effect, 10 samples from the first sample collection were measured again along dates from the second sample collection. Dates from the second sample collection were only measured by Metabolon unlike dates from the first sample collection which were measured by MetaSysX and Metabolon. The median RSD (RSD = sdandard deviation/mean) from biological replicates is a combination of technical and biological variation whilst that from technical replicates only expresses technical variation

#### Immature fruits

With the second sample collection, while harvesting ripened fruits from the palm trees, immature fruits still undergoing ripening activity and occasionally late green Kimri fruits from the pre-ripening stage were collected when available. In total, 37 immature date fruits, corresponding to 10 date samples, were collected. With each of the 10 samples, the immature fruits were ranked by their extent of ripening based on visual assessment of color change and skin wrinkling. Each fruit was given an ID based on a combination of the sample number and a letter reflecting the fruit rank within the sample. A full listing of all immature fruit IDs and corresponding sample IDs is given in Table 2. Photos of immature fruits from two date samples are shown in Fig. 1b.

**Table 2:** Listing of immature date fruits from the second sample collection

Date sample number	Date sample ID	Immature fruit ID
85	85-AZGHZ-MA	85A,85B,85C
87	87-TZGRT-MA	87A,87B,87C
89	89-KLMR-MA	89A,89B,89C,89D,89E
90	90-MJL-MA	90A,90B,90C,90D
91	91-BLZT-MA	91A,91B,91C
92	92-SHTW-MA	92A,92B,92C,92D,92E,92 F
93	93-BSDN-MA	93A,93B,93C
97	97-THMT-MA	97A,97B,97C
99	99-MJN-MA	99A,99B,99C,99D
103	103- TZW-MA	103A,103B,103C

Overall, 37 immature fruits were collected from 10 date samples. Each fruit was assigned an ID based on a combination of the date sample number and a letter expressing the fruit’s extent of ripening, as judged by eye, relative to the remaining fruits within the sample. It is important to note that these letters are only meaningful within a sample and are not comparable between samples

### Metabolite measurement of the date samples

#### Dates preprocessing and measurement protocols

The metabolic content of the date fruits from the second sample collection was measured separately a year after samples from the first collection were measured. The first collection of dates was preprocessed by MetaSysX GmbH. and measured by both MetaSysX GmbH. and Metabolon Inc., USA. Dates from the second collection were preprocessed and measured by Metabolon Inc., USA alone. The protocols for sample processing and metabolomics measurement by both MetaSysX and Metabolon are described in details in Additional file 3.

Briefly, with MetaSysX, 50 mg of the peel and flesh of the date fruits were flash frozen in liquid nitrogen and extracted according to standardized procedures [23]. The dried metabolite extracts were measured with a *Waters* ACQUITY Reversed Phase Ultra Performance Liquid Chromatography (RP-UPLC) coupled to a *Thermo-Fisher Exactive* mass spectrometer which consists of an ElectroSpray Ionization source (ESI) and an Orbitrap mass analyzer. C8 and C18 columns were used for the lipophilic and the hydrophilic measurements, respectively. Chromatograms were recorded in Full Scan MS mode (Mass Range [100–1500]) [23]. Chromatograms from the UPLC-FT-MS runs were analyzed and processed using the software REFINER MS® 7.5 (Genedata, Switzerland). The data were further filtered and analyzed using in-house software tools (refer to Additional file 3). The samples were also measured using the *Agilent Technologies* GC coupled to a *Leco Pegasus HT* mass spectrometer which consists of an EI ionization source and a TOF mass analyzer. Column: 30 meters DB35; Starting temp: 85 °C for 2 min; Gradient: 15 °C per min up to 360 °C. NetCDF files exported from the Leco Pegasus software were imported into “*R*”. The Bioconductor package *TargetSearch* was used to transform retention time to retention index (RI), to align the chromatograms, to extract the peaks and to annotate them by comparing the spectra and the RI to the GMD [24, 25]. Obtained data from both platforms were normalized according to sample weight and to the measurement day to minimize process error over the course of many days of measurement.

With Metabolon, date samples were prepared and extracted according to the standard solvent extraction method by Metabolon Inc. [26]. The UPLC/MS/MS analysis was based on the Waters ACUITY ultra performance liquid chromatography (Waters Corporation, USA) and the ThermoFischer Scientific Orbitrap Elite high-resolution accurate mass spectrometer (Thermo Fischer Scientific Inc., USA) equipped with a heated electrospray ionization (HESI) source and an Orbitrap mass analyzer. The dried sample extracts for the LC positive and LC negative mode were reconstituted in acidic and basic LC- compatible solvents. Two independent injections were performed on each sample using separate dedicated columns. The mass spectra analysis alternated between MS and data dependent MS^2^ scans using dynamic exclusion. With GC/MS, the samples were further dried under vacuum desiccation for an entire day and derivatized under dried nitrogen using bistrimethyl-silyl-trifluoroacetamide (BSTFA). The GS/MS analysis was based on a Thermo Finnigan™ TRACE™ DSQ™ (ThermoFinnigan, USA) fast-scanning single –quadrupole mass spectrophotometer using electron impact ionization source. The GC column was 5 % phenyl and the temperature ramp range was from 40 to 300 °C in a time span of 16 min. The raw data files from both platforms were extracted using the in-house informatics system (refer to Additional file 3). A reference library maintained by Metabolon Inc. [27], consisting of chemical standards with retention time, retention index, mass to charge ratio (m/z) and chromatographic data including MS/MS spectral data was used to identify metabolites in experimental samples as detailed in [28]. In this study, the samples were analyzed over a span of two or three days, and therefore data normalization step was performed to correct variation from instrument inter-day tuning differences.

#### Measurement experimental design

With the first collection of dates containing 62 date samples, the MetaSysX measurement was done in triplicates yielding a total of 186 measured metabolic profiles (Table 1-B). With Metabolon, 34 samples were measured in duplicates whilst the 28 remaining as singletons, amounting to 96 measured metabolic profiles (Table 1-B, Fig. 1c). For the rest of this article, we will refer to the latter as ‘DS1-bolon’ whilst the former metabolomics dataset will be referred to as ‘DS1-sysX’. Dates from the second sample collection were measured by Metabolon only and therefore the derived metabolomics data will be referred to in short as ‘DS2’. DS1-bolon and DS2 metabolomics data can be found in Additional file 4 & Additional file 5 respectively. The experimental design consisted of a singleton measurement of each of the 51 mature date samples (Table 1-B, Fig. 1c) and similarly the 37 immature fruits were each measured once. To account for batch measurement effect, 10 fruits from the first sample collection were measured again along the 88 fruits from the second collection, resulting in 98 measured metabolic profiles (Table 1-B). We distinguish between metabolomics data from the 37 immature and 61 mature date samples (inclusive of the 10 samples from the first collection) using the terms ‘DS2-immature’ and ‘DS2-mature’ respectively (Fig. 1c). The sample characteristics of DS1-sysX, DS1-bolon and DS2 as discussed here are summarized in Table 1-B. Since Metabolon measured datasets were extensively used in this paper, they are further illustrated in Fig. 1c.

### Statistical analysis of metabolomics data

#### Data preprocessing and platform comparison

Metabolomics data, were log-transformed and scaled so that the median measurement value from each measured metabolic profile was equal to the overall median from the whole dataset. This normalization was done separately for DS1-sysX, DS1-bolon and DS2. By default, biological replicates (when available) were not combined and measurement from each replicate was treated as a separate metabolic profile. However, with few analyses, a single measurement from each date sample was required and the replicates were averaged. This will be clearly indicated where applicable. Comparison of platforms was based on average metabolite missingness level across samples and the median relative standard deviation (RSD) across biological replicates. RSD was expressed as metabolite-wise standard deviation from replicates divided by the mean. With Metabolon measurement of samples from the first collection (or DS1-bolon), data from technical replicates were available from repeated measurement of a homogenous mixture of pooled samples (refer to Additional file 3). The median RSD from these technical replicates was calculated for assessment of data quality by Metabolon.

#### Non-supervised PCA analysis of mature dates and quality control

The multivariate statistical analysis package SIMCA v13.0.3 was used to perform PCA on DS1-bolon, DS1-sysX and DS2-mature separately to characterize collective metabolic variation underlying significant proportions of the variance from the respective datasets. Simca default metabolite missingness threshold of 50 % was used [29]. The significance of the extracted principal components was derived from SIMCA via built-in cross validation where for each component consecutively, parts of the data are alternatingly kept out of the model then predicted [29]. Based on the PC1/PC2 two dimensional space, date samples 78-BZGZ-MA and 105-ZGHL-EG from DS2-mature located outside the Hotelling’s 95 % confidence ellipse interval were considered outliers and excluded from further analysis of the dataset [29].

#### SIMCA OPLS-DA and O2PLS-DA models of the dates ripening process

Metabolic signature of date ripening was modeled from analysis of the development stage dataset, or DS2-immature, a subset of the second date sample collection as follows: Initially, PCA analysis was run on measured metabolomics data to confirm the within-sample ranking of individual fruits previously set by visual assessment of the fruits’ extent of ripening (refer to the previous section). The PCA analysis revealed clusters of fruits with comparable ripening profiles across samples (more details in the results section). These clusters were used to define development stage classes that served as a training set for an OPLS-DA classifier [29, 30]. Applying the classifier on the rest of the samples in DS2 led to the calculation of class prediction scores indicative of the samples’ ripening metabolic states. For DS1-bolon, the OPLS-DA model trained on DS2-immature data was not suitable owing to likely differences between batch measurements. Also, unlike the second collection of dates, no development stage dataset was included in the first collection. Instead, we developed a strategy based on the 10 fruits from the first sample collection which were measured again along the samples from the second collection. Because the samples in question were included in both batch measurements, they will be referred to as *batch 1&2 samples* for the remaining parts of this article. Our strategy for predicting the ripening states of dates from the first sample collection is here described: First, we used the OPLS-DA model previously trained on the DS2-immature samples to predict the development classes of *batch 1&2 samples* based on their DS2 data from the same batch measurement as the training set. This class information was used to train an O2PLS-DA classifier on the same samples (batch 1&2 samples) based on their batch 1 and 2 metabolomics measured data. The O2PLS-DA procedure [29, 30] is able to identify metabolites consistently differentiating between the different classes in the training set based on multiple measurements of the training set (here from different batch reading). The integrative nature of the O2PLS-DA model meant that it could be used to calculate class prediction scores for dates from the first and second sample collection. The scores from the first sample collection served to indicate the ripening states of these date samples whilst the scores from the second collection served to optimize and validate the O2PLS-DA model by drawing a comparison to the class prediction scores for the same samples by the original OPLS-DA model (more details in Additional file 1: Figure S2). The O2PLS-DA model was only defined on Metabolon measured data.

#### Association analysis of PCs from mature dates with date (soft/dry) type, country of production, ripening state and color

**Fig. 2:**
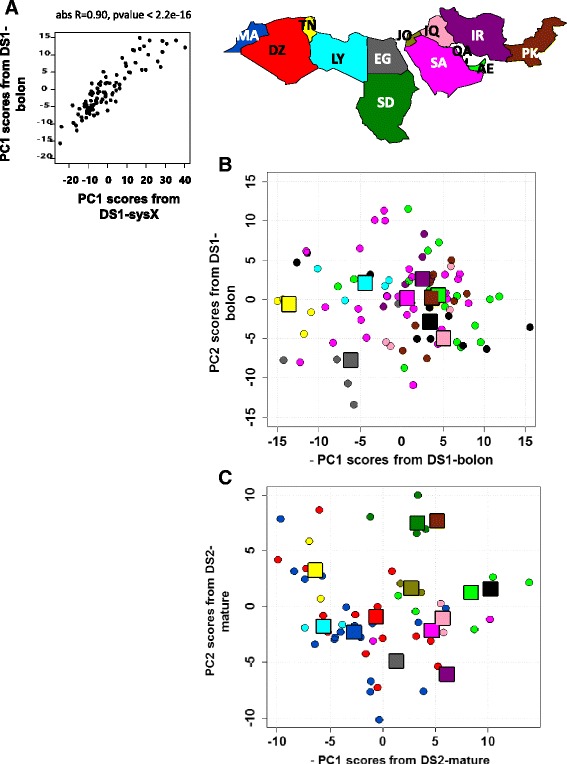
PCA analysis of metabolomics data from mature dates. **a** PC1 scores from DS1-bolon and DS1-sysX are highly concordant. **b** & **c** PC1 scores plotted against PC2 scores for DS1-bolon and DS2-mature respectively. The color of the circular symbols indicates the corresponding date sample country of production and follows the country-color code on the geographical map shown on the top of the figure. The square symbols were added to indicate the median PC1/PC2 coordinates per country and follow the same color code. Countries are denoted by their ISO Alpha-2 international code. The US unique date sample from the first collection has been omitted to keep the geographical map simple. PC1 scores have been negated so that the order of the countries follows that on the map (West/East left/right respectively). With both datasets, a significant association between PC1 scores and the country of production, expressed as an ordinal variable (refer to methods), was found. PC2 from both datasets showed no significant association

The lm function from the statistical analysis R software version 3.1.1 was used to run the regression model ‘*PC ~ date_variable*’ where date_variable consisted of one of four variables: date_type, a categorical variable with two levels: Soft and dry, with semi-dry varieties assigned to the dry class (Additional file 2); date_country, an ordinal variable from ranking the sampled countries West to East; date_ripening_state corresponding to the class prediction scores calculated by the OPLS-DA and O2PLS-DA models for samples from the first and the second collection respectively and date_color, a continuous variable based on the average of the red/green/blue (RGB) color measurements. The R package *maps* was used to generate the geographical map in Fig. 2 depicting the dates countries’ of production.

#### Analysis of the distribution of classes of metabolites on the loading space underlying PCs from mature dates

In order to further characterize PC1, the distribution of metabolites classified into broad metabolic categories including amino acid metabolism, sugar metabolism, energy metabolism, lipid metabolism, purine and pyrimidine metabolism, secondary metabolism and vitamin metabolism was manually examined on the underlying loading value space. The latter refers to the set of loading values assigned to the metabolites by PCA analysis where each loading value expresses the correlation between the corresponding metabolite abundance profile and the PC scores. Within a broad metabolic class, sets of metabolites sharing a functional or structural feature and having comparable loading values were identified. The common feature consisted mostly of pathway co-membership, a common catalytic activity or a unifying structural theme. These sets of metabolites were mapped to subclasses within the original broad categories as follows:

##### Amino acid metabolism

Refined into 1) subclass *amino acids* that includes proteinogenic and non-proteinogenic amino acids, 2) subclass *primary amines* deriving from direct decarboxylation of amino acids, 3) subclass *dipeptides* from pairs of amino acid conjugates, 4) subclass *glutathione cycle and glutathione metabolism* featuring both oxidized and reduced forms of glutathione, metabolites analogous to glutathione and gamma-glutamyl amino acid intermediates in the glutathione synthesis and degradation pathway, 5) subclass *N-acetylated amino acids*, 6) subclass *polyamines and polyamine degradation*.

##### Sugar metabolism

Refined into the following subclasses: 1) subclass *non-reducing sugars* featuring sucrose and sucrose like sugars, 2) subclass *reducing sugars and derivative alcohols, lactones and acids*, 3) subclass *TCA cycle* encapsulating di and tri carboxylic acid intermediates, 4) subclass *glycolysis* capturing phosphorylated sugars as well as key product pyruvate and derivative lactate, 5) subclass *sugar dehydration* encompassing products from dehydration of fructose and glucose.

##### Lipid metabolism

Within which the following subclasses were recognized: 1) subclass *lysophospholipids*, 2) subclass *lysophospholipid degradation* featuring free head groups and remaining lysophosphatidic acids or alternatively phosphorylated head groups and remaining monoacylglycerols in addition to N-acylethanolamine derivatives of lysophospholipids [31]*,* 3) subclass unsaturated fatty acid and oxylipins, 4) subclass *sphingoid bases*.

##### Purine and pyrimidine metabolism

Was split into two subclasses spanning each a different range of loading values: 1) subclass *nucleic acid and tRNA nucleosides* encapsulating simple forms of nucleobases and DNA/mRNA nucleosides as well as nucleosides carrying more complex tRNA specific modifications. Products from nucleoside modifications known to occur in mature eukaryotic rRNA [32] displayed a disparate range of loading values and were captured under 2) subclass *rRNA nucleosides.*

##### Secondary metabolism

Three clusters of metabolites were observed on the loading space consisting of: 1) subclass *tannins*, 2) subclass *general phenylpropanoid pathway* featuring a range of chalcone derivative flavonoids, excluding tannins, as well as precursor hydroxycinnamates and other derivatives, 3) subclass *poly-methoxycinnamates, hydroxybenzoates and volatiles (VOCs)* comprising di and tri-methoxycinnamates, hydroxybenzoates potential derivatives of methoxycinnamates [33] and volatiles deriving from both precursor and product molecules.

##### Vitamin metabolism, hormone metabolism and energy metabolism

These were small classes that did not require further refinement.

Finally, a general category *degradation activity and amino acid volatiles (VOC)* was formulated to capture metabolites from degradation of purines, vitamins and amino acids leading to synthesis of short chain volatiles (VOCs) [8]. For the rest of the article, all afore mentioned subclasses of metabolites as well as unrefined categories *vitamin metabolism, hormone metabolism, energy metabolism* and *degradation activity and amino acid VOC* will be collectively referred to as ‘metabolite classes’. It is important to note that the analysis was restricted to Metabolon measured data.

## Results

### Date fruit metabolomics datasets and platform comparison

In this study, mature date fruits were collected in two separate occasions from 14 different countries including: Morocco, Algeria, Tunisia, Libya, Egypt, Sudan, Jordan, Saudi Arabia, Iraq, Qatar, United Arab Emirates, Iran, Pakistan and the United States. Unlike dates from the second sample collection, date fruits from the first sample collection were measured by both MetaSysX and Metabolon, which led to two metabolomics datasets DS1-sysX and DS1-bolon, respectively. Overall, MetaSysX showed a relatively higher median RSD (refer to methods for details on RSD calculation) over biological replicates: 0.35 as opposed to 0.26 from Metabolon (Table 1-B). A parallel analysis based on calculating the average Euclidean distances ‘*AVED*’ between all metabolite measurements in a given sample ‘***s***’ and their corresponding counterparts in every other sample in the dataset revealed that the AVED between ***s*** and its biological duplicate has often the lowest value with both datasets (Additional file 1: Figure S3). This implies that even though the MetaSysX measurement was slightly noisier than the Metabolon measurement, as revealed by the RSD values from above, with both platforms variation between the date samples was still higher than the intrinsic variation between individual fruits from the same sample. The median RSD from technical replicate measurements of pooled batch 1 samples by Metabolon was as low as 0.12 (Table 1-B). Further to data reproducibility, it was noted that DS1-sysX is characterized by a higher level of metabolite missingness across samples, in particular with the lipid platform (Table 1-B). On the other hand, DS1-sysX featured a much higher number of detected signals in comparison to DS1-bolon (3143 as opposed to 282, Table 1-B) since MetaSysX performed an untargeted peak extraction. Also, complex lipids could only be obtained from MetaSysX measurement.

Comparison of Metabolon-measured data from dates from the first and the second sample collection (DS1-bolon and DS2) revealed a higher number of metabolites detected in the latter than the former dataset (Table 1-B). This could be primarily caused by the fact that the first sample set was initially processed by MetaSysX whereas the second sample set was processed solely by Metabolon and was matched against an updated library (refer to Additional file 3). Also the inclusion of dates from pre-ripening stages in the second set could have led to the detection of new metabolites. A range of secondary metabolites was detected in both datasets, in particular members of the general phenylpropanoid pathway including flavonoid species tannins, flavones, flavanonols, flavonols, flavanones, glycosylated flavanones and glycosylated flavonols as well as hydroxycinnamates, methoxycinnamates, lignans, monolignols and stilbenes (Table 3); though, the vast majority of detected metabolites were primary metabolites. These ranged from amino acids, lipids, sugars, vitamins, alcohols, acids, amines, purines and pyrimidines and will be covered in more details in the discussion section. The number of metabolites exclusive to DS1-bolon is 53 whilst 173 metabolites were only detected in DS2; 229 metabolites were measured in both datasets making the total number of unique metabolites detected over both datasets by Metabolon equal to 455.

**Table 3:** Count of different species of secondary metabolites in DS1-bolon and DS2

Secondary metabolite class	Secondary metabolite subclass	DS1-bolon	DS2
Fatty acid esters		1	1
Branched-chain amino acid volatiles		11	10
Flavonoids	Flavan-3-ols	1	1
	Flavanones	1	1
	Flavanonals	1	0
	Flavones	2	2
	Flavonols	1	1
	Glycosylated flavanones	1	1
	Glycosylated flavones	2	2
	Glycosylated flavonols	4	4
	Proanthocyanidins	1	2
Other phenyl propanoids	Cinnamic acids	8	8
	Lignans	1	2
	Monolignols	2	2
	Stilbenes	1	0
Other benzenoids		7	7
Terpenoids		2	6
Total		49	50

### PCA analysis of metabolomics data from mature dates reveals a first principal component associated with the geography of the region

In order to study the intrinsic variation in the composition of collected mature dates, PCA analysis was performed on measured metabolomics data using SIMCA (for details on QC preprocessing, the reader is referred to the methods section). With DS1-bolon, the top four components were found to be significant and together accounted for 41.1 % of the total variation in the dataset (PC1 accounted alone for 17.7 % followed by PC2 9.7 %, PC3 7.8 % and PC4 5.7 %). To validate these results, PCA was performed separately on the DS1-sysX metabolomics data measured from the same date samples. PC1 scores from DS1-bolon and DS1-sysX were highly correlated (abs Pearson R = 0.90, pvalue < 2.2e-16, Fig. 2a), confirming that the effect from PC1 is platform independent. Regressing PC1 scores from DS1-bolon against the date_country variable (defined in the methods section) revealed a significant pvalue = 4.80e-08 and an adjusted R-squared of 0.34. There was no significant association between the date_country variable and PC2, 3 and 4 from DS1-bolon.

In turn, PCA analysis of DS2-mature revealed 4 significant components accounting for 44.2 % of the total variation where 16.7 % was captured by PC1 alone and 11.4 %, 10 % and 6.06 % by PC2, PC3 and PC4 respectively. Similar to DS1-bolon, scores from PC1 alone were significantly associated with the ordinal date_country variable (pvalue = 3.14e-05, adjusted R-squared = 0.45). Taken together, these results suggest that PC1, explaining the largest systematic variation in mature dates from the first and second sample collection, is significantly associated with the fruit’s country of production. An increased density of the North African dates over the positive range of the PC1 scale opposed by an enrichment of the Gulf dates at the negative range can be observed with DS1-bolon and DS2-mature metabolomics datasets on Fig. 2b & c respectively.

### PC1 from mature dates captures varying extents of fruit ripening

**Fig. 3:**
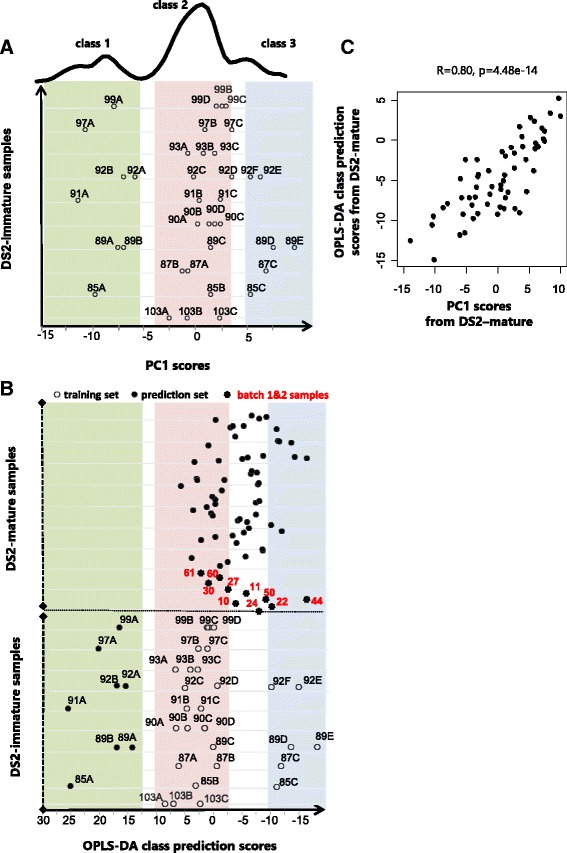
PC1 from DS2-mature is associated with the ripening process. **a** PC1 scores from DS2-immature. Fruits from varying stages of ripening from the same sample are shown on the same line. Each fruit is labelled with an identifier featuring a letter indicative of its extent of ripening relative to the other fruits within the sample as judged by eye. The ordering of the letters is well captured by the PC1 scores and occasional discrepancies occur when the fruits featured very similar PC1 scores. Density analysis of the PC1 scores, showing on top of (**a**) indicates that the fruits can be assigned to three developmental classes, denoted as class 1 (light green), class 2 (light pink) and class 3 (light blue) by increasing ripening maturity. **b** An OPLS-DA classifier trained on class 2 versus 3 was used to calculate class prediction scores for all DS2 samples including the *batch1&2* samples which *were* measured in separate batches once with dates from the first sample collection and again with dates from the second sample collection. **c** A scatter plot of PC1 scores and OPLS-DA class prediction scores from the DS2-mature samples indicates a significant correlation

The inclusion of a subset of date fruits with on-going ripening activity in the second sample collection (also referred to as DS2-immature, Fig. 1b & c) was aimed at identifying the metabolic signature of the ripening process. The objective was to assess possible contribution of the development effect to observed variance in DS1-bolon and DS2-mature as although the corresponding date samples were considered mature, fruits still undergoing ripening changes may have been incidentally present. An overview of the analysis used to assess this possible effect can be found in the methods section; here, we present the results. PCA analysis of the immature fruits revealed a high concordance between PC1 scores and fruit ranking previously defined based on visual assessment of the fruits’ ripening extent (refer to methods) (Fig. 3a). Occasional discrepancies were observed only when the fruits featured similar PC1 score values, which would suggest comparable ripening states. A density analysis of PC1 scores revealed three broad clusters of samples which were denoted by class 1, 2 and 3 by increasing extent of ripening (Fig. 3a). An OPLS-DA model trained on class 2 versus 3 revealed one significant predictive component explaining 87 % of the variation in the class variable (R-squared-Y = 0.87, Q-squared = 0.69). This classifier essentially learns the metabolites best differentiating between the classes. Applying this classifier to all samples in DS2 excluding the training set led to class prediction scores that reflect the original levels of such differentiating metabolites in these samples. It follows that these scores are indicative of the extent of ripening in these samples. Examination of these prediction scores revealed two main observations: First, DS2-immature samples from class 1 were laid correctly closest to class 2 and furthest from class 3; second, DS2-mature date samples were positioned expectedly in between class 2 and 3 (Fig. 3b). A significant Pearson R value (R = 0.80, pvalue = 4.48e-14) was obtained from comparison of the OPLS-DA class prediction scores and their PC1 counterparts from DS2-mature samples (Fig. 3c). This implies that further to the geography effect, PC1 from DS2-mature also carries a ripening signature. No significant association was found with PC2, 3 and 4.

**Fig. 4:**
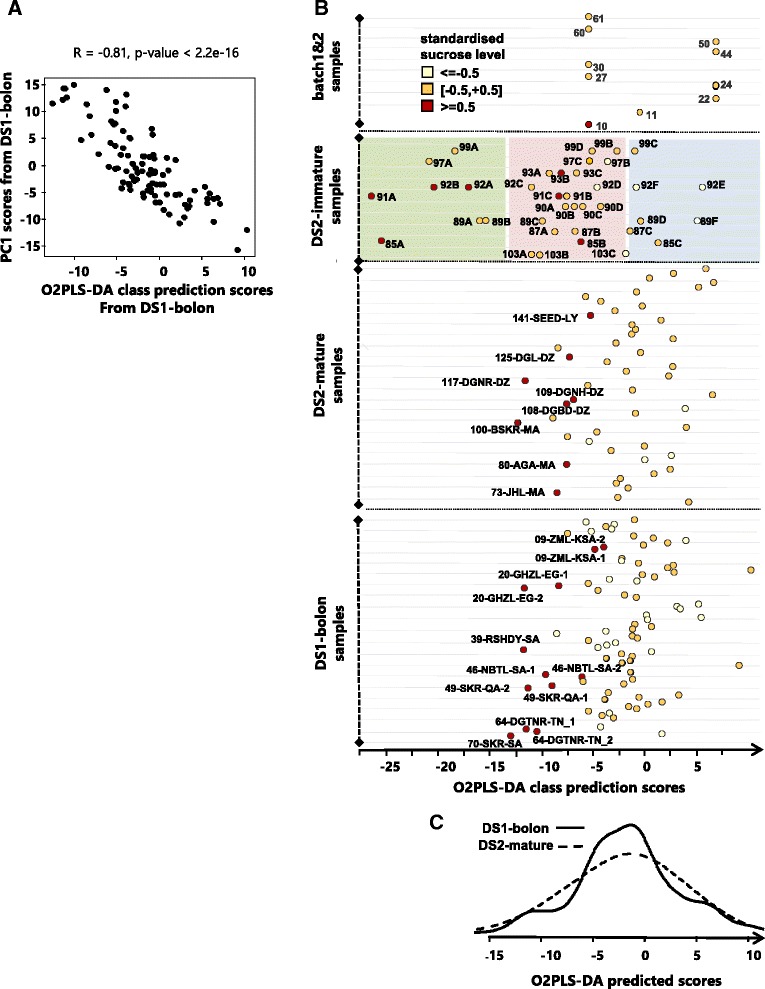
The O2PLS-DA model for predicting the ripening states of DS1-bolon samples. **a** A scatter plot of the O2PLS-DA predicted scores versus the PC1 scores from DS1-bolon indicating a significant correlation level. **b** The O2PLS-DA class prediction scores (x-axis) for all 186 measured metabolic profiles listed sequentially on the y-axis within their respective datasets. The batch1&2 samples served (excluding sample 11) as the training set for the O2PLS-DA classifier. The DS2-immature samples are predicted correctly within their predefined development classes as initially revealed by the PCA analysis: class1 (light green), class2 (light pink), class3 (light blue). The symbols color code reflects the level of the dates endogenous sucrose level expressed in standard deviation units from the mean, calculated for each batch separately. Only samples with high sucrose level are labelled with their IDs for clarity. **c** Density plot of the O2PLS-DA class prediction scores for the DS1-bolon and DS2-mature datasets

The procedure for mapping the ripening effect onto DS1-bolon was outlined in the methods section. Briefly, it followed from examination of the class prediction scores by the OPLS-DA classifier (Fig. 3b) that the 10 samples measured in both batch measurements (or *batch 1&2 samples*) are spread over class 2 and 3 (the word batch here referring to a sample collection set). These samples served to construct seed classes 2 and 3 for a new classifier. The latter was based on the O2PLS-DA procedure which is able to dissect the common signal from multiple measurements of the same samples that consistently distinguishes between the samples’ classes. In this work, the multiple measurements of the training set samples consisted of their batch1 and 2 metabolomics measurements. The class segregation of this training set was guided by the results on Fig. 3b and tuned to maximize the concordance level between derived class prediction scores for a subset of batch 2 samples and their counterparts by the OPLS-DA classifier (more details in the methods and Additional file 1: Figure S2). The O2PLS-DA model with the best concordance level was found to consist of batch 1&2 samples 61, 30, 60, 27, 10/24, 50, 22, 44 affiliated to seed class 2/seed class 3 respectively whilst leaving out sample 11 (Additional file 1: Figure S2). The O2PLS-DA class prediction scores for DS1-bolon were found to correlate strongly with their PC1 score counterparts (abs Pearson R = 0.8, pvalue < 2.2e-16, Fig. 4a). This implies that PC1 from the first collection of dates is also associated with a ripening effect further to the geography of the region, in a similar way to PC1 from the second collection samples. No significant association was found with PC2, 3 and 4 from the same dataset.

Importantly, the O2PLS-DA class prediction scores for the first and second collection of date samples are comparable and can be projected along the same axis as shown in Fig. 4b. This led to the following interesting observations: First, sample 11 from batch 1&2 samples was predicted in between class 2 and 3, in accordance with the original OPLS-DA classifier in Fig. 3b. Second, the ranking of the fruits from DS2-immature was well maintained by the O2PLS-DA classifier and occasional errors are similar to those observed with the OPLS-DA classifier in Fig. 3b. These two observations further confirm the validity of the O2PLS-DA model. Third, samples from the second collection of dates appeared more spread out than the samples from the first collection, which show some density in the middle area between class 2 and 3, also reflected by the density plot in Fig. 4c. This could be due to the more controlled post-harvest conditions with marketed dates which were dominant in the first date collection. Last, with both DS1-bolon and DS2-mature, date samples closer to class 2 appeared to contain a relatively high level of sucrose. Apart from 141-SEED-LY annotated as a soft type (Additional file 2), all other varieties with high sucrose levels and known type belonged to the dry or semi-dry type. High sucrose levels were also observed with the DS2-immature fruits from classes 1 and 2 (Fig. 4b).

### The metabolic space underlying PC1 from mature dates is consistent with the biology of fruit ripening

**Fig. 5:**
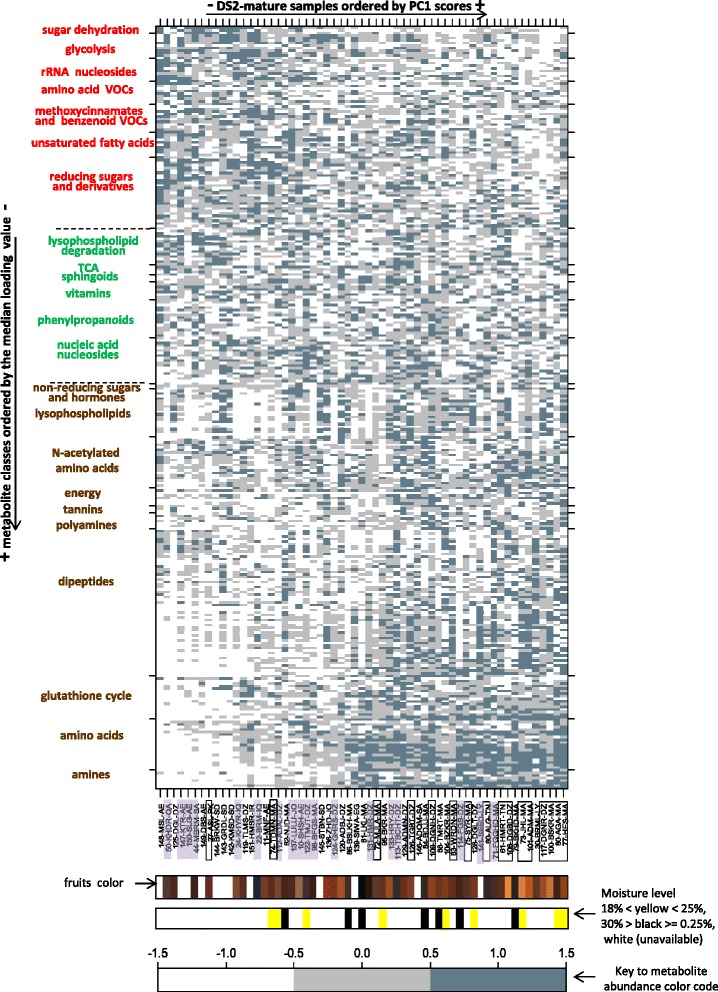
Heatmap analysis based on DS2-mature data. Showing the abundance level of metabolites arranged in biological classes by increasing PC1 loading values (y-axis) along date samples arranged by increasing PC1 scores (x-axis). Metabolite classes are shown to the left in different colours to reflect various biochemical phases of the ripening process in dates: (brown) early ripening Khalal, (green) ripening underway corresponding to Rutab and (red) over-ripening. The positive range of PC1 shows increased discolouration amongst dates many of which belong to the dry type (black framed rectangles). The soft type (highlighted in purple rectangles) is enriched at the negative range. Information on the dry/soft phenotype is variety specific and was collected from the literature where possible. Low moisture fruits and relatively moist fruits appear randomly scattered along PC1

Twenty three classes of metabolites having a common structural or functional theme and comparable PC1 loading values were defined as described in the methods section. This grouping is rationalized by the fact that metabolites with strongly positive and strongly negative loading values are highly correlated ‘within’ but anti-correlated ‘in between’. Strong correlation at either end of the loading values range justifies the enrichment of biological classes of metabolites at both ends of the range. In parallel, there exists an intimate relationship between loading values and PC scores in that a metabolite loading value expresses the extent of correlation between the metabolite abundance profile and PC scores across the samples. The relationship between metabolite levels, PC1 loading values and scores in DS2-mature is captured in the heatmap on Fig. 5 and a similar figure for DS1-bolon can be found in Additional file 1: Figure S4. The x-axis features date samples ordered by increasing PC1 score values whilst the y-axis features metabolites ordered by two criteria: First metabolite classes were ordered by their median loading value then the metabolites were ordered by their loading values within each class.

**Fig. 6:**
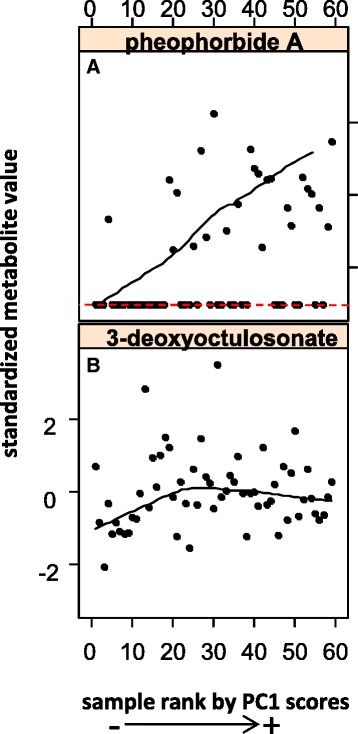
Standardized abundance levels of selected metabolites in DS2-bolon date samples ordered by PC1 scores. **a** Pheophorbide A, a marker of chlorophyll degradation. **b** 3-deoxyoctulosonate, a structural component of rhamnogalacturonan II species of pectin and a marker of cell wall hydrolysis. Samples with missing values were assigned a minimum value indicated by a red dashed line

Inspection of the heatmap on Fig. 5 shows a clear signature of the biochemistry of date ripening as previously outlined in the introduction section and additional details are consistent with the general fruit ripening process as shall be discussed later. Briefly, date samples with the most positive PC1 scores featured the highest levels of amines and regulatory polyamines, glutathione-mediated antioxidant activity, energy production, lysophospholipids, amino acids, tannins, non-reducing sugars and hormones. The enrichment patterns of the last three classes of metabolites further to a similar pattern by pheophorbide A (Fig. 6a), a degradation product of chlorophyll, are consistent with the biochemical profile of the Khalal early ripening stage in dates during which fruits ungreen and acquire color (refer to background for more details). The abundance level of all previously mentioned metabolites declined in date samples with middle range PC1 scores. This is unlike metabolites from the general phenylpropanoid pathway, nucleic acid nucleosides, vitamins, TCA intermediates, sphingoid bases and lysophospholipid degradation products which maintained a steady abundance level. The enrichment in keto-octulosonic acid from the degradation of cell wall pectin with middle range PC1 scores (Fig. 6b) may indicate increased fruit softening which is typical of the Rutab advanced ripening phase in dates (refer to background). Dates with very negative PC1 scores showed enrichment in unsaturated fatty acids, aroma volatiles from degradation of amino acids and phenylpropanoids, reducing sugars and sugar dehydration products. The latter can also derive from the Maillard reaction [34]; consistent with the advanced ripening stage in dates (refer to background). Accumulation of glycolysis sugars and products from degradation of ribosomal structure could be indicative of a slowing down in metabolic activity in fruits at this stage. Similar enrichment/depletion patterns of metabolite classes along PC1 were observed with DS1-bolon data (Additional file 1: Figure S4), with a marginal discrepancy in phospholipid metabolism.

Interestingly, date varieties obtained from different countries showed similar PC1 values and hence comparable ripening-related biochemical profiles. Examples are Deglet Nour date samples 117-DGNR-DZ and 64-DGTNR-TN from Algeria and Tunisia, respectively, at the positive end of PC1; Sufri date samples 41-SFR-SA and 52-SFR-QA from Saudi Arabia and Qatar, both with middle range PC1 values and Mabroom date samples 44-MBRM-SA and 48-MBRM-QA from Saudi Arabia and Qatar at the negative end of PC1 (Fig. 5 and Additional file 1: Figure S4).

Close examination of the range of measured moisture values from a handful of samples from the second sample collection (refer to methods) suggested the presence of moist Rutab dates (25 % < moisture level <30 %) amongst the cohort (Fig. 5). These may have been undergoing active ripening related changes and may not be considered mature dates, since their phenotype may have changed have they been allowed more time to complete their ripening (refer to methods for definition of date maturity). Their inclusion in the cohort was therefore incidental. Interestingly, dates where low moisture content (from the range [18–25 %] can be taken to suggest a stationary metabolic activity and hence maturity were also observed at the positive range of PC1 (Fig. 5) that captures early ripening metabolic activity. This indicates that the mature population of dates is characterized by a varying extent of ripening metabolic turnover. Further analysis suggested an enrichment of the dry and semi-dry types of dates as well as increased fruit discoloration amongst the mature dates at the positive range of PC1 whilst the soft type is over-represented at the negative range (Fig. 5 and Additional file 1: Figure S4) (based on association analysis between PC1 scores and the date_type and date_color variables that revealed significant pvalues 6.063e-09/0.05 and 0.018/0.002 for DS1-bolon/DS2-mature respectively).

**Fig. 7:**
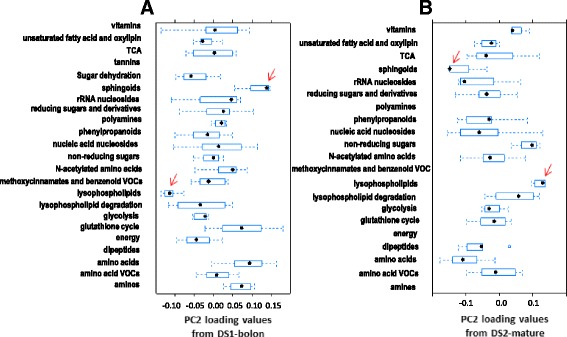
Boxplots of PC2 loading values arranged by metabolic class. **a** DS1-bolon, **b** DS2-mature. The classification of metabolites follows that developed for PC1 (refer to methods). For both datasets, metabolite classes *sphingoids* and *lysophospholipids* (pointed at with a red arrow) appeared to underlie the effect captured by PC2. Classes with less than three metabolites were not considered; these consisted of tannins and dipeptides for DS1-bolon and polyamines, methoxycinnamates and benzenoid VOCs, energy and amines for DS2-bolon. The star in each box indicates the median loading value per metabolic class

The same classes of metabolites were re-evaluated in light of the loading values from PC2, 3 and 4 from analysis of DS1-bolon and DS2-mature. For each component and dataset, box plots of the loading values arranged by metabolite class can be found in Fig. 7 and Additional file 1: Figure S5. Interestingly, PC2 from the two datasets appeared to carry the same metabolic signature with the *sphingoids* and *lysophospholipids* classes observed at opposite ends of the loading values range (Fig. 7). Moreover, the absolute correlation in the per-metabolic class median of the PC2 loading values between the two datasets was found equal to 0.68. No concordance was found between the PC3 and PC4 per-class median loading values across the two datasets implying that PC3 and PC4 capture metabolic effects that are intrinsic to each dataset. For DS1-bolon, PC3 captured an opposing effect between the metabolic class *TCA* and two other classes: *non-reducing sugars* and *N-acetylated amino acids* whilst PC4 highlighted a contrast between class *TCA* and class *unsaturated fatty acids and oxylipins* (Additional file 1: Figure S5). As for DS2-mature, an opposing trend was noted between metabolic classes *unsaturated fatty acid and oxylipins* and *rRNA nucleosides* for PC3 and between *phenylpropanoids* and *glycolysis* for PC4.

## Discussion

### The ripening process may be completed to varying extents in mature soft and dry types of dates causing major variation in fruit composition

In this study, two collections of date fruits were measured with metabolomics techniques and multivariate statistical analysis applied to both extract and characterize the principal components that explain most variability in their metabolomics data. The two date collections present fundamental differences: Dates from the first sample collection originated mostly from the Gulf region and the subset that was obtained from shops is likely to have been through a grading and drying process to meet market criteria. In contrast, dates from the second collection were mostly North African varieties collected fresh from the trees and local markets. Despite these differences, PC1 separately derived for each dataset was found in both cases significantly associated with the fruit country of production with dates from the East Gulf region and the West North African side being observed, in broad terms, at opposite ends of the PC1 scale. Another factor that proved to be strongly associated with PC1 is the extent of metabolic conversion during the ripening process. The moisture analysis indicated that this is partly explained by the incidental occurrence of immature moist fruits which possibly had not yet completed their ripening. This was rather anticipated and was the reason for the inclusion of a development stage dataset as part of the second cohort. However, low moisture dates which should not incur any further changes in their phenotype and are by definition mature, were found to span the entire range of PC1 displaying varying extents of ripening metabolic turnover. In other words, some date samples reach maturity after a rigorous ripening activity (negative range of PC1) whilst others become mature and dry out having carried out a lower extent of metabolic turnover (positive range of PC1). Further analysis suggested that the former dates are generally from the soft type whilst the latter are mostly from the dry type. The soft/dry phenotype is variety specific and was, in this study, collected from the literature. It is important to note that the phenotype in question does not refer to a development or maturity stage per-se but to a collection of physiochemical properties that distinguish the fresh naturally ripened fruit from the two types: Dates from the soft class are characterized by higher moisture, softer texture and higher levels of sucrose to reducing sugars in the ripe fruit (refer to background for more details). Due to their high moisture level, dates from the soft class often necessitate additional drying to become mature unlike the dry type which matures naturally on the trees.

Importantly, the observed variation in metabolic turnover in dates from the soft and dry types can explain their known phenotypic differences. Low metabolic turnover in the dry type may limit the synthesis of color substances and efficient degradation of fibrous structures. This could explain fruit discoloration and the hard texture typical to this type of date [4]. On the other hand the high metabolic turnover with the soft type is likely to be accompanied by optimal degradation of fibers and accumulation of color molecules, which justifies the soft texture and intensified color typical to this type of dates. Also, the high sucrose versus high reducing sugar levels in dry versus soft types of dates can be attributed to the development effect since early accumulating sucrose is readily broken down into reducing sugars as ripening progresses. Interestingly, hydrolysis of sucrose by invertase during ripening was found to display faster kinetics in soft than in the dry type of dates [35]. Slower degradation of sucrose could be a limiting factor for the ripening process in dry varieties as it would impact a significant proportion of downstream ripening reactions. Whether the activity of invertase is alone causative of the soft/dry classification of dates remains to be investigated. Also, underlying factors whether of genetic nature or simply consisting of low water activity or both need to be addressed; as although evidence in the literature suggests genetic diversity between the soft and dry types [17], a direct link to the fruit ripening process remains to be established. One venue for investigating the role of water activity is via experimental modification of water content in developing soft and dry dates through altering irrigation amount and frequency [36, 37]. This combined with global metabolome characterization of a larger cohort of dry and soft dates may provide important clues on the relevance of water activity to the soft and dry phenotypes in dates. In summary, the ripening effect captured in PC1 in this study is not exclusive to fresh immature dates with ongoing ripening activity but also mature dates from the dry/soft classes that display varying extent of metabolic turnover during ripening.

Importantly, the enrichment of dry and soft type of dates at opposite ends of PC1 provides an explanation for observed association between PC1 scores and geography. In the Arab world, different types of date palm cultivation areas with varying climates tend to be more suitable for either type of dates: Oasis sites typical to North African countries including Tunisia, Morocco, Algeria, Libya and Egypt are famous for the semi-dry and dry types of dates whilst offshore dry areas found in Egypt, Sudan, Libya, Saudi Arabia and Oman are mainly suitable for dry varieties. Finally, the humid nature of coastal areas typical to Bahrain, United Arab Emirates and Qatar are more suitable for soft varieties of dates [15, 16]. Importantly, the established genetic variation in dates between the North African and Arabian Gulf regions [38] could be linked to varying climatic conditions imparting a bias in the type of cultivar between the two regions.

In comparison to PC2, 3 and 4, PC1 captures a higher proportion of the variance in the data. Also, in this work, unlike PC2, 3 and 4, PC1 is significantly associated with available phenotypic characteristics of the dates including the country of production, soft/dry type and color intensity. This justifies its being at the focus of this study. Nevertheless, the metabolic signatures of PC2, 3 and 4 will be later discussed in some details.

### Multivariate techniques are useful exploratory and integrative tools of single and multi-measured metabolomics datasets

In this study, a range of multivariate techniques were used to reach a comprehensive understanding of determinants of metabolic variation in date samples. Initially, non-supervised PCA was used to extract this variation. In order to assess the relationship with the ripening process, an OPLS-DA classifier was trained on the DS2-immature dataset to model the ripening process in dates. However, a prerequisite for the OPLS-DA model is class segregation of samples, which was clearly missing with this dataset. This is because the fruits in DS2-immature were not collected at pre-set time intervals during the ripening process and therefore could not be aligned across samples to create the required classes. Instead, a PCA analysis revealed a dominant PC1 that essentially captured the ripening process in DS2-immature and organized the constituent fruits accordingly into three broad clusters. Clusters 2 and 3 served as the training set for the OPLS-DA model leaving out cluster 1. This is rationalized by the anticipation that the prediction set, consisting of DS2-mature, would lay between clusters 2 and 3 as cluster 1 featured green dates from the early phase of ripening that was not represented in the prediction set. Mapping the development effect onto DS1-bolon from the first date collection was important for the sake of replicating the association between PC1 and the biochemistry of fruit ripening in a yet independent dataset. The model used was based on the O2PLS-DA procedure which is able to extract systematic variation from batch 1 and 2 measurements of the training set that consistently differentiate the designated sample classes. It follows that the O2PLS-DA procedure was used in this study to consolidate separate batch measurements of the same samples as although the measuring technique was essentially the same, slight operational changes may have been introduced between the two batch measurements which were well separated in time. This is, in principal, similar to the way the technique has been traditionally applied to bring together measurements of the same biological samples by different analytical methods [30].

### Comprehensive characterization of temporal aspects of ripening metabolism in dates

At the metabolic level, PC1 has a ripening signature and is the reason why it is able to differentiate between the dry and soft phenotypes that undergo varying ripening kinetics. Soft and dry types of dates have different climatic requirements which could explain the association between PC1 and geography. We now focus on the metabolic signature of PC1 and dedicate the remaining part of the discussion section to contrasting observed enrichment in classes of metabolites along PC1 with the known biochemistry of fruit ripening (though, we will occasionally refer to other PCs when discussing metabolic classes that are relevant to them). We would implicitly refer to the positive and negative ends of PC1 by their corresponding ripening profiles as early ripening and late ripening based on the results on Fig. 5. We will frequently refer to Additional file 6 which shows scatter plots of all metabolite abundance profiles along PC1 organized within their respective biological classes.

#### Amino acids and related metabolites

Enrichment in free amino acids was observed in this study in dates with an early ripening profile similar to other fruit [8, 39] (Fig. 5). Amongst all detected amino acids, the levels of alanine, glutamate and aspartate declined least in dates with late ripening profile (Additional file 6), consistent with previous work looking at ripening in tomato [39]. In general, amino acids serve as building blocks for synthesis of key intermediates and end-products of the ripening process in fruits [8]. In particular the aromatic amino acids, also measured in this study, give rise to a myriad of secondary metabolites, notably color and flavor-conferring phenylpropanoids. The observed enrichment in dipeptides in date fruits with an early ripening profile in this study (Fig. 5) may be linked to protein degradation activity recruited by hormones to eliminate pre-ripening enzymes at the onset of ripening [8]. Another potential source for the dipeptides is the targeting peptide sequence, attached to pre-folded nuclear proteins, which is digested upon protein entry into organelle structures including chromoplast [40]. Chromoplasts, which are differentiated forms of chloroplasts lacking chlorophyll, act as metabolic hubbs at early ripening, necessicating constant in-flow of effector proteins from the nucleus [41]. Targeting peptide sequences contain mostly hydrophobic amino acid residues [40], consistent with the high proportion (70 %) of hydrophobic valine, leucine, isoleucine and phenylalanine amongst amino acid constituents of the dipeptides observed in this study. Interestingly, N-acetylation of chromoplast-targeted pre-folded proteins was suggested as a mechanism of organelle specificity [42]. This may account for the enrichment of N-acetylated amino acids in date samples with an early ripening profile in this study (Fig. 5); although acetylation can sometimes be a necessary intermediate reaction during metabolism of amino acids. Finally, enrichment in glutathione activity in dates with an early ripening profile (Fig. 5) is consistent with increased antioxidant activity in fruits at early ripening [43].

#### Primary amines and polyamines

In this study, ethanolamine, GABA, serotonin, tyramine, tryptamine and phenethylamine from the decarboxylation of serine, glutamate, 5-hydroxy tryptamine, tyrosine, tryptophan and phenylalanine were all found enriched in dates with early ripening profiles (Additional file 6). This is consistent with early ripening expression of amino acid decarboxylases leading to amine synthesis in a number of fruits [44, 45]. Tyramine and tryptamine serve as precursors for the synthesis of defense-mediating alkaloids previously detected in dates [46] and a turnover of phenethylamine into antipathogen volatiles phenylacetaldehyde and phenylethanol serves the same purpose [8, 45]. The role of serotonin in fruit ripening has not been fully investigated; however, melatonin that derives from N-acetylserotonin (a derivative of serotonin also observed in our data), has recently been found to promote various physiological aspects of ripening when given exogenously to green tomato [47]. Recently, the decrease in GABA with ripening was linked to maintaining high levels of essential glutamate and aspartate during tomato ripening [48]. Enrichment in the polyamine putrescine in dates with an early ripening profile (at the positive end of PC1) is consistent with previously reported expression of a mouse ornithine decarboxylase conjugated to a ripening specific promotor at the onset of ripening in transgenic tomato [49]. Previous work suggested a synergy between the ripening hormone ethylene and the polyamines spermine and spermidine, derivatives of putrescine [39, 50]. The potential regulatory role of putrescine may justify its co-occurrence with products from its degradation pathway in dates with an early ripening profile (Additional file 6).

#### Secondary metabolism

The earliest sign of secondary metabolites from the phenylpropanoid pathway in our data consisted of tannins procyanidin B1 and procyanidin B2 and catechin monomers all showing maximal level in dates with an early ripening profile (Additional file 6), in accordance with the literature [8, 51]. Astringent tannin oligomers are abundant in green fruit and only lose their astringency when undergoing structural changes as ripening progresses [52]. In addition to tannins, a wide range of color and flavor flavonoids and hydroxycinnamates were observed to peak at different ranges of PC1 and some showed no correlation with PC1. In general, variance from these metabolites was poorly explained by PC1 (Additional file 6). This could be due to a much stronger influence by genetic background [8], which may contribute to unique taste and color characteristics of individual varieties. Interestingly, PC4 from DS2-mature revealed an opposing trend between classes *phenylpropanoids* and *TCA on* one hand and the accumulation of phosphorylated sugars, captured under the class *glycolysis,* on the other hand. This effect can be explained by energy requirement for the synthesis of phenylpropanoids through initial degradation of phosphorylated sugars during glycolysis and downstream TCA activity. A third class of secondary metabolites consisted of volatiles, major contributors to aroma in fruits. In this study, an increase in branched chain amino acid derived volatiles and hydroxycinnamate derived volatiles was observed in dates with a late ripening profile (Fig. 5 and Additional file 6), consistent with the literature [8]. Volatiles are strong attractants of seed dispersers and their sharp increase in overripe fruit could constitute a mechanism to maximize the chance of consumption before onset of senescence.

#### Changes to cell wall and cell membrane

Alterations in cell membrane composition have long been known to occur during ripening [53–55], but have been given little attention by the more recent literature. Key changes to membrane phospholipids during ripening include an increased desaturation level of fatty acyl chains facilitating their peroxidation. Induction of expression of a handful of desaturase isomers was found to be strongly associated with a continuous flux of linoleate and linolenate substrates of the lipoxygenase (LOX) pathway during peach ripening [56]. This pathway is known for being the mechanism of synthesis of a myriad of C6 volatile aldehydes and alcohols that contribute significantly to fruit aroma [8]. In this study, a range of mono and poly-unsaturated fatty acids including the LOX substrates and their oxidized derivative oxylipins were observed to generally plummet in dates with a late ripening profile at the negative range of PC1.

Initial excision of fatty acyl chains resulted in accumulation of lysophospholipids in dates with an early ripening profile and late accumulation of lysophospholipid degradation products in dates with a late ripening profile (Fig. 5). These included monoacylglycerols and phosphorylated head groups, free head groups and remaining lysophosphatidic acids [57] as well N-acylethanolamines, derivatives of lysophospholipids via N-acylphosphatidylethanolamine intermediates [31] (Additional file 6). These degradation products, in addition to sphingoid bases, are likely to have a signaling role in fruits and some are already known to be downstream mediators of abscisate signaling in other plant organs [58–60]. The opposing trend in lysophospholipids versus sphingoids by PC2 in both datasets is interesting and may suggest a change in signaling patterns during ripening of certain date varieties or as a response to certain external stimuli. For instance, sphingolipid signaling is known to be induced under drought conditions in plants [59].

Fruit softening during ripening is known to be associated with an increased activity of fiber degrading enzymes, many of which targeting cell wall structures [8]. Pectin is a major constituent of the plant cell wall and its hydrolysis seems to make way for synergistic disassembly of cellulose and hemicellulose polysaccharide matrices [8]. In this study, the abundance of keto-deoxyoctulosonic acid, an acidic monosaccharide located in the side chain of the rhamnogalacturonan II class of pectin peaked in dates with middle range PC1 (Fig. 6b). This served to establish a link to the Rutab phase which is characterized by maximal fruit softness (refer to results).

#### Sugar metabolism, energy and gene expression activity

In this study, sucrose and related non-reducing sugars kestose and melezitose (from DS1-bolon) were most abundant in fruits with an early ripening profile at the positive range of PC1 (Fig. 5, Additional file 6). In fruits, an increase in sucrose levels is observed at the late mature green stage and sucrose is broken down by the enzyme invertase following the onset of ripening [8]. Metabolism of released sugar monomers proceeds via the early pentose phosphate pathway, which serves to provide carbon precursor molecules for the synthesis of aromatic amino acids, secondary metabolites, vitamins and purine/pyrimidines. In parallel, flux through glycolysis and the downstream TCA cycle serves to sustain energy levels required for gene expression and anabolic reactions during ripening. The relationship between precursor non-reducing sugars and product TCA intermediates is captured by PC3 from DS1-bolon and may reflect varying glycolysis/TCA kinetics across the date cohort.

In this study, the abundance levels of energy molecules NAD+ and AMP were lowest in dates with a late ripening profile (Fig. 5, Additional file 6). This together with accumulation of the TCA cycle intermediate fumarate, members of the glycolysis pathway and ribosomal nucleosides possibly originating from ribosome degradation (Additional file 6) may indicate a diminished metabolic activity at this late stage of ripening. Interestingly, metabolites from the *TCA*/*rRNA nucleosides* classes appeared to contrast the *unsaturated fatty acids* and *oxylipins* in PC4/PC3 from DS1-bolon/DS2-mature respectively. Late synthesis of oxylipins in some species of dates may require residual TCA activity and late synthesis of key enzymes.

The accumulation of reducing sugars in dates with a late ripening profile in this study (Fig. 5) confirmed similar reports in dates at the late Tamr stage [10]. Amongst the sugars observed (also shown in Additional file 6) xylose, fucose, arabinose and glucose may derive from cell wall hydrolysis activity during ripening. Ribulose and xylulose could derive from their phosphorylated forms from the pentose phosphate pathway whilst sugar alcohols, sugar lactones and derivative acids may result from upregulation of aldose/ketose oxidoreductases in ripe dates, previously shown using a proteomics approach [61]. It is important to note that besides contributing to fruit flavor, sugar alcohols are a type of polyol osmoprotectant that may act to alleviate the impact of fruit dryness at this stage.

#### Vitamins and hormones

In this study, a range of vitamins has been detected including riboflavin, niacin, pyridoxine and nicotinate (Additional file 6). The enrichment in pyridoxine in dates with an early ripening profile may be attributed to its essential role in amino acid synthesis and metabolism by amino acid decarboxylases at the early phase of ripening. In contrast, the accumulation of threonate (Additional file 6), a degradation product of vitamin C, in dates with a late ripening profile is consistent with the previously described decrease in vitamin C at the Tamr stage [1].

Dates behave like climacteric fruits implying a leading regulatory role by ethylene although interplay with abscisate may be operating on certain ripening events [8]. In this study, the ripening hormone ethylene was not measured (below the mass cut-off imposed on the instrumentation) but two related metabolites were observed (Additional file 6). One is cyanoalanine, which is a conjugate of cysteine and cyanide (cyanide being a toxic byproduct of ethylene synthesis [62]) and 5-methylthioadenosine, an intermediate in the Yang cycle that replenishes the ethylene precursor SAM. Both molecules showed maximum levels in dates with an early ripening profile at the positive range of PC1 (Additional file 6). This range was previously mapped to the Khalal stage (refer to results), which follows the climacteric ethylene peak in dates [63]. A similar pattern by isopentenyl adenosine, the precursor of the cytokinin hormone zeatin, is concordant with the reported abundance profile of zeatin during tomato ripening [64]. Abscisate is an early regulator that precedes ethylene in the climacteric fruit model tomato [65, 66]. Following the peak in ethylene, abscisate was shown to increase in abundance slightly [67], which could explain the small peak in abscisate observed in this study in dates with middle range PC1 values. In summary, key hormones and related metabolites were measured as part of this work motivating future analysis of partial correlations with other measured metabolites in order to uncover regulatory mechanisms operating on distinct aspects of the ripening process in date fruit.

## Conclusions

This study has shed light on important aspects of date fruit biology. It was shown that mature dates may significantly vary in their composition depending on the extent of metabolic conversion during the ripening process in line with the dry/soft classification of dates. It follows that the dry and soft types of dates present varying nutritional value and whilst the dry type is richer in amino acids, amines, phospholipids, energy molecules and sucrose sugar, the soft type contains more aroma volatiles, reducing sugars, sugar alcohols and acids, simpler lipids and may carry traces of dehydration products as a consequence of artificial drying. In addition to the ripening effect, the geography aspect was found to be associated with the main component of variation (PC1) in both cohorts. This was also linked to the dry and soft phenotypes based on the fact that the two types have varying climatic requirements. Our analysis of metabolite classes in dates with early versus advanced ripening metabolic profiles revealed similarity to ripening in other fruits and served to emphasize the changes in phospholipids that are not described in sufficient details in the current literature. The findings from this study were confirmed in two separate datasets and one dataset was measured by two independent metabolomics platforms to reveal identical effects. The reliability and reasonable cost of metabolomics technique may motivate further research on non-model fruits contributing to a broader understanding of various aspects of fruit biology.

## Availability of supporting data

Metabolomics datasets measured by Metabolon in this study are available in Additional file 4 & Additional file 5.

## Additional files

Additional file 1:
**Figure S1.** Phenotyping the date samples.** Figure S2.** Iterative optimization of the O2PLS-DA classifier. **Figure S3.** Quality control based on Metabolon/MetaSysX replicate measurements. **Figure S4.** Heatmap analysis based on DS1-bolon data. **Figure S5.** Boxplots of PC3&4 loading values arranged by metabolic class. (PPTX 567 kb) Additional file 2:
**Variety, origin, and characteristics of date samples.** (DOCX 60 kb) Additional file 3:
**Sample processing and metabolomics measurement.** (DOCX 31 kb) Additional file 4:
**Metabolomics data from the first sample collection, measured by Metabolon (DS1-bolon).** (XLSX 322 kb) Additional file 5:
**Metabolomics data from the second sample collection, measured by Metabolon (DS2).** (XLSX 379 kb) Additional file 6:
**Abundance profiles of standardized metabolite levels along DS2-mature samples arranged by increasing PC1 scores.** (PDF 1578 kb)

## Abbreviations

VOCs: Volatile organic compounds
DS1-bolon: Metabolomics dataset from the first date collection measured by Metabolon
DS1-sysX: Metabolomics dataset from the first date collection measured by MetaSysX
DS2: Metabolomics data from the second date collection measured by Metabolon
DS2-immature: Metabolomics data from the development stage samples, a subset of the second date collection measured by Metabolon or DS2
DS2-mature: Metabolomics data from the second date collection measured by Metabolon (or DS2) excluding the development stage samples
Batch 1&2 samples: 10 samples from the first sample collection measured again along the samples from the second date collection
