# Supplementary material for: Metabolomics of dates (Phoenix dactylifera) reveals a highly dynamic ripening process accounting for major variation in fruit composition
Source: BMC Plant Biol. 2015 Dec 16;15:291. doi: 10.1186/s12870-015-0672-5 (PMC4681049; doi:10.1186/s12870-015-0672-5)
Supplement: Additional file 1: — Figure S1. Phenotyping the date samples. Figure S2. Iterative optimization of the O2PLS-DA classifier. Figure S3. Quality control based on Metabolon/MetaSysX replicate measurements. Figure S4. Heatmap analysis based on DS1-bolon data. Figure S5. Boxplots of PC3&4 loading values arranged by metabolic class. (PPTX 567 kb) [file 12870_2015_672_MOESM1_ESM.pptx]

## Slide 1
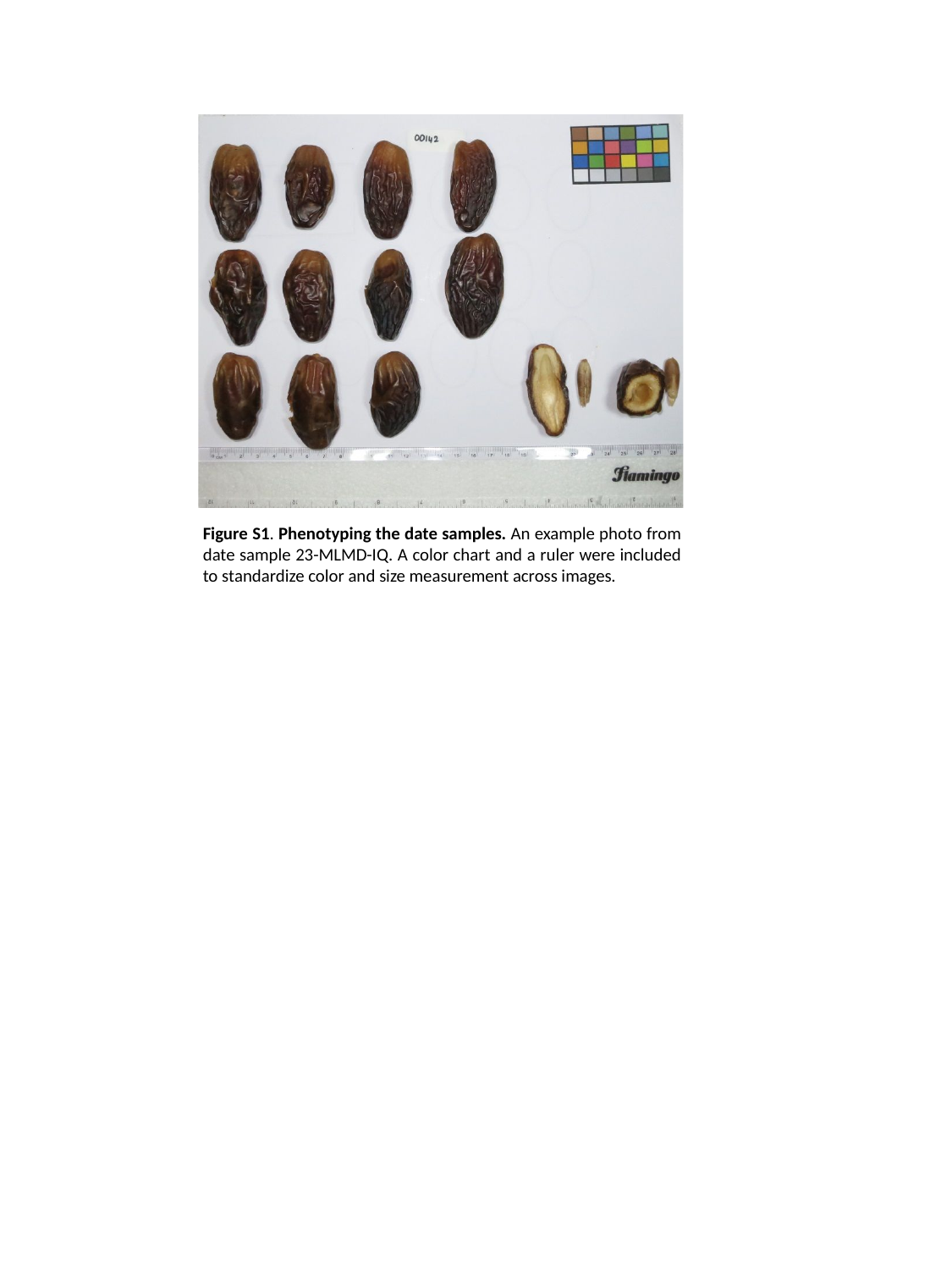

Figure S1. Phenotyping the date samples. An example photo from date sample 23-MLMD-IQ. A color chart and a ruler were included to standardize color and size measurement across images.

## Slide 2
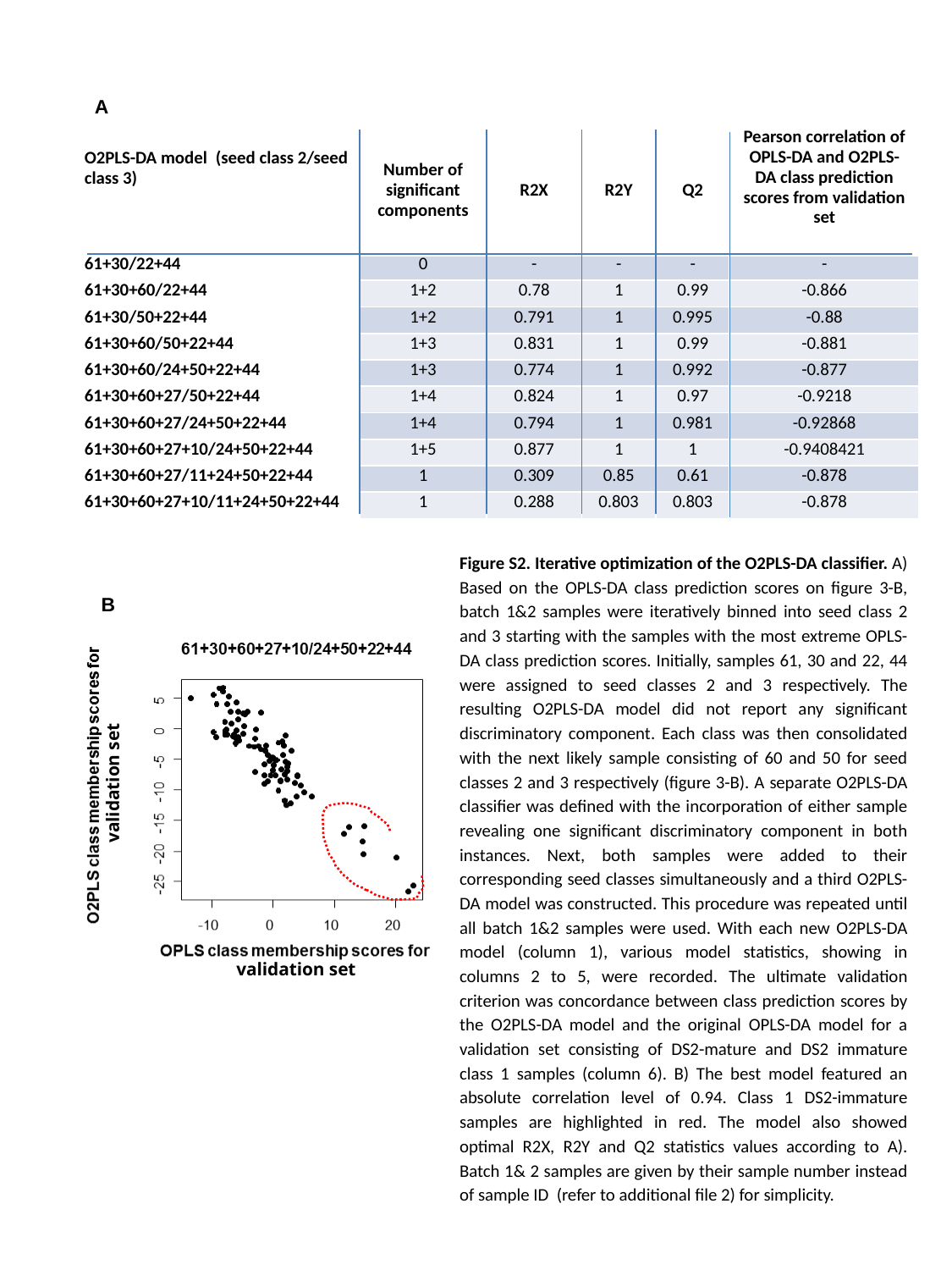

A
| O2PLS-DA model (seed class 2/seed class 3) | Number of significant components | R2X | R2Y | Q2 | Pearson correlation of OPLS-DA and O2PLS-DA class prediction scores from validation set |
| --- | --- | --- | --- | --- | --- |
| 61+30/22+44 | 0 | - | - | - | - |
| 61+30+60/22+44 | 1+2 | 0.78 | 1 | 0.99 | -0.866 |
| 61+30/50+22+44 | 1+2 | 0.791 | 1 | 0.995 | -0.88 |
| 61+30+60/50+22+44 | 1+3 | 0.831 | 1 | 0.99 | -0.881 |
| 61+30+60/24+50+22+44 | 1+3 | 0.774 | 1 | 0.992 | -0.877 |
| 61+30+60+27/50+22+44 | 1+4 | 0.824 | 1 | 0.97 | -0.9218 |
| 61+30+60+27/24+50+22+44 | 1+4 | 0.794 | 1 | 0.981 | -0.92868 |
| 61+30+60+27+10/24+50+22+44 | 1+5 | 0.877 | 1 | 1 | -0.9408421 |
| 61+30+60+27/11+24+50+22+44 | 1 | 0.309 | 0.85 | 0.61 | -0.878 |
| 61+30+60+27+10/11+24+50+22+44 | 1 | 0.288 | 0.803 | 0.803 | -0.878 |
Figure S2. Iterative optimization of the O2PLS-DA classifier. A) Based on the OPLS-DA class prediction scores on figure 3-B, batch 1&2 samples were iteratively binned into seed class 2 and 3 starting with the samples with the most extreme OPLS-DA class prediction scores. Initially, samples 61, 30 and 22, 44 were assigned to seed classes 2 and 3 respectively. The resulting O2PLS-DA model did not report any significant discriminatory component. Each class was then consolidated with the next likely sample consisting of 60 and 50 for seed classes 2 and 3 respectively (figure 3-B). A separate O2PLS-DA classifier was defined with the incorporation of either sample revealing one significant discriminatory component in both instances. Next, both samples were added to their corresponding seed classes simultaneously and a third O2PLS-DA model was constructed. This procedure was repeated until all batch 1&2 samples were used. With each new O2PLS-DA model (column 1), various model statistics, showing in columns 2 to 5, were recorded. The ultimate validation criterion was concordance between class prediction scores by the O2PLS-DA model and the original OPLS-DA model for a validation set consisting of DS2-mature and DS2 immature class 1 samples (column 6). B) The best model featured an absolute correlation level of 0.94. Class 1 DS2-immature samples are highlighted in red. The model also showed optimal R2X, R2Y and Q2 statistics values according to A). Batch 1& 2 samples are given by their sample number instead of sample ID (refer to additional file 2) for simplicity.
B
validation set
validation set

## Slide 3
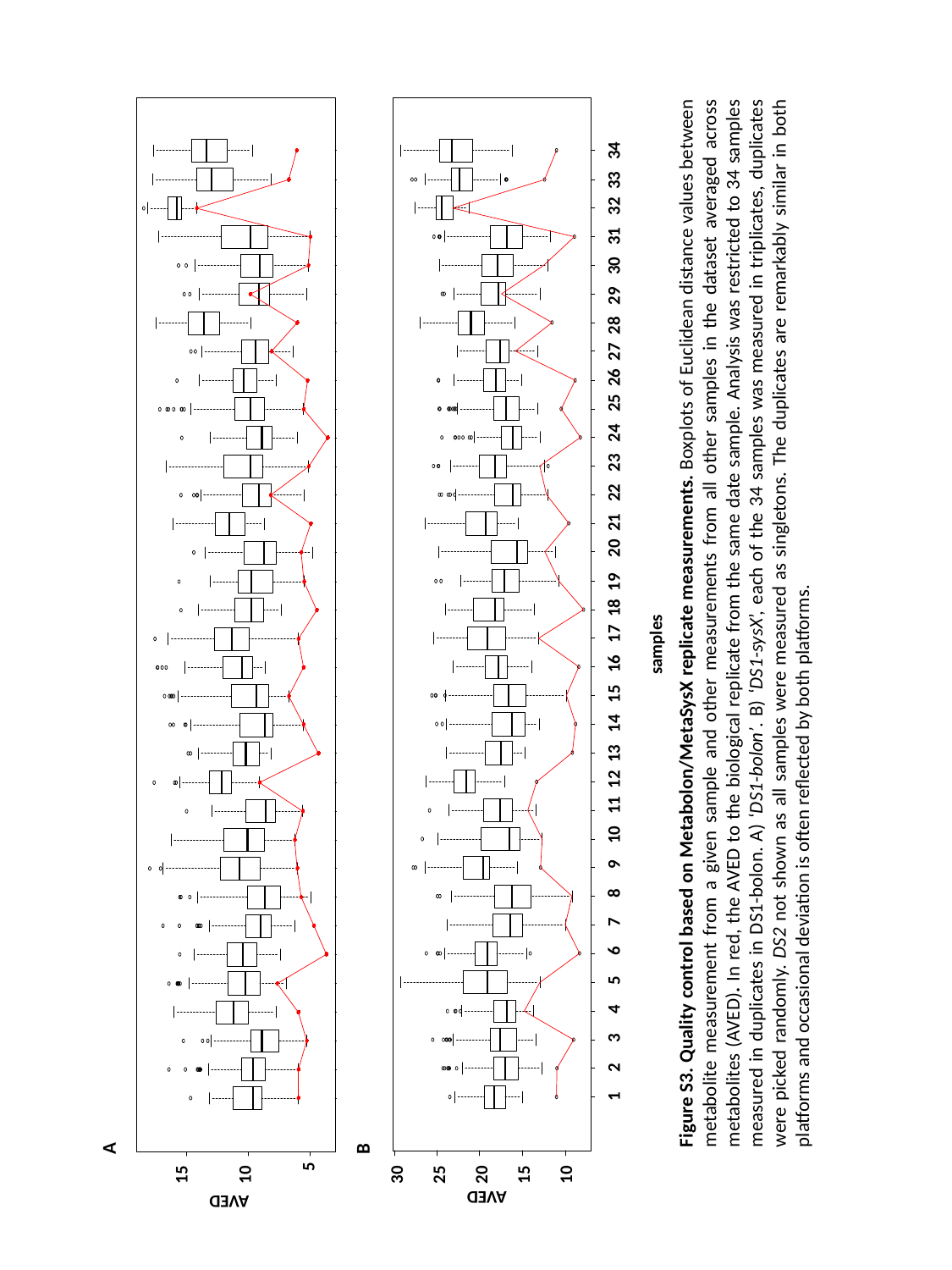

A
15
10
 5
AVED
B
30
25
20
15
10
AVED
 1 2 3 4 5 6 7 8 9 10 11 12 13 14 15 16 17 18 19 20 21 22 23 24 25 26 27 28 29 30 31 32 33 34
 samples
Figure S3. Quality control based on Metabolon/MetaSysX replicate measurements. Boxplots of Euclidean distance values between metabolite measurement from a given sample and other measurements from all other samples in the dataset averaged across metabolites (AVED). In red, the AVED to the biological replicate from the same date sample. Analysis was restricted to 34 samples measured in duplicates in DS1-bolon. A) ‘DS1-bolon’. B) ‘DS1-sysX’, each of the 34 samples was measured in triplicates, duplicates were picked randomly. DS2 not shown as all samples were measured as singletons. The duplicates are remarkably similar in both platforms and occasional deviation is often reflected by both platforms.

## Slide 4
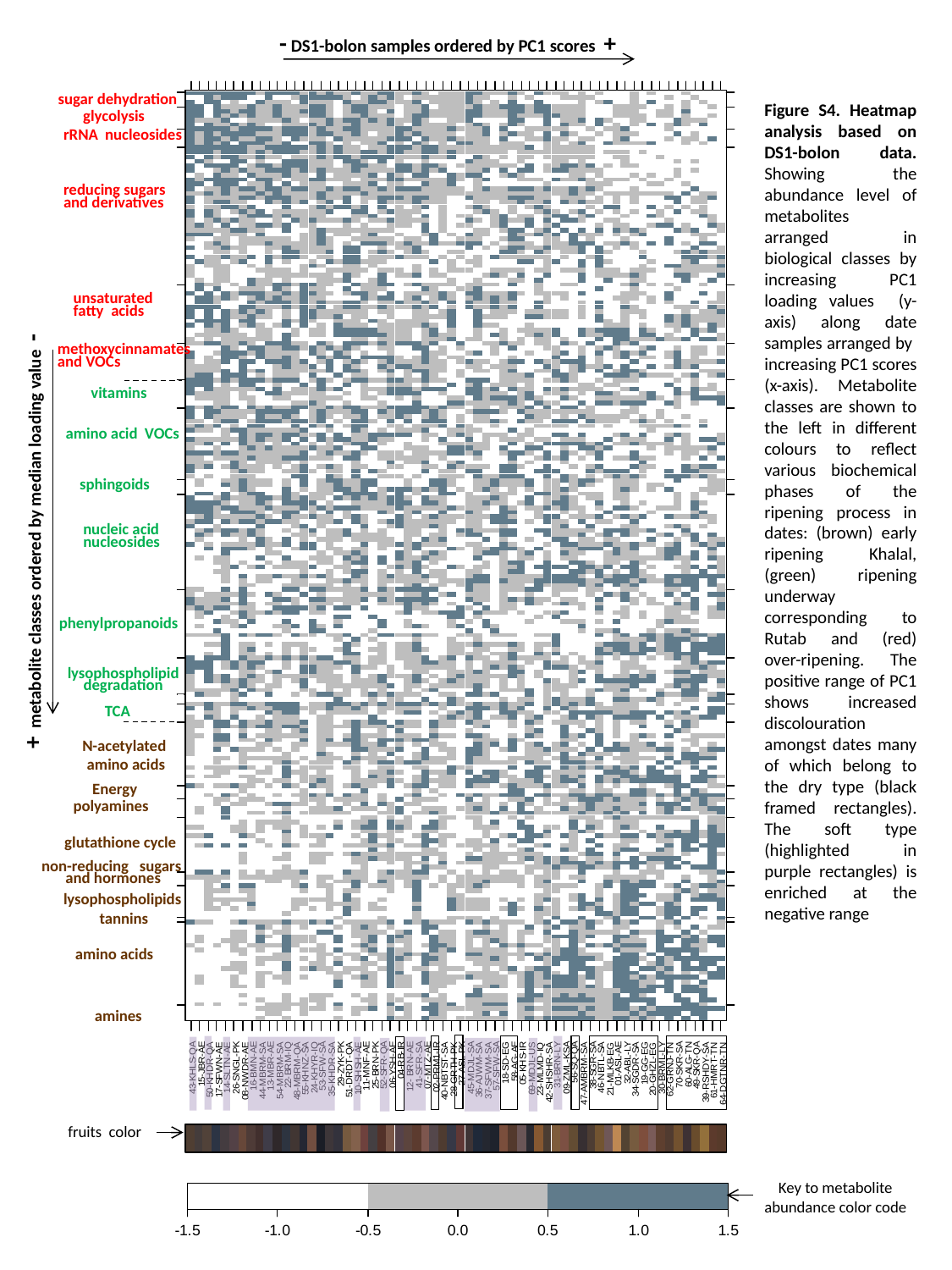

- DS1-bolon samples ordered by PC1 scores +
+ metabolite classes ordered by median loading value -
sugar dehydration
glycolysis
rRNA nucleosides
reducing sugars and derivatives
unsaturated
fatty acids
methoxycinnamates
and VOCs
vitamins
amino acid VOCs
sphingoids
nucleic acid
nucleosides
phenylpropanoids
lysophospholipid degradation
TCA
N-acetylated
 amino acids
Energy
polyamines
glutathione cycle
 non-reducing sugars and hormones
lysophospholipids
tannins
amino acids
amines
Figure S4. Heatmap analysis based on DS1-bolon data. Showing the abundance level of metabolites arranged in biological classes by increasing PC1 loading values (y-axis) along date samples arranged by increasing PC1 scores (x-axis). Metabolite classes are shown to the left in different colours to reflect various biochemical phases of the ripening process in dates: (brown) early ripening Khalal, (green) ripening underway corresponding to Rutab and (red) over-ripening. The positive range of PC1 shows increased discolouration amongst dates many of which belong to the dry type (black framed rectangles). The soft type (highlighted in purple rectangles) is enriched at the negative range
fruits color
Key to metabolite abundance color code

## Slide 5
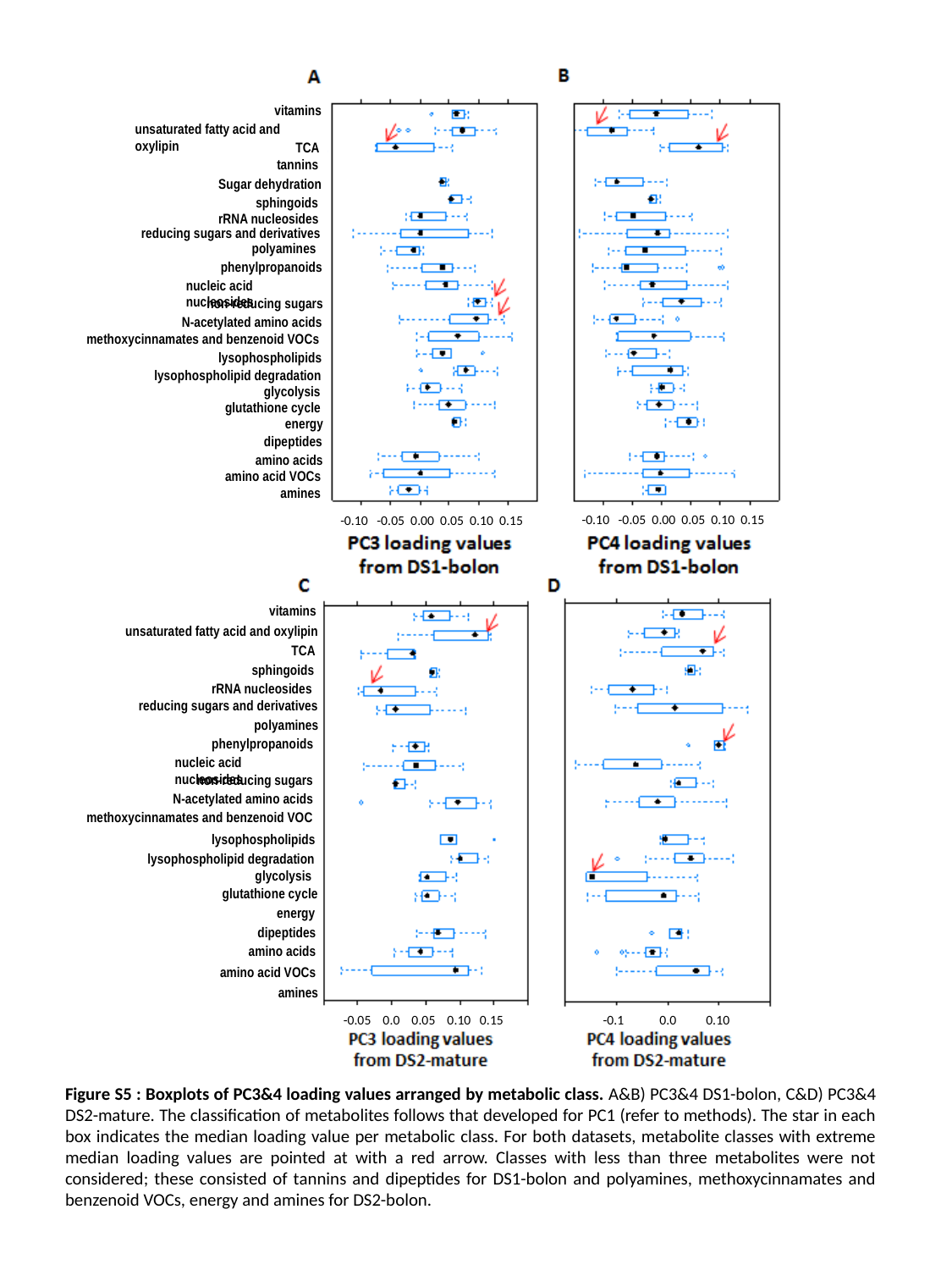

vitamins
unsaturated fatty acid and oxylipin
TCA
tannins
Sugar dehydration
sphingoids
rRNA nucleosides
reducing sugars and derivatives
polyamines
phenylpropanoids
nucleic acid nucleosides
non-reducing sugars
N-acetylated amino acids
methoxycinnamates and benzenoid VOCs
lysophospholipids
lysophospholipid degradation
glycolysis
glutathione cycle
energy
dipeptides
amino acids
amino acid VOCs
amines
-0.10 -0.05 0.00 0.05 0.10 0.15
-0.10 -0.05 0.00 0.05 0.10 0.15
vitamins
unsaturated fatty acid and oxylipin
TCA
sphingoids
rRNA nucleosides
reducing sugars and derivatives
polyamines
phenylpropanoids
nucleic acid nucleosides
non-reducing sugars
N-acetylated amino acids
methoxycinnamates and benzenoid VOC
lysophospholipids
lysophospholipid degradation
glycolysis
glutathione cycle
energy
dipeptides
amino acids
amino acid VOCs
amines
 -0.1 0.0 0.10
 -0.05 0.0 0.05 0.10 0.15
Figure S5 : Boxplots of PC3&4 loading values arranged by metabolic class. A&B) PC3&4 DS1-bolon, C&D) PC3&4 DS2-mature. The classification of metabolites follows that developed for PC1 (refer to methods). The star in each box indicates the median loading value per metabolic class. For both datasets, metabolite classes with extreme median loading values are pointed at with a red arrow. Classes with less than three metabolites were not considered; these consisted of tannins and dipeptides for DS1-bolon and polyamines, methoxycinnamates and benzenoid VOCs, energy and amines for DS2-bolon.
