# Supplementary material for: Metabolomics of dates (Phoenix dactylifera) reveals a highly dynamic ripening process accounting for major variation in fruit composition
Source: BMC Plant Biol. 2015 Dec 16;15:291. doi: 10.1186/s12870-015-0672-5 (PMC4681049; doi:10.1186/s12870-015-0672-5)
Supplement: Additional file 2: — Variety, origin, and characteristics of date samples. (DOCX 60 kb) [file 12870_2015_672_MOESM2_ESM.docx]

**Additional file 2:**  **Variety, origin, and characteristics of date samples.** The sample ID is a combination of the sample number, 2 to 4 letter abbreviation of the variety name and the two letter ISO country code. Other information include the type of variety: *soft, semi-dry* or *dry* as noted in the literature. A fourth class *semi-soft* is common to North African varieties but not Gulf varieties and dates belonging to this class were captured under the *semi-dry* type instead.

| **Sample Number** | **Sample ID** | **Variety Name** | **Country of Production** | **Batch** | **Point of Collection** | **Type** |
| --- | --- | --- | --- | --- | --- | --- |
| 1 | 01-ASL-AE | Aseela | UAE | 1 | festival | dry [[1](#_ENREF_1)] |
| 2 | 02-PRM1-IR | Piarom | Iran | 1 | festival | semi-dry [[2](#_ENREF_2), [3](#_ENREF_3)] |
| 4 | 04-RB-IR | Rabee | Iran | 1 | festival | semi-dry [[4](#_ENREF_4)] |
| 5 | 05-KHS-IR | Khasoy | Iran | 1 | festival |  |
| 6 | 06-YSH-AE | Ayash | UAE | 1 | festival |  |
| 7 | 07-MTZ-AE | Mumtaza | UAE | 1 | festival |  |
| 8 | 08-NWDR-AE | Nawader | UAE | 1 | festival |  |
| 9 | 09-ZML-KSA | Zamlee | KSA | 1 | festival |  |
| 10 | 10-SHSH-AE | Sheshe | UAE | 1&2 | festival | soft [[4](#_ENREF_4), [5](#_ENREF_5)] |
| 11 | 11-MNF-AE | Menefi (Moneifi) | UAE | 1&2 | festival |  |
| 12 | 12- BRN-AE | Berni (barni) | UAE | 1 | festival | soft [[6](#_ENREF_6)] |
| 13 | 13-MBR-AE | Anbara | UAE | 1 | festival | soft [[4](#_ENREF_4)] |
| 14 | 14-SLTN-AE | Sultana | UAE | 1 | festival | soft[[5](#_ENREF_5)] |
| 15 | 15-JBR-AE | Jabri | UAE | 1 | festival |  |
| 16 | 16-BMN-AE | BuMaan | UAE | 1 | festival | soft [[5](#_ENREF_5)] |
| 17 | 17-SFWN-AE | Safawin | UAE | 1 | festival |  |
| 18 | 18-SD-EG | Saeedi | Egypt | 1 | festival | semi-dry [[7](#_ENREF_7)] |
| 19 | 19-GG-EG | Gagee | Egypt | 1 | festival | dry [[7](#_ENREF_7)] |
| 20 | 20-GHZL-EG | Ghazelee | Egypt | 1 | festival | dry [[7](#_ENREF_7)] |
| 21 | 21-MLKB-EG | Malakabi | Egypt | 1 | festival | dry [[7](#_ENREF_7)] |
| 22 | 22-BRM-IQ | Ibrahim ( Iraqi Ajwa) | Iraq | 1&2 | festival | soft [[4](#_ENREF_4), [8](#_ENREF_8)] |
| 23 | 23-MLMD-IQ | Um Alamod | Iraq | 1 | festival |  |
| 24 | 24-KHYR-IQ | Khiyara | Iraq | 1&2 | festival | soft [[4](#_ENREF_4), [8](#_ENREF_8)] |
| 25 | 25-BRN-PK | Barani | Pakistan | 1 | festival |  |
| 26 | 26-SNGL-PK | Sangol | Pakistan | 1 | festival |  |
| 27 | 27-ASL-PK | Aseel | Pakistan | 1&2 | festival | semi-dry [[9](#_ENREF_9)] |
| 28 | 28-GRTH-PK | Gorakh | Pakistan | 1 | festival |  |
| 29 | 29-ZYK-PK | Zayaki | Pakistan | 1 | festival |  |
| 30 | 30-BRML-LY | Burmeel | Libya | 1&2 | festival |  |
| 31 | 31-BRN-LY | Berni | Libya | 1 | festival | soft [[10](#_ENREF_10), [11](#_ENREF_11)] |
| 32 | 32-ABL-LY | Abel | Libya | 1 | festival | semi-dry [[10](#_ENREF_10), [11](#_ENREF_11)] |
| 34 | 34-SGDR-SA | Sagai dry | KSA | 1 | shop | dry [[5](#_ENREF_5)] |
| 35 | 35-KHDR-SA | Khudry | KSA | 1 | shop | soft [[1](#_ENREF_1), [6](#_ENREF_6)] |
| 36 | 36-AJWM-SA | Ajwa Madina | KSA | 1 | shop | soft [[6](#_ENREF_6), [12](#_ENREF_12)] |
| 37 | 37-SFWM-SA | Safawi Madina | KSA | 1 | shop | soft [[1](#_ENREF_1), [12](#_ENREF_12)] |
| 38 | 38-SGR-SA | Sagai rotab | KSA | 1 | shop | dry [[5](#_ENREF_5)] |
| 39 | 39-RSHDY-SA | Rushydia | KSA | 1 | shop | dry [[1](#_ENREF_1)] |
| 40 | 40-NBTST-SA | Naboot Sultan | KSA | 1 | shop |  |
| 41 | 41-SFR-SA | Sufri | KSA | 1 | shop | soft [[6](#_ENREF_6)] |
| 42 | 42-SHSHR-SA | Shieshi rotab | KSA | 1 | shop |  |
| 43 | 43-KHLS-QA | Khlas | Qatar | 1 | shop | soft [[4](#_ENREF_4), [5](#_ENREF_5)] |
| 44 | 44-MBRM-SA | Mabroom | KSA | 1&2 | shop | soft [[6](#_ENREF_6)] |
| 45 | 45-MDJL-SA | Medjoul | KSA | 1 | shop | soft [[4](#_ENREF_4), [13](#_ENREF_13)] |
| 46 | 46-NBTL-SA | Naboot Ali | KSA | 1 | shop | dry [[1](#_ENREF_1)] |
| 47 | 47-AMBRM-SA | Amber Madina | KSA | 1 | shop |  |
| 48 | 48-MBRM-QA | Mabroom | Qatar | 1 | shop | soft [[6](#_ENREF_6)] |
| 49 | 49-SKR-QA | Sukary | Qatar | 1 | shop | semi-dry [[4](#_ENREF_4)] |
| 50 | 50-KHDR-QA | Khudry | Qatar | 1&2 | shop | soft [[1](#_ENREF_1), [6](#_ENREF_6)] |
| 51 | 51-DRDT-QA | Dried dates | Qatar | 1 | shop |  |
| 52 | 52-SFR-QA | Sufri | Qatar | 1 | shop | soft [[6](#_ENREF_6)] |
| 53 | 53-SFW-SA | Safawi | KSA | 1 | shop | soft [[1](#_ENREF_1), [12](#_ENREF_12)] |
| 54 | 54-MBRM-SA | Mabroom | KSA | 1 | shop | soft [[6](#_ENREF_6)] |
| 55 | 55-KHNZ-SA | Kheneizi | KSA | 1 | shop | soft [[5](#_ENREF_5)] |
| 56 | 56-SQ-QA | Suqai | Qatar | 1 | shop | dry [[5](#_ENREF_5)] |
| 57 | 57-SFW-SA | Safawi | KSA | 1 | shop | soft [[1](#_ENREF_1), [12](#_ENREF_12)] |
| 58 | 58-AG-AE | Sagai | UAE | 1 | shop | dry [[5](#_ENREF_5)] |
| 60 | 60-ALG-TN | Allig | Tunisia | 1&2 | shop | semi-dry [[11](#_ENREF_11)] |
| 61 | 61-HMRT-TN | Hamrata | Tunisia | 1&2 | shop | semi-dry [[11](#_ENREF_11)] |
| 62 | 62-GRNJ-TN | Grenja (Tronja) | Tunisia | 1 | shop | semi-dry [[11](#_ENREF_11)] |
| 64 | 64-DGTNR-TN | Deglet Nour | Tunisia | 1 | shop | semi-dry [[13](#_ENREF_13)] |
| 69 | 69-MDJL-US | Medjoul | USA | 1 | shop | soft [[4](#_ENREF_4), [13](#_ENREF_13)] |
| 70 | 70-SKR-SA | Sukkary | KSA | 1 | shop | semi-dry [[4](#_ENREF_4)] |
| 71 | 71-FGGHD-MA | Fegous-ghlid  (Boufegouss) | Morocco | 2 | shop | soft [[11](#_ENREF_11)] |
| 72 | 72-MJLA-MA | Majhool AA | Morocco | 2 | shop | semi-dry [[11](#_ENREF_11)] |
| 73 | 73-JHL-MA | Jihl | Morocco | 2 | farm | dry [[11](#_ENREF_11)] |
| 74 | 74-TDMN-MA | Tadment | Morocco | 2 | farm | semi-dry [[13](#_ENREF_13)] |
| 75 | 75-SYR-MA | Sayer | Morocco | 2 | farm | semi-dry [[11](#_ENREF_11)] |
| 77 | 77-HFS-MA | Hafs | Morocco | 2 | farm | semi-dry [[11](#_ENREF_11)] |
| 78 | 78-BZGZ-MA | Bouzagza | Morocco | 2 | farm |  |
| 79 | 79-BKHD-MA | Boukhanda | Morocco | 2 | farm |  |
| 80 | 80-AGA-MA | Agalid  (Aguelid) | Morocco | 2 | farm | semi-dry [[11](#_ENREF_11)] |
| 81 | 81-LKL-MA | Lakhal | Morocco | 2 | farm |  |
| 82 | 82-NJD-MA | Najda | Morocco | 2 | farm |  |
| 83 | 83-WRDN-MA | Wahardan | Morocco | 2 | farm | semi-dry [[11](#_ENREF_11)] |
| 84 | 84-BDJJ-MA | Bid-djaj | Morocco | 2 | farm |  |
| 85 | 85-AZGHZ-MA | Azaghzaw | Morocco | 2 | farm | semi-dry [[11](#_ENREF_11)] |
| 86 | 86-BSLKH-MA | Bouslikh | Morocco | 2 | farm |  |
| 87 | 87-TZGRT-MA | Tazgaghzt | Morocco | 2 | farm |  |
| 88 | 88-TMRT-MA | Tamaright 2 | Morocco | 2 | farm |  |
| 89 | 89-KLMR-MA | Khalt-lhammar | Morocco | 2 | farm |  |
| 90 | 90-MJL-MA | Mejhool | Morocco | 2 | farm | semi-dry [[11](#_ENREF_11)] |
| 91 | 91-BLZT-MA | Belahzit | Morocco | 2 | farm | semi-dry [[11](#_ENREF_11)] |
| 92 | 92-SHTW-MA | Shetwia | Morocco | 2 | farm | semi-dry [[11](#_ENREF_11)] |
| 93 | 93-BSDN-MA | Booserdoon | Morocco | 2 | farm | dry [[11](#_ENREF_11)] |
| 96 | 96-BKR-MA | Bekri-khder | Morocco | 2 | farm |  |
| 97 | 97-THMT-MA | Tahmoot 2 | Morocco | 2 | farm |  |
| 98 | 98-BFGS-MA | Boufegous rqiq  (Moussa) | Morocco | 2 | farm | soft [[11](#_ENREF_11)] |
| 99 | 99-MJN-MA | Maajoun | Morocco | 2 | farm |  |
| 100 | 100-BSKR-MA | Boussakri | Morocco | 2 | farm | dry [[11](#_ENREF_11)] |
| 101 | 101-ADM-MA | Admoo | Morocco | 2 | farm | dry [[11](#_ENREF_11), [14](#_ENREF_14)] |
| 103 | 103- TZW-MA | Tarzawa | Morocco | 2 | farm |  |
| 104 | 104-BZKR-MA | Boozekri | Morocco | 2 | farm |  |
| 105 | 105-ZGHL-EG | Zaghlool | Egypt | 2 | shop | soft [[11](#_ENREF_11)] |
| 108 | 108-DGBD-DZ | Deglet Bayda | Algeria | 2 | festival | dry [[11](#_ENREF_11), [13](#_ENREF_13)] |
| 109 | 109-DGNH-DZ | Deglet Nour Hourra | Algeria | 2 | festival | dry [[13](#_ENREF_13)] |
| 112 | 112-ADAL-DZ | Adam adaalee | Algeria | 2 | festival | soft [[15](#_ENREF_15)] |
| 113 | 113-TBCHT-DZ | Tantebouchte  (Tantbucht) | Algeria | 2 | festival | soft [[15](#_ENREF_15)] |
| 114 | 114-FGS-DZ | Fegous (Boufegous) | Algeria | 2 | festival | soft [[15](#_ENREF_15)] |
| 117 | 117-DGNR-DZ | Deglet Nour | Algeria | 2 | festival | semi-dry [[11](#_ENREF_11), [13](#_ENREF_13)] |
| 119 | 119-TLMS-DZ | Telemsoua | Algeria | 2 | festival |  |
| 120 | 120-ADBJ-DZ | Adam bujamaa | Algeria | 2 | festival |  |
| 122 | 122-TMJT-DZ | Tamjhourt | Algeria | 2 | festival | soft [[15](#_ENREF_15)] |
| 124 | 124-GHRS-DZ | Gharss | Algeria | 2 | festival | soft [[15](#_ENREF_15)] |
| 125 | 125-DGL-DZ | Ddagla | Algeria | 2 | festival |  |
| 126 | 126-TGBT-DZ | Tagarbushet  (Taqerbucht) | Algeria | 2 | festival | semi-dry [[11](#_ENREF_11)] |
| 128 | 128-DGLT-DZ | Deglet | Algeria | 2 | festival |  |
| 129 | 129-ADMN-DZ | Adam admaan | Algeria | 2 | festival |  |
| 131 | 131-HMR-DZ | Hameera or (H’mira) | Algeria | 2 | festival | soft [[15](#_ENREF_15)] |
| 133 | 133-HRY-DZ | Hamray | Algeria | 2 | festival | soft [[16](#_ENREF_16)] |
| 136 | 136-ZHD-JO | Zehdi | Jordan | 2 | festival |  |
| 137 | 137-LULU-JO | Lulu | Jordan | 2 | festival | soft [[5](#_ENREF_5)] |
| 139 | 139-SIWA-EG | Siwa | Egypt | 2 | festival |  |
| 141 | 141-SEED-LY | Saeedi | Libya | 2 | festival | soft [[11](#_ENREF_11)] |
| 142 | 142-KMSD-SD | Kulma soda | Sudan | 2 | festival |  |
| 143 | 143-GRDL-SD | Grundeela/Qundeil | Sudan | 2 | festival |  |
| 144 | 144-BRKW-SD | Barakawi | Sudan | 2 | festival |  |
| 145 | 145-STBN-SD | Sultana Bedeen | Sudan | 2 | festival |  |
| 146 | 146-ADQM-SA | Aydea Al Qaseem | KSA | 2 | festival |  |
| 147 | 147-KTR-AE | Kattara | UAE | 2 | festival | soft [[4](#_ENREF_4)] |
| 148 | 148-MSL-AE | Msalla | UAE | 2 | festival |  |
| 149 | 149-DBS-AE | Dabbas | UAE | 2 | festival |  |
| 150 | 150-SLG-AE | Sillege | UAE | 2 | festival | soft [[4](#_ENREF_4)] |
| 151 | 151-HMSR-IR | Harmayi Sadrati | Iran | 2 | festival |  |

1. **The description of the dates** [<http://www.manafezinternational.com/dates.html>]

2. Biglari F, AlKarkhi AFM, Easa AM: **Antioxidant activity and phenolic content of various date palm (Phoenix dactylifera) fruits from Iran**. *Food Chem* 2008, **107**(4):1636-1641.

3. Rastegar S, Rahemi M, Baghizadeh A, Gholami M: **Enzyme activity and biochemical changes of three date palm cultivars with different softening pattern during ripening**. *Food Chem* 2012, **134**(3):1279-1286.

4. Al-Khayri JM, Jain SM, Johnson DV: **Date Palm Genetic Resources and utilization**, vol. 2: Asia and Europe: Springer Science + Business Media Dordrecht; 2015.

5. Zahid I, Al Kaabi H: **Date palm research and development programme in the UAE (UAE/2000/ 002)**. In*.*: UAE University & United Nations Office for Project Proposal.

6. **AJWA THE KING OF DATES** [<http://realajwadates.com/>]

7. Rabei.S SWM, Rizk.R.M,EL Sharabasy.S.F: **Morphometric taxonomy of date palm diversity growing in Egypt**. In: *2nd International conference: 29-30 April*: Edited by Egypt.J.Bot. 2012: 175-189.

8. Feraoun AH: **Description of some Iraqi dates varieties**. In*.* Edited by Agriculture; 2002.

9. Markhand GS, Abul-Soad AA, Mirbahar AA, Kanhar NA: **Fruit Characterization of Pakistani Dates**. *Pak J Bot* 2010, **42**(6):3715-3722.

10. Battaglia M: **Libyan Dates - Pomological cards**. In*.*: <http://issuu.com;> 2011.

11. Johnson DV, Al-khayri JM, Jain SM: **Date palm genetic resources and utilization**, vol. 1: Africa and the Americas: Springer Science+Business Media Dordrecht; 2015.

12. [<http://www.madinahdatefactory.com/dates/>]

13. Kader AA, Hussein A: **Harvesting and postharvest handling of dates**: International Center for Agricultural Research in the Dry Areas (ICARDA); 2009.

14. Sedra MH, Lashermes P, Trouslot P, Combes MC, Hamon S: **Identification and genetic diversity analysis of date palm (Phoenix dactylifera L.) varieties from Morocco using RAPD markers**. *Euphytica* 1998, **103**(1):75-82.

15. Acourene S, Djafri K, Benchabane A, Tama M, Taleb B: **Dates quality and assessment of the main date palm cultivars grown in Algeria**. *Annual Research & Review in Bilogy* 2014, **4**(3):12.

16. Absi R: **Analyse de la diversite varietale du Palmier Dattier (Phoenix Dactylifera L.): Cas des Ziban (Region de Sidi Okba)**. Universite Mohamed Khider Biskra; 2013.
