# Supplementary material for: Metabolomics of dates (Phoenix dactylifera) reveals a highly dynamic ripening process accounting for major variation in fruit composition
Source: BMC Plant Biol. 2015 Dec 16;15:291. doi: 10.1186/s12870-015-0672-5 (PMC4681049; doi:10.1186/s12870-015-0672-5)
Supplement: Additional file 3: — Sample processing and metabolomics measurement. (DOCX 31 kb) [file 12870_2015_672_MOESM3_ESM.docx]

**Additional file 3: Sample processing and metabolomics measurement**

**MetaSysX platform**

***Sample preprocessing*:** 50 mg of the peel and flesh of the date fruits were flash frozen in liquid nitrogen and processed according to standardized procedures developed by MetaSysX GmbH. ([Giavalisco, Li et al. 2011](#_ENREF_26)). Briefly, the plant tissue (peel and flesh) was homogenized in a 2 ml Eppendorf tube (Eppendorf, Germany) twice for a minute at maximum speed within a Retsch mill (Retsch, Germany). For metabolite extraction, a mixed solvent of methanol:methyl-tert-butyl-ether:water (1:3:1) was added to each homogenate and samples were shaken for 30 min at 4^0^ C and further incubated for 10 minutes in an ice cooled ultra-sonication bath. Upon incubation, 650 µL of UPLC-grade methanol: water (3:1) was added to the samples, followed by vortexing and centrifugation for 5 minutes at 4^0^ C in a tabletop centrifuge (Eppendorf, Germany). The above procedures led to a phase separation, providing the upper organic phase containing hydrophobic metabolites (lipids) and a lower aqueous phase containing polar and semi-polar metabolites. The phases were separated, dried in a speedvac concentrator (Centrivac, Heraeus, Germany) and stored at -80^0^ C for further analyses.

***Metabolite measurements with Ultra Performance Liquid Chromatography FT Mass Spectroscopy (UPLC –FT-MS):*** The dried metabolite extracts from aqueous phase were re-suspended in 100 µL of UPLC grade water and 2 µL was injected on a C_18_ reversed phase column (100 mm*2.1mm *1.81 µm particles, Waters), using a Waters Acquity Ultra Performance Liquid Chromatography (UPLC) system. The two mobile phases were 0.1% formic acid in water (Buffer A) and 0.1 % formic acid in acetonitrile (Buffer B). The gradient separation occurred at a flow rate of 400 µL/min in 20 minutes of total run time as previously described in ([Giavalisco, Li et al. 2011](#_ENREF_26)). The dried metabolite extracts from the organic phase were re-suspended in 500 µL of UPLC grade acetonitrile: isopropanol (7:3) and 2 µL was injected on a C_8_ reversed phase column (100 mm*2.1mm *1.71 µm particles, Waters). The mobile phases were water (UPLC MS grade, BioSolve) with 1% 1 M NH_4_ Ac, 0.1% acetic acid (Buffer A) and acetonitrile: isopropanol (7:3, UPLC grade, BioSolve) also containing 1% 1 M NH_4_Ac, 0.1 % acetic acid (Buffer B). The gradient separation followed at a flow rate of 400 µL/min in 20 minutes of total run time as previously described in ([Giavalisco, Li et al. 2011](#_ENREF_26)). The mass spectra were obtained using an orbitrap Exactive mass spectrometer (Thermo Fischer, Germany). The spectrum was recorded alternating between full-scan and all ion-fragmentation scan modes covering a mass range from 150- 1500 m/z. The resolution was set to 10,000 with 10 scans per second restricting Orbitrap loading time to 100 ms. With the capillary voltage set to 3KV and capillary temperature set to 150^0^ C and other parameters specified in ([Giavalisco, Li et al. 2011](#_ENREF_26)), the spectra was recorded from 1 minute to 17 minutes of the UPLC gradients.

***Metabolite measurements with Gas Chromatography - Time-of-Flight Mass Spectrometer (GC-TOFMS)***: Samples were measured using the Agilent Technologies GC (Agilent, USA) coupled to a Leco Pegasus HT mass spectrometer (Leco Corporation, USA). The spectrometer is empowered with an Electron Impact ionization source (EI) and a Time of Flight (TOF) mass analyzer. For the GCMS-MS, the column height is 30 meters with the starting temperature 85° C for 2 minutes and the gradient is 15C per minute up to 36/3600C.

***Signal processing and metabolite calling:*** Chromatograms from the UPLC-FT-MS runs were analyzed and processed using the software REFINER MS^®^ 7.5 (Genedata, Switzerland). The data was further filtered and analyzed using in-house software tools. The alignment of the chromatograms was performed by selecting a feature, present in all three replicates of a date fruit variety and at least in one of the date fruits. Each feature had an average retention time (RI) and an average m/z value. The alignment was performed for lipids, polar and semi-polar metabolites. The dataset was refined by removing isotopic peaks, in-source fragments of analytes from ionization and lower intense adduct of the same analyte as in ([Giavalisco, Li et al. 2011](#_ENREF_26), [Hummel, Segu et al. 2011](#_ENREF_31)). The compounds were annotated using the MetaSysX GmbH’s pre-established library of reference compounds in terms of mass and retention time. The GC-MS data files extracted from the Leco Pegasus software was imported into the statistical package R. Further, a software package, the TargetSearch ([Cuadros-Inostroza, Caldana et al. 2009](#_ENREF_13)) transformed the retention time (RT) to retention index (RI) to generate peaks and align chromatograms. The spectra and the RI were compared to the Golm Metabolome Database (GMD) ([Kopka, Schauer et al. 2005](#_ENREF_36)). The metabolites with a RT and a mass spectrum that did not match with the GMD were termed ‘unknown metabolites’. Obtained data from both platforms was normalized according to sample weight and to the measurement day to minimize process error over the course of many days of measurement.

**Metabolon platform**

***Sample preprocessing:*** With batch 1, material from MetaSysX pre-processed samples was used. With batch 2, the following procedure was followed: The samples were weighed and frozen at – 80^0^ C prior to extraction. The samples were prepared and extracted according to the standard solvent extraction method by Metabolon Inc. ([Evans, DeHaven et al. 2009](#_ENREF_22)). In brief, beads were added to the pre-weighed frozen samples together with water (8 µL of per mg of sample) for homogenisation. The blanks (aliquot of pure water) were prepared by adding 700 µL of water to three cryovials. The blanks and the samples were continuously stirred on the GenoGrinder (Glen Mills GenoGrinder 2000, Germany) at 1000 strokes per minute for five minutes, to ensure complete homogenization. 100 µL of aliquot from each sample was transferred to the plates. For quality control (QC) purposes, additional samples were added including: six of 100 µL blanks; six of 100 µL of homogenous mixture of pooled samples, prepared by pooling 30 µL of each sample; one of 100 µL of human plasma, on each plate. All samples were further processed as follows. The samples were loaded on three plates. To each sample, 450 µL of extraction solvent (MeOH with 10 µL /ml chlorophenylalanine, 2.5 µL /ml 2-fluorophenylglycine, 25 µg/ml d-6 cholestrol and 25 µL /ml tridecanoic acid) was added. The samples were then shaken on the GenoGrinder (GenoGrinder, Spex, USA) at 675 strokes per min for two minutes and centrifuged at 2000 rpm for 5 minutes on a Beckman centrifuge (Beckman GS-6R Centrifuge, USA) at 4^0^C. The extracted samples were divided into equal parts for metabolomics analysis on the Gas Chromatography Mass Spectrometry (GC/MS) and the Orbitrap Elite accurate Liquid Chromatography Mass Spectrometry^2^ (LC-MS-MS) platforms. Four sets of samples were prepared by the Hamilton robot (Hamilton Star, Germany) by transferring 110 µL aliquots from each well to three PCR plates, each for LC positive, LC negative, replicate set and one to 250 µL auto sampler vial inserts for GC. All samples were dried for 120 minutes by using a Zymark Turbovap 96 (Zymark Turbovap, USA) followed by overnight incubation in a drybox to ensure optimal dryness of the sample.

***Metabolite measurements with Ultrahigh Performance Liquid Chromatography/Mass Spectroscopy (UPLC/MS/MS):***  The UPLC/MS/MS analysis was based on the Waters ACUITY ultra performance liquid chromatography (Waters Corporation, USA) and the ThermoFischer Scientific Orbitrap Elite high-resolution accurate mass spectrometer (Thermo Fischer Scientific Inc., USA) equipped with a heated electrospray ionization (HESI) source and an Orbitrap mass analyzer. The dried sample extracts for the LC positive and LC negative mode were reconstituted in acidic and basic LC- compatible solvents. Two independent injections were performed on each sample using separate dedicated columns for optimized acidic positive ions and the other for optimized basic negative ions. The acidic samples were reconstituted by gradient elution of water and methanol containing 0.1 % formic acid whereas; the basic samples were reconstituted by gradient elution of water and methanol containing 6.5mM ammonium bicarbonate ([Evans, DeHaven et al. 2009](#_ENREF_22)). The mass spectra analysis alternated between MS and data dependent MS^2^ scans using dynamic exclusion.

***Metabolite measurements with GC/MS***: The samples assigned for the GC/MS analysis were further dried under vacuum desiccation for an entire day and derivatized under dried nitrogen using bistrimethyl-silyl-trifluoroacetamide (BSTFA). The GS/MS analysis was based on a Thermo Finnigan^TM^ TRACE^TM^ DSQ^TM^ (ThermoFinnigan, USA) fast-scanning single –quadrupole mass spectrophotometer using electron impact ionization source. The GC column was 5% phenyl and the temperature ramp range was from 40 to 300^0^ C in a time span of 16 minutes.

***Signal processing and metabolite calling:*** The raw data files from both platforms were extracted using the in-house informatics system. The system possesses an automated laboratory information management system (LIMS), data extraction and peak identification software, data processing tools for QC and compound identification and information interpretation and visualization tools. The date extraction and compound identification was performed using Metabolon’s web-service based hardware and software platform. A reference library maintained by Metabolon Inc. ([DeHaven, Evans et al. 2010](#_ENREF_15)), consists of chemical standards with retention time, retention index, mass to charge ratio (m/z) and chromatographic data including MS/MS spectral data was used to identify metabolites in experimental samples. The compounds were identified by the comparison of obtained data to the library entries based on three types of criteria; retention index (RI) within a narrow RI window, accurate mass match to the library (+/- 0.005 atomic mass units) and MS/MS forward and reverse scores between experimental data and standards in the library ([DeHaven 2012](#_ENREF_14)). In this study, the samples were analyzed over a span of two or three days for batches 1 and 2, respectively, and therefore data normalization step was performed to correct variation from instrument inter-day tuning differences.
