# Supplementary material for: Metabolomics of dates (Phoenix dactylifera) reveals a highly dynamic ripening process accounting for major variation in fruit composition
Source: BMC Plant Biol. 2015 Dec 16;15:291. doi: 10.1186/s12870-015-0672-5 (PMC4681049; doi:10.1186/s12870-015-0672-5)
Supplement: Additional file 6: — Abundance profiles of standardized metabolite levels along DS2-mature samples arranged by increasing PC1 scores. (PDF 1578 kb) [file 12870_2015_672_MOESM6_ESM.pdf]

Additional file 4. Abundance profiles of standardized metabolite levels along DS2-mature samples arranged by increasing PC1 scores. Shown within their respective classes

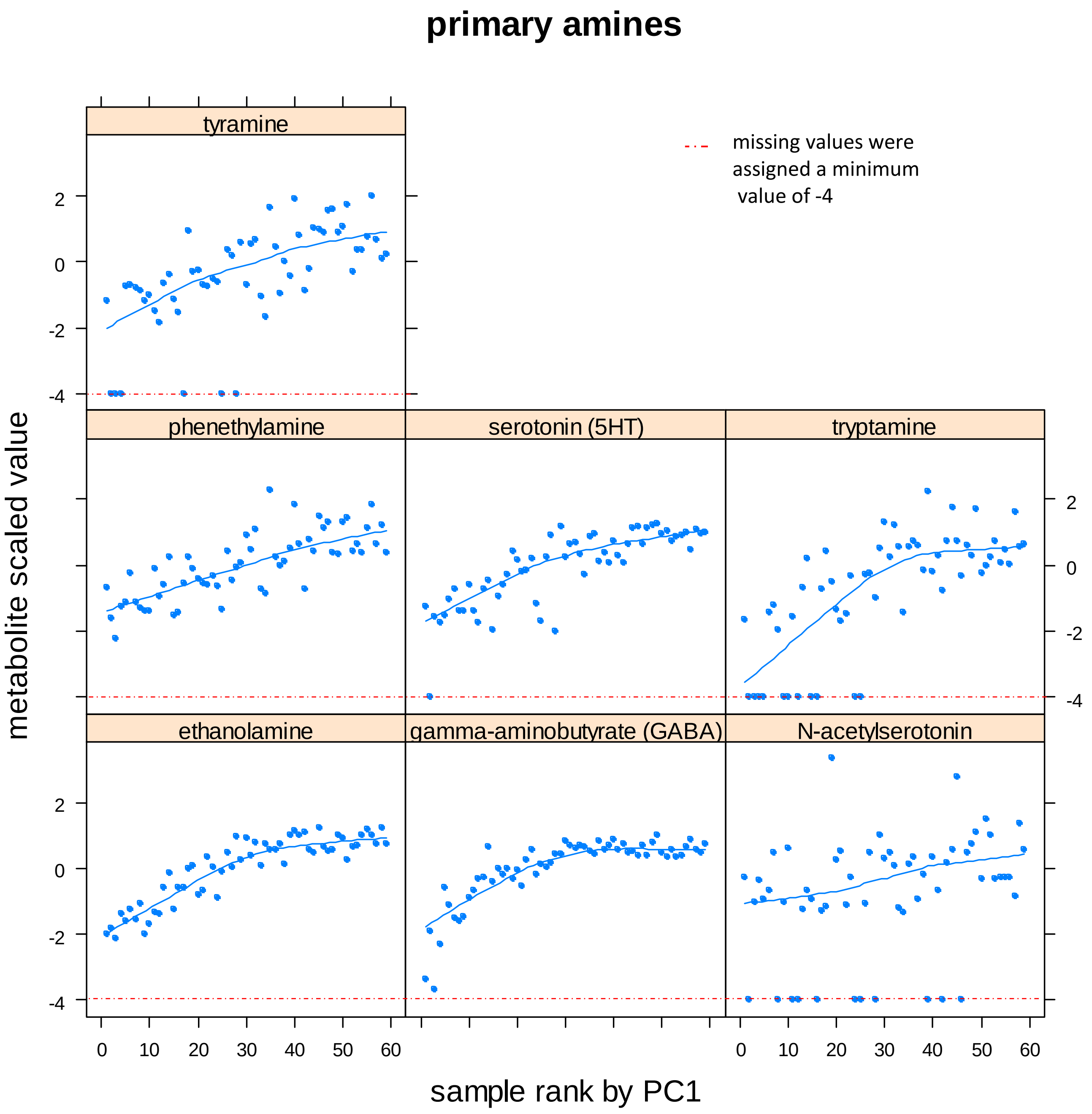

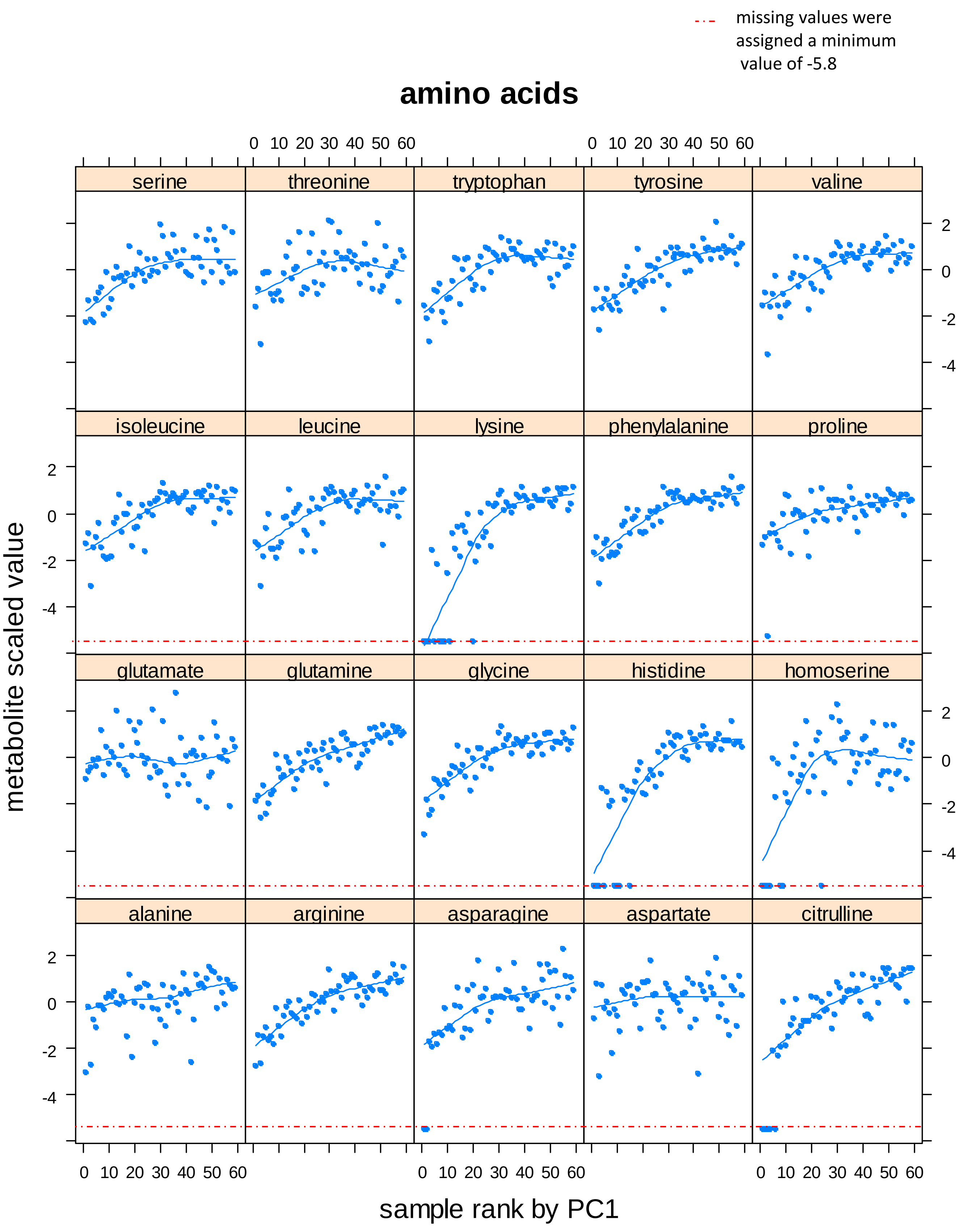

missing values were assigned a minimum value of -5.8
amino acids
serine
threonine
tryptophan
tyrosine
valine
isoleucine
leucine
lysine
phenylalanine
proline
glutamate
glutamine
glycine
histidine
homoserine
alanine
arginine
asparagine
aspartate
citrulline
metabolite scaled value
sample rank by PC1
0 10 20 30 40 50 60
2 0 -2 -4

glutathione cycle and glutathione metabolism

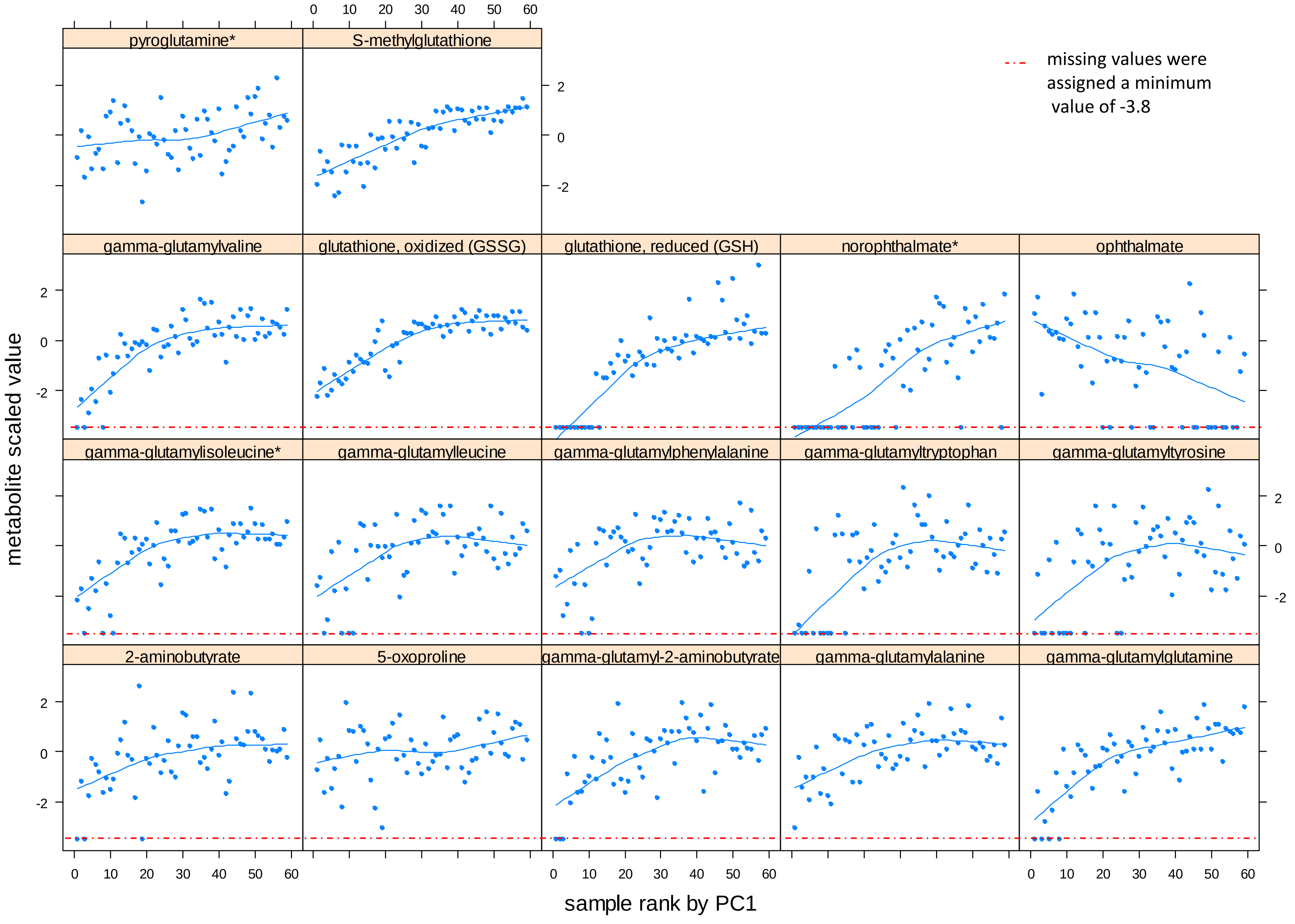

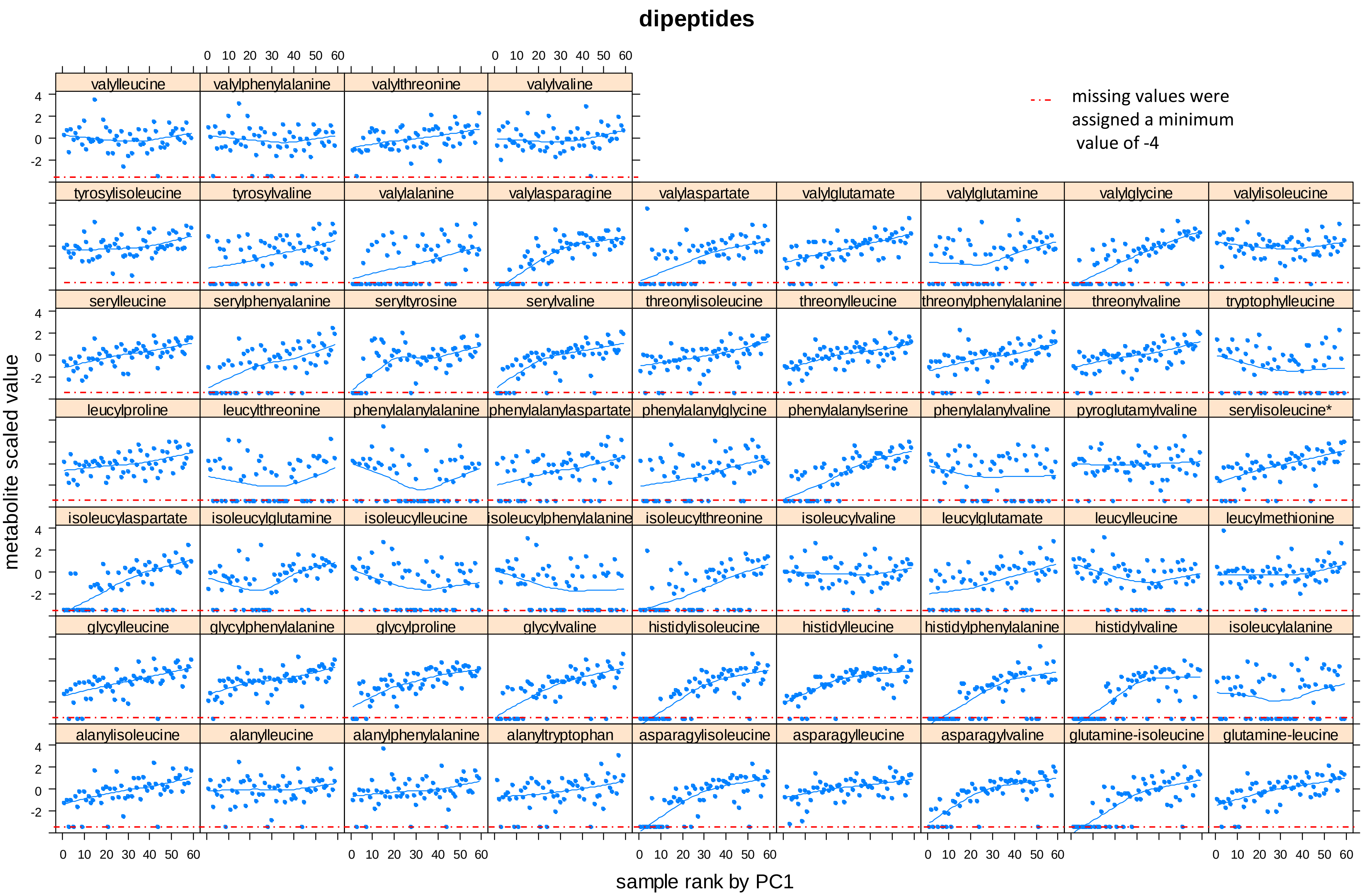

dipeptides
missing values were assigned a minimum value of -4
valylleucine
valylphenylalanine
valylthreonine
valylvaline
tyrosylisoleucine
tyrosylvaline
valylalanine
valylasparagine
valylaspartate
valylglutamate
valylglutamine
valylglycine
valylisoleucine
serylleucine
serylphenylalanine
seryltyrosine
serylvaline
threonylisoleucine
threonylleucine
threonylphenylalanine
threonylvaline
tryptophylleucine
leucylproline
leucylthreonine
phenylalanylalanine
phenylalanylaspartate
phenylalanylglycine
phenylalanylserine
phenylalanylvaline
pyroglutamylvaline
serylisoleucine*
isoleucylaspartate
isoleucylglutamine
isoleucylleucine
isoleucylphenylalanine
isoleucylthreonine
isoleucylvaline
leucylglutamate
leucylleucine
leucylmethionine
glycylleucine
glycylphenylalanine
glycylproline
glycylvaline
histidylisoleucine
histidylleucine
histidylphenylalanine
histidylvaline
isoleucylalanine
alanylisoleucine
alanylleucine
alanylphenylalanine
alanyltryptophan
asparagylisoleucine
asparagylleucine
asparagylvaline
glutamine-isoleucine
glutamine-leucine
metabolite scaled value
sample rank by PC1

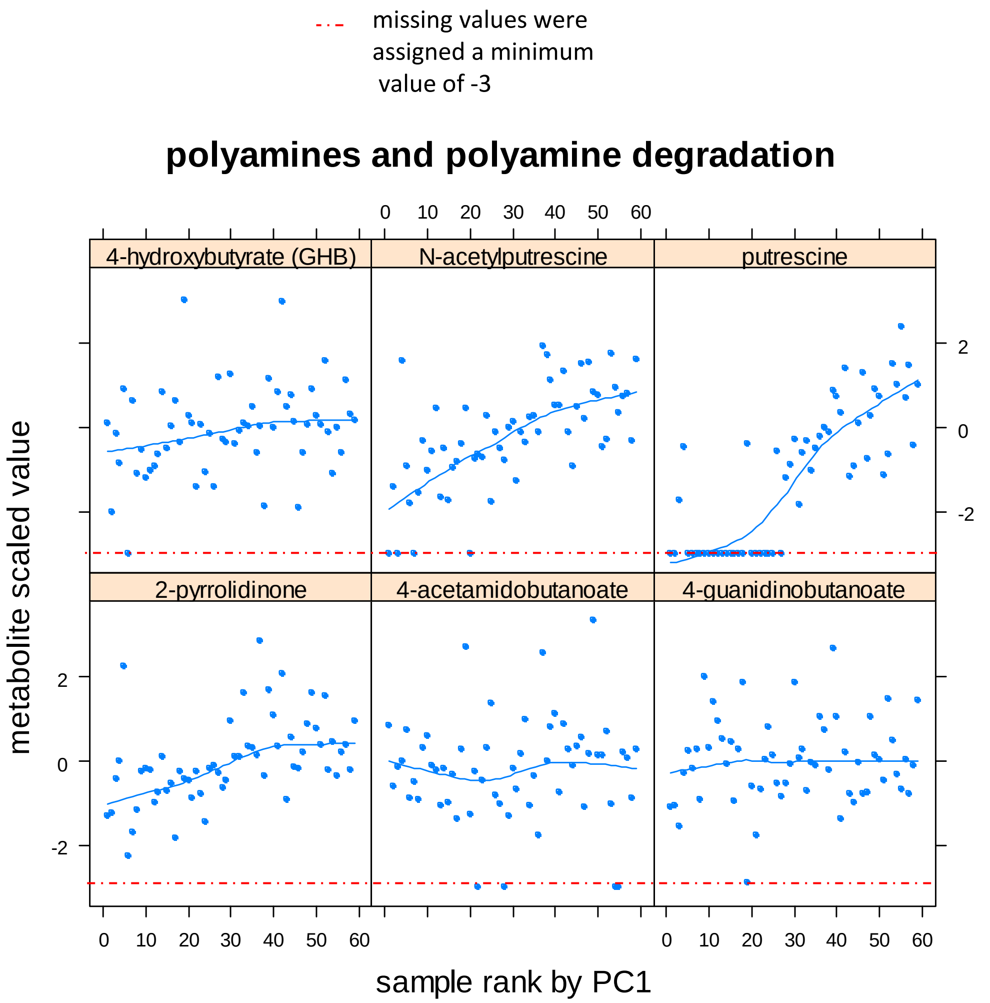
missing values were assigned a minimum value of -3
polyamines and polyamine degradation
4-hydroxybutyrate (GHB)
N-acetylputrescine
putrescine
2-pyrrolidinone
4-acetamidobutanoate
4-guanidinobutanoate
metabolite scaled value
sample rank by PC1
0 10 20 30 40 50 60
2 0 -2

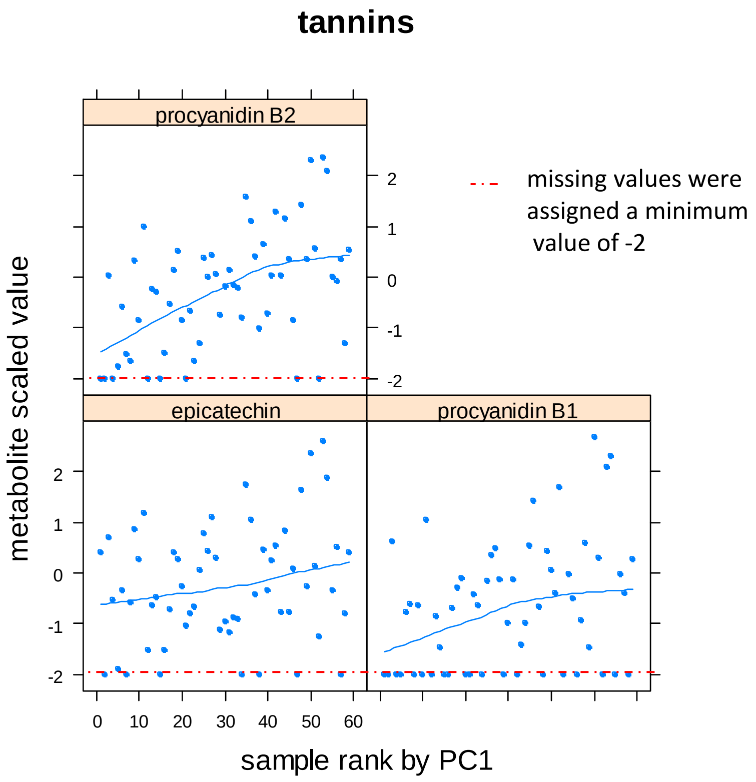
tannins
procyanidin B2
epicatechin
procyanidin B1
missing values were assigned a minimum value of -2
metabolite scaled value
sample rank by PC1
0 10 20 30 40 50 60
2 1 0 -1 -2

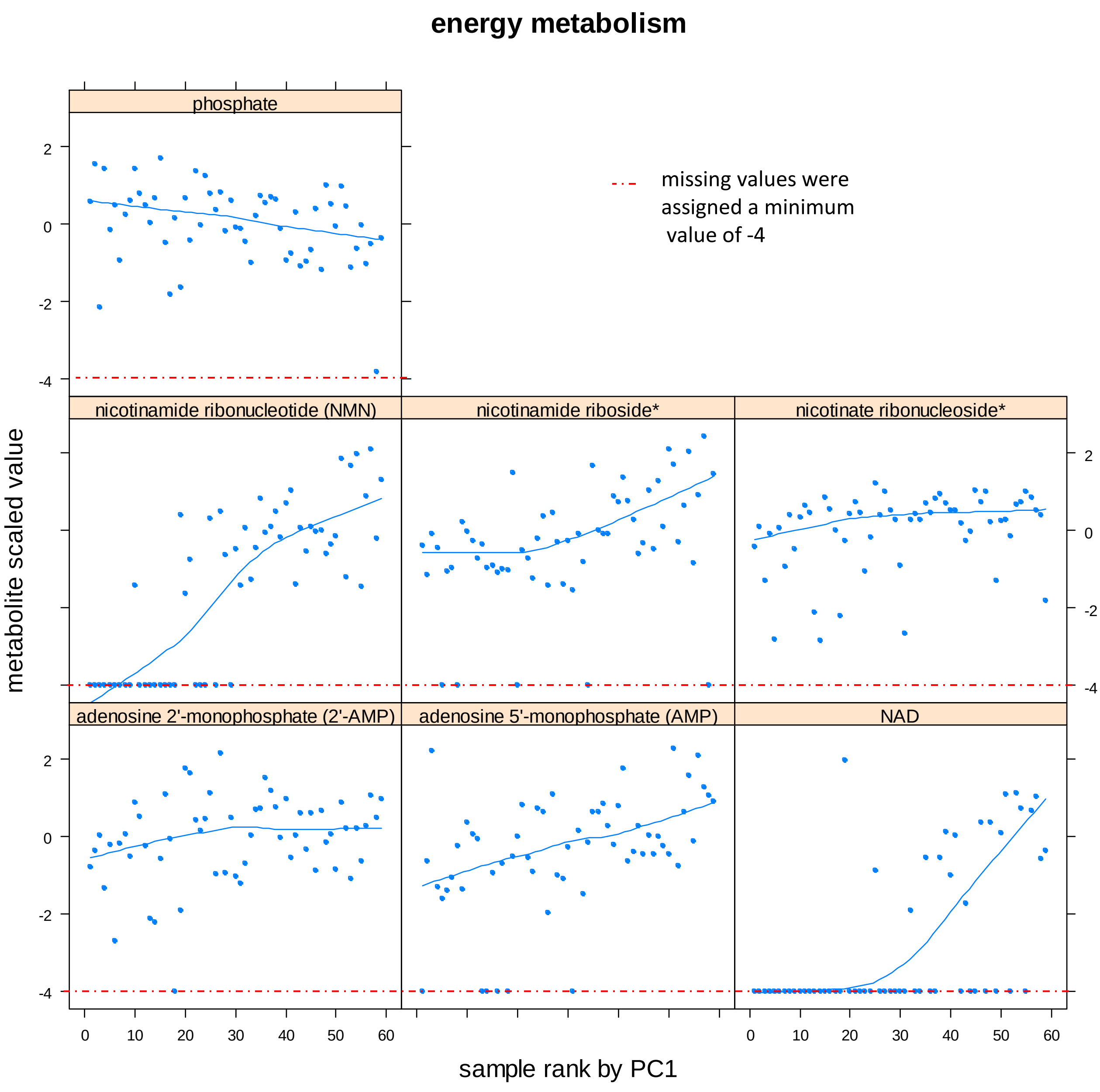
energy metabolism
phosphate
missing values were assigned a minimum value of -4
nicotinamide ribonucleotide (NMN)
nicotinamide riboside*
nicotinate ribonucleoside*
adenosine 2'-monophosphate (2'-AMP)
adenosine 5'-monophosphate (AMP)
NAD
metabolite scaled value
sample rank by PC1

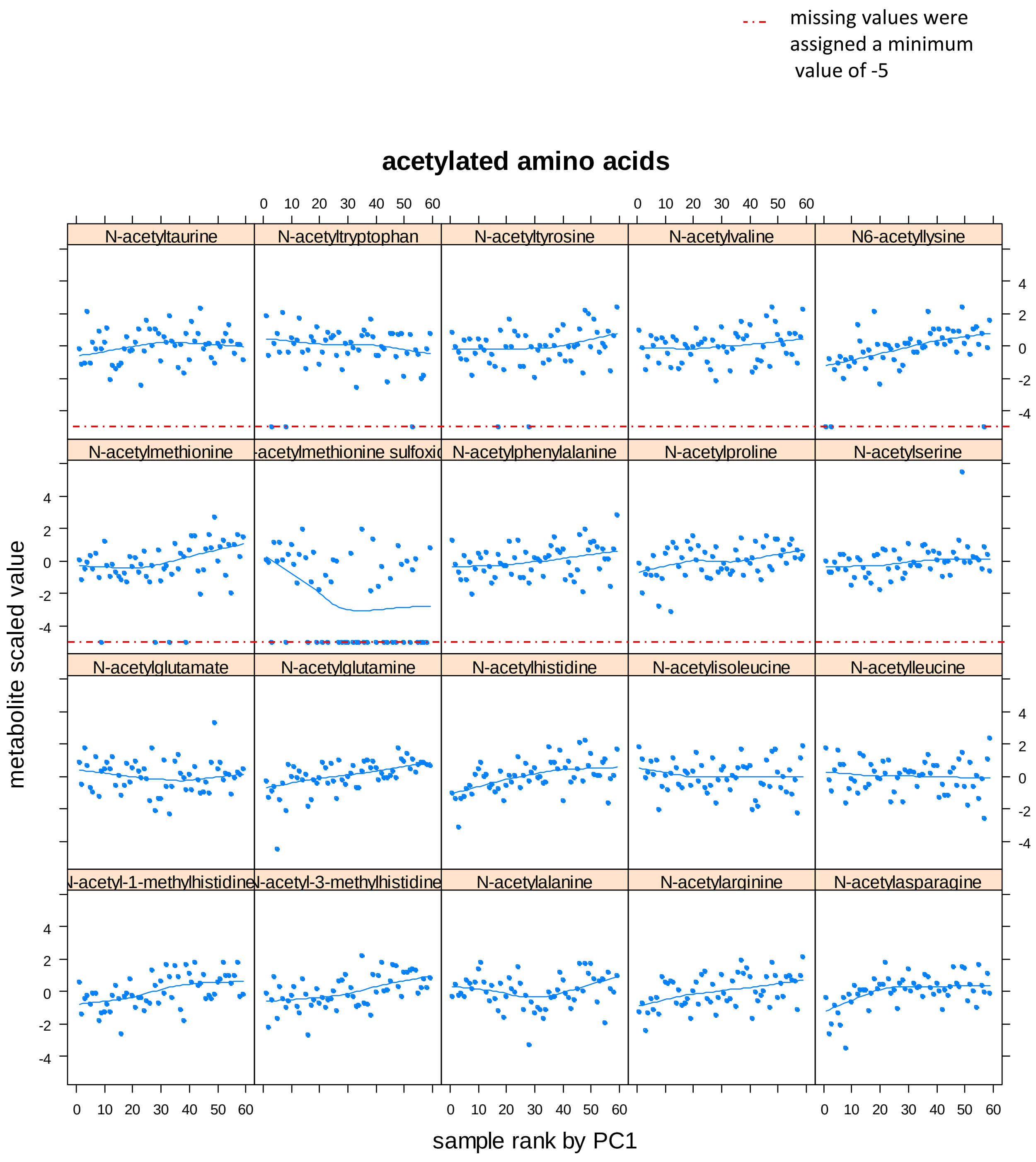

missing values were assigned a minimum value of -5
acetylated amino acids
N-acetyltaurine
N-acetyltryptophan
N-acetyltyrosine
N-acetylvaline
N6-acetyllysine
N-acetylmethionine
-acetylmethionine sulfoxi
N-acetylphenylalanine
N-acetylproline
N-acetylserine
N-acetylglutamate
N-acetylglutamine
N-acetylhistidine
N-acetylisoleucine
N-acetylleucine
-acetyl-1-methylhistidine
-acetyl-3-methylhistidine
N-acetylalanine
N-acetylarginine
N-acetylasparagine
metabolite scaled value
sample rank by PC1

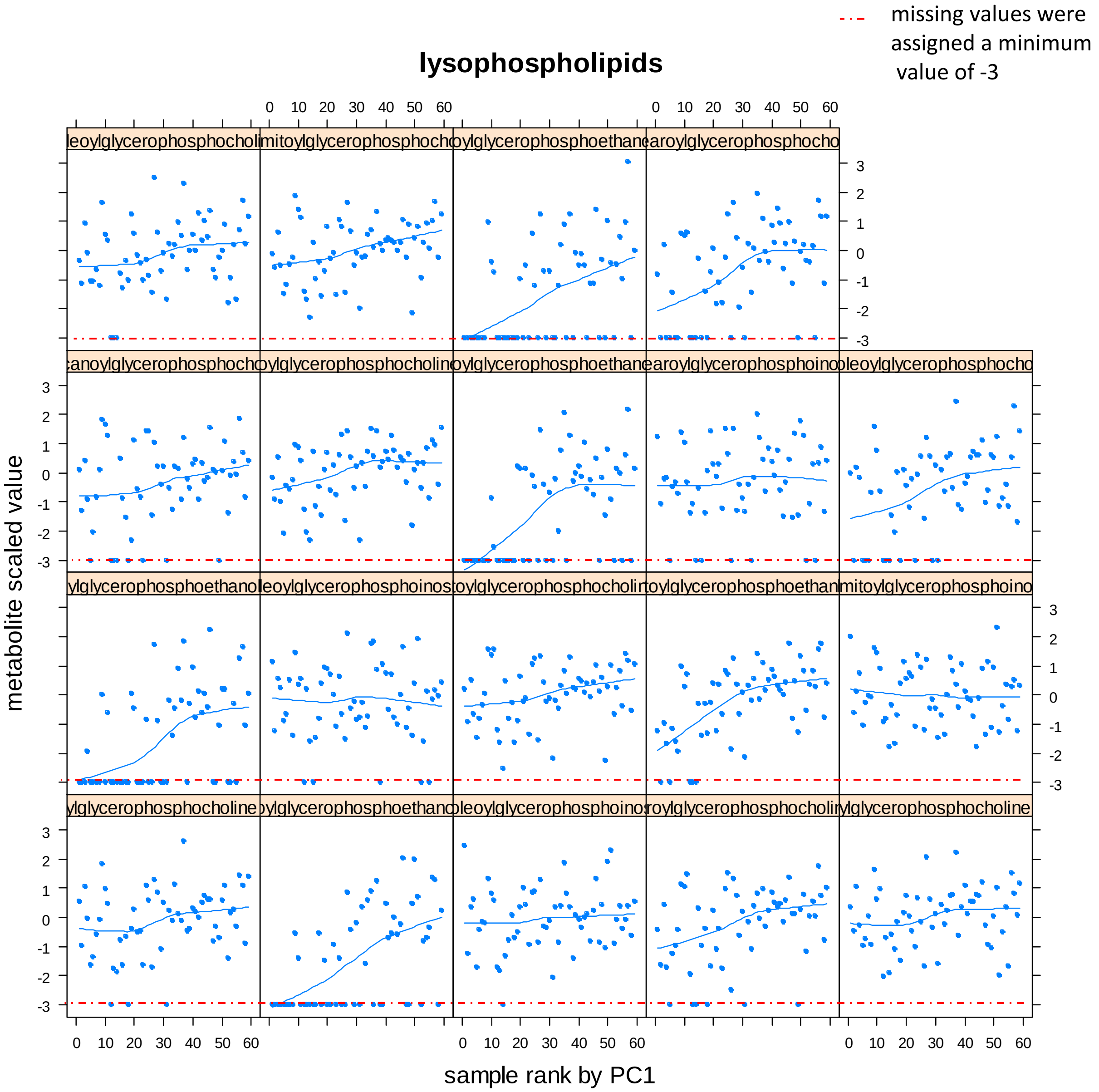

missing values were assigned a minimum value of -3
lysophospholipids
metabolite scaled value
sample rank by PC1

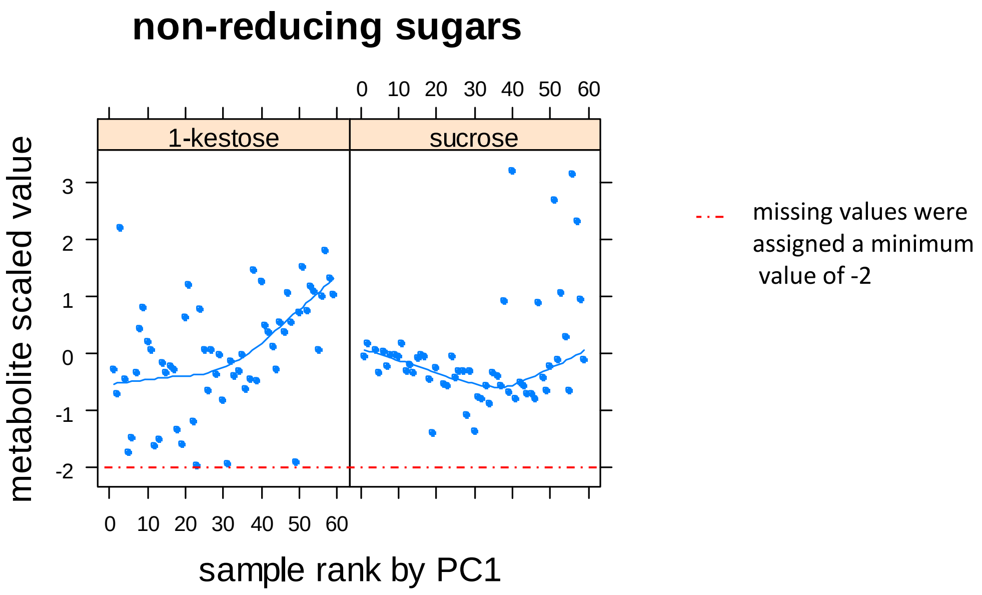

non-reducing sugars
1-kestose
sucrose
metabolite scaled value
sample rank by PC1
missing values were assigned a minimum value of -2

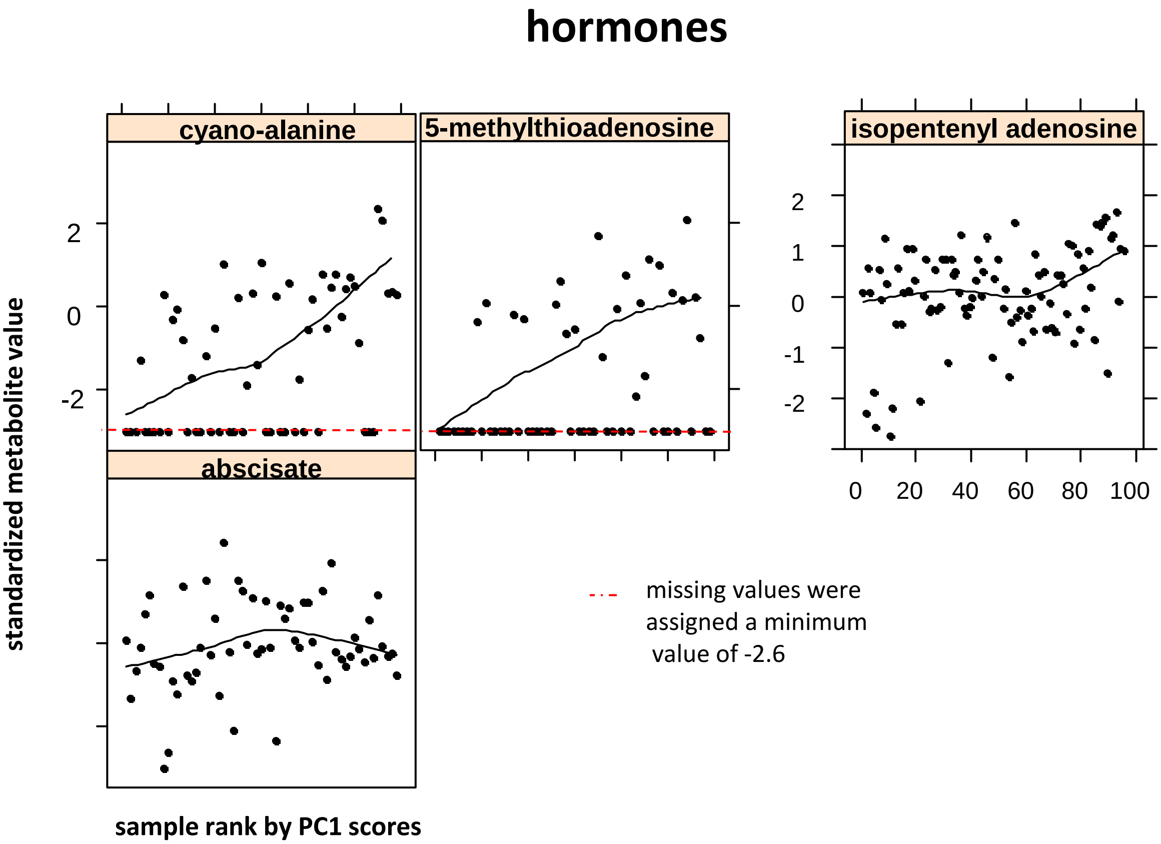
hormones
cyano-alanine
5-methylthioadenosine
isopentenyl adenosine
abscisate
2
0
-2
1
-1
0
20
40
60
80
100
standardized metabolite value
sample rank by PC1 scores
missing values were assigned a minimum value of -2.6

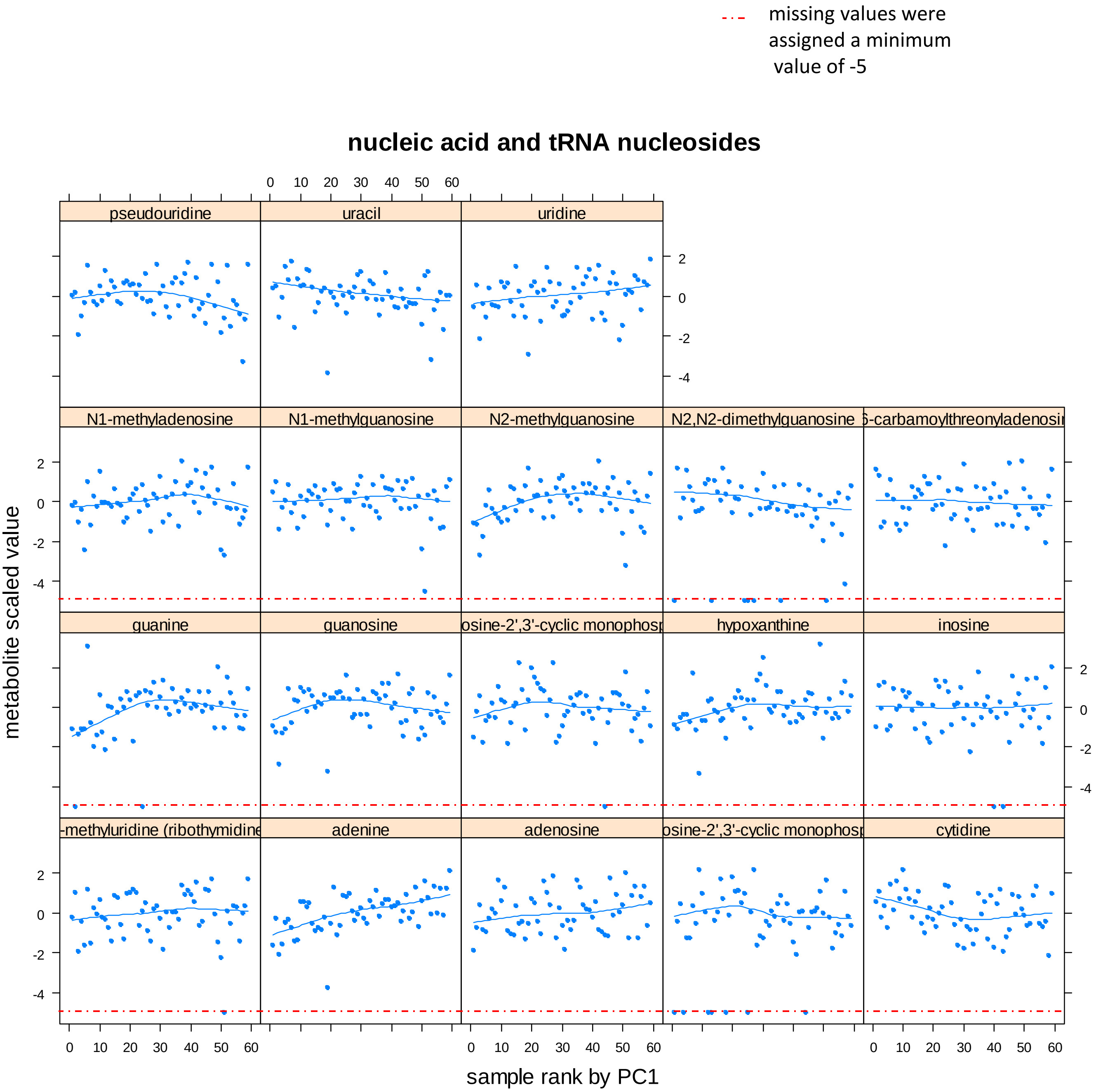

missing values were
assigned a minimum
value of -5
nucleic acid and tRNA nucleosides
pseudouridine
uracil
uridine
N1-methyladenosine
N1-methylguanosine
N2-methylguanosine
N2,N2-dimethylguanosine
guanine
guanosine
hypoxanthine
inosine
adenine
adenosine
cytidine
metabolite scaled value
sample rank by PC1

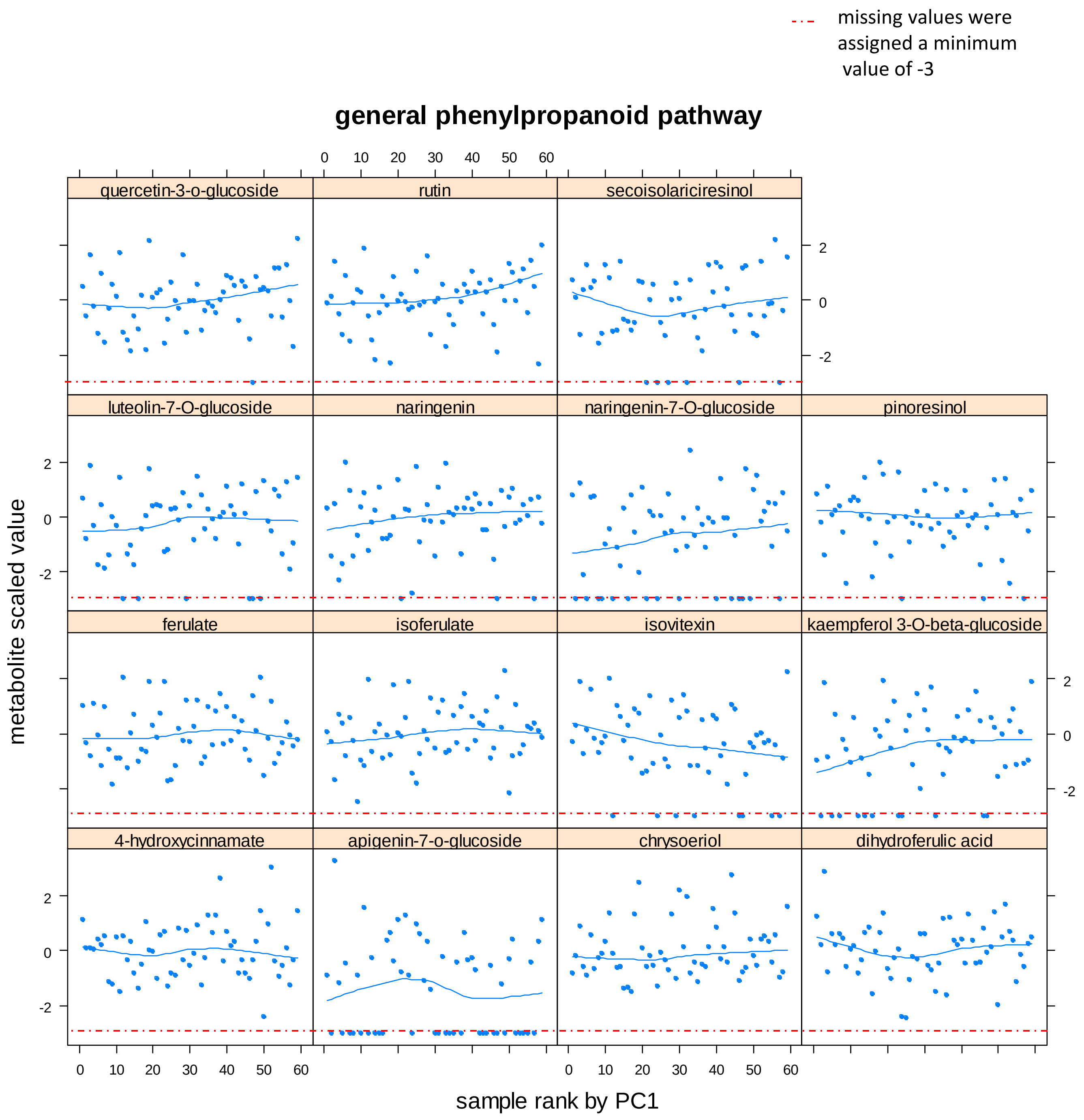

missing values were assigned a minimum value of -3
general phenylpropanoid pathway
quercetin-3-o-glucoside
rutin
secoisolariciresinol
luteolin-7-O-glucoside
naringenin
naringenin-7-O-glucoside
pinoresinol
ferulate
isoferulate
isovitexin
kaempferol 3-O-beta-glucoside
4-hydroxycinnamate
apigenin-7-o-glucoside
chrysoeriol
dihydroferulic acid
metabolite scaled value
sample rank by PC1
0
10
20
30
40
50
60
2
0
-2

vitamins

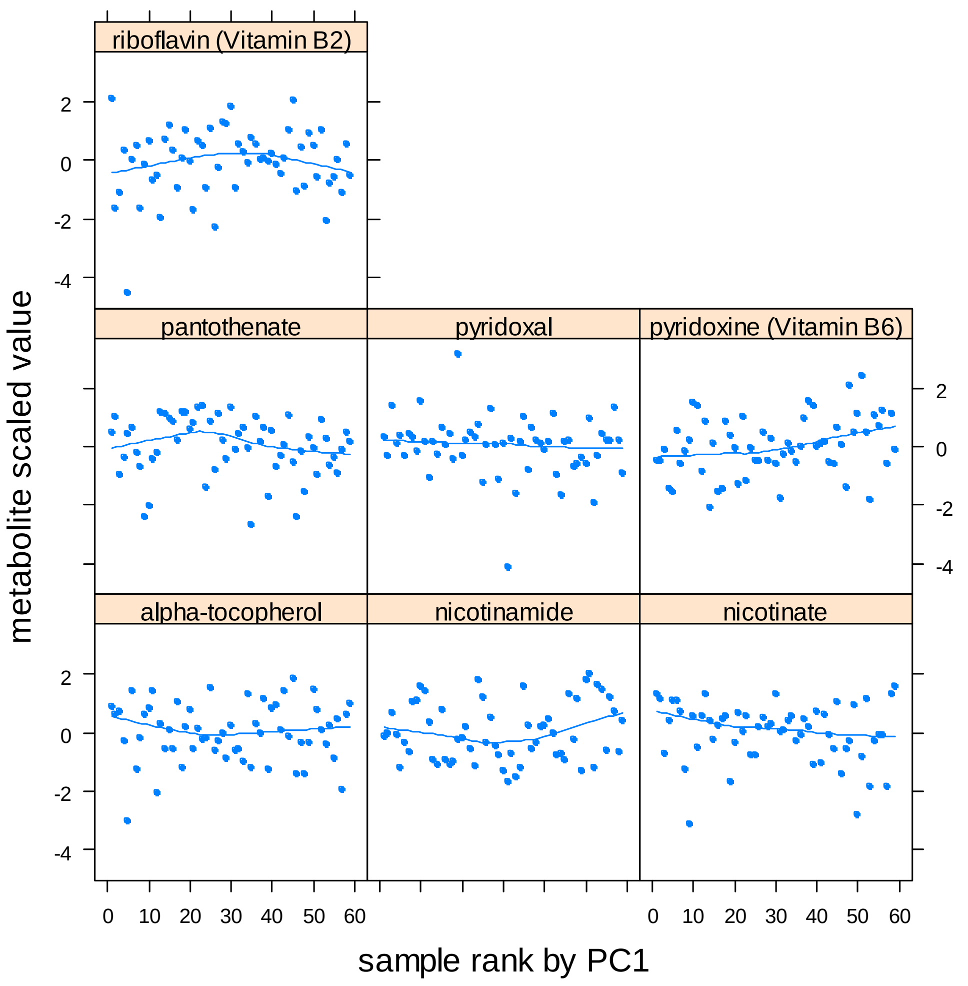

sphingoid bases

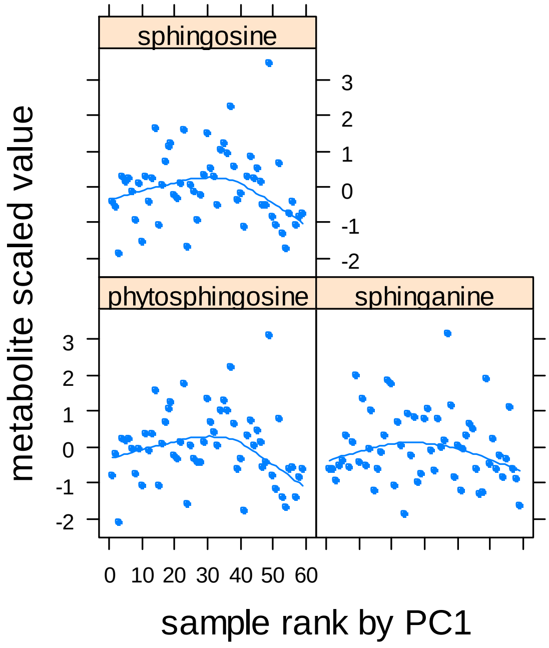

TCA

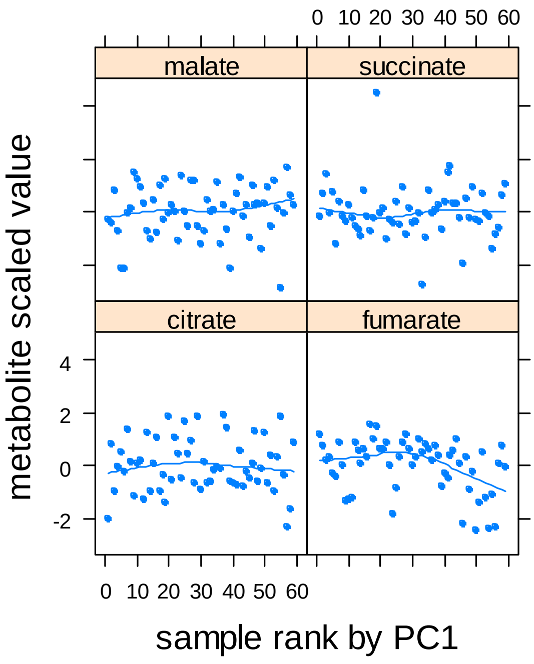

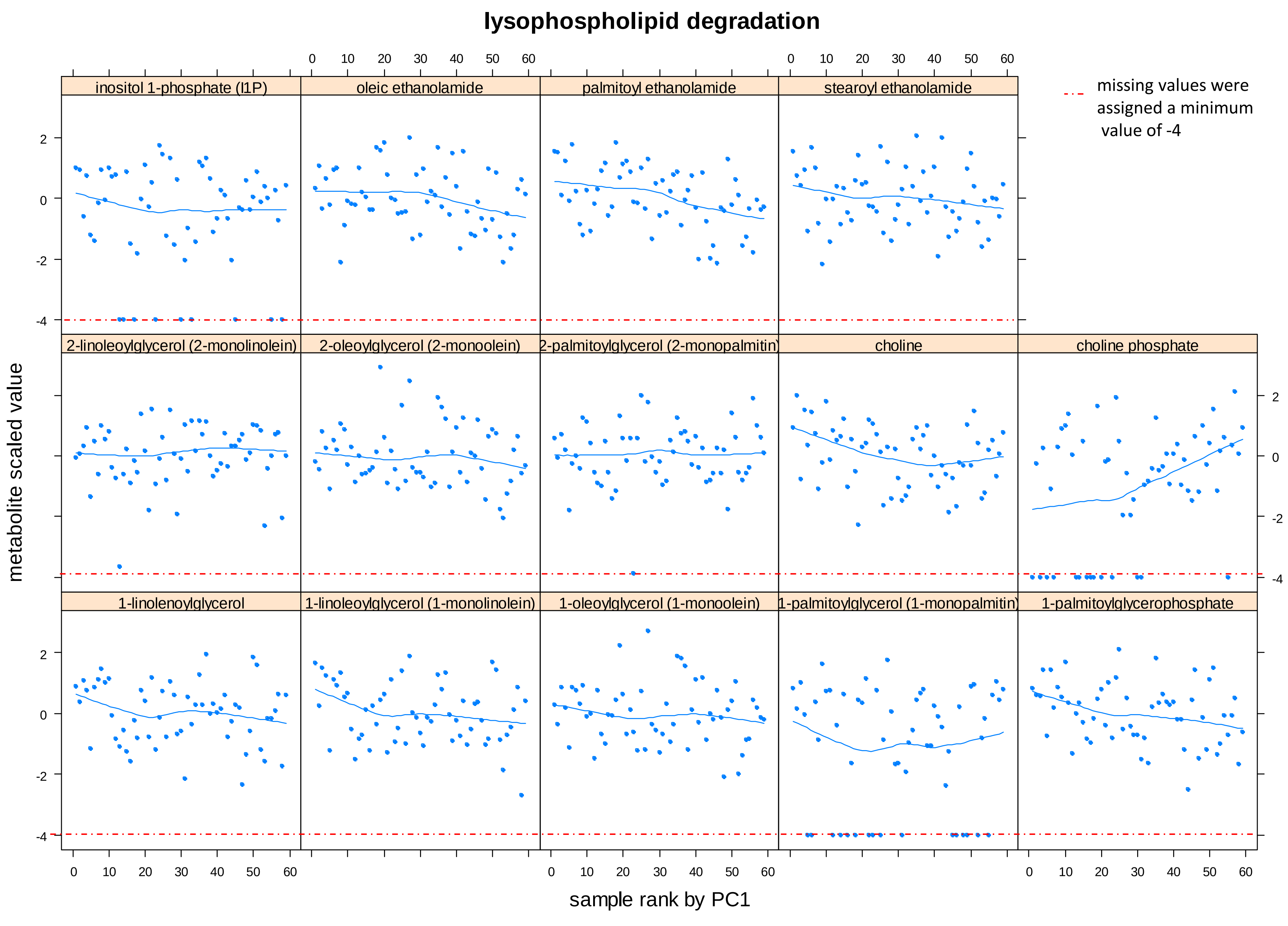

lysophospholipid degradation
inositol 1-phosphate (I1P)
oleic ethanolamide
palmitoyl ethanolamide
stearoyl ethanolamide
2-linoleoylglycerol (2-monolinolein)
2-oleoylglycerol (2-monoolein)
2-palmitoylglycerol (2-monopalmitin)
choline
choline phosphate
1-linolenoylglycerol
1-linoleoylglycerol (1-monolinolein)
1-oleoylglycerol (1-monoolein)
1-palmitoylglycerol (1-monopalmitin)
1-palmitoylglycerophosphate
missing values were assigned a minimum value of -4
metabolite scaled value
sample rank by PC1

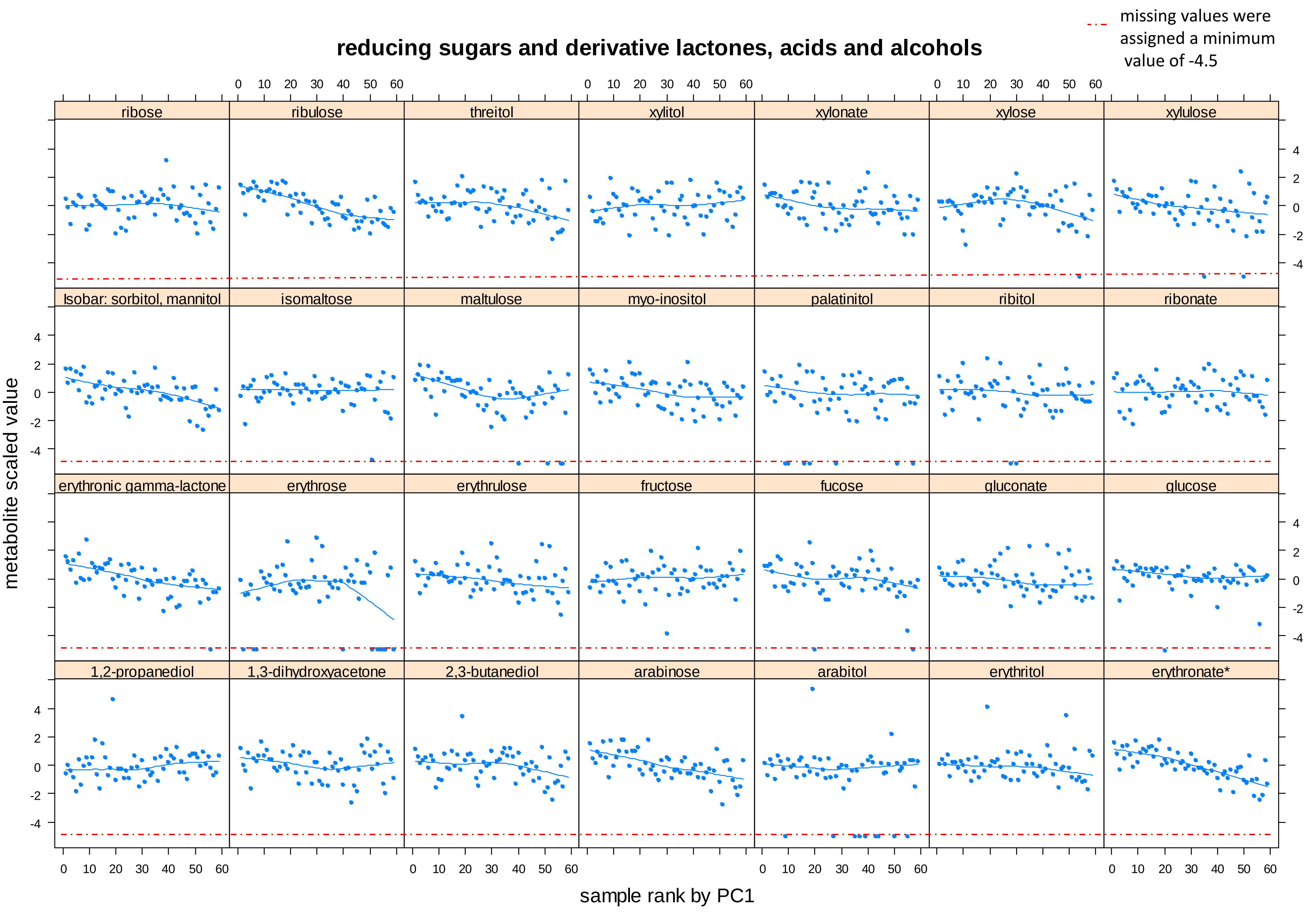

reducing sugars and derivative lactones, acids and alcohols
missing values were assigned a minimum value of -4.5
ribose
ribulose
threitol
xylitol
xylonate
xylose
xylulose
Isobar: sorbitol, mannitol
isomaltose
maltulose
myo-inositol
palatinitol
ribitol
ribonate
erythronic gamma-lactone
erythrose
erythrulose
fructose
fucose
gluconate
glucose
1,2-propanediol
1,3-dihydroxyacetone
2,3-butanediol
arabinose
arabitol
erythritol
erythronate*
metabolite scaled value
sample rank by PC1

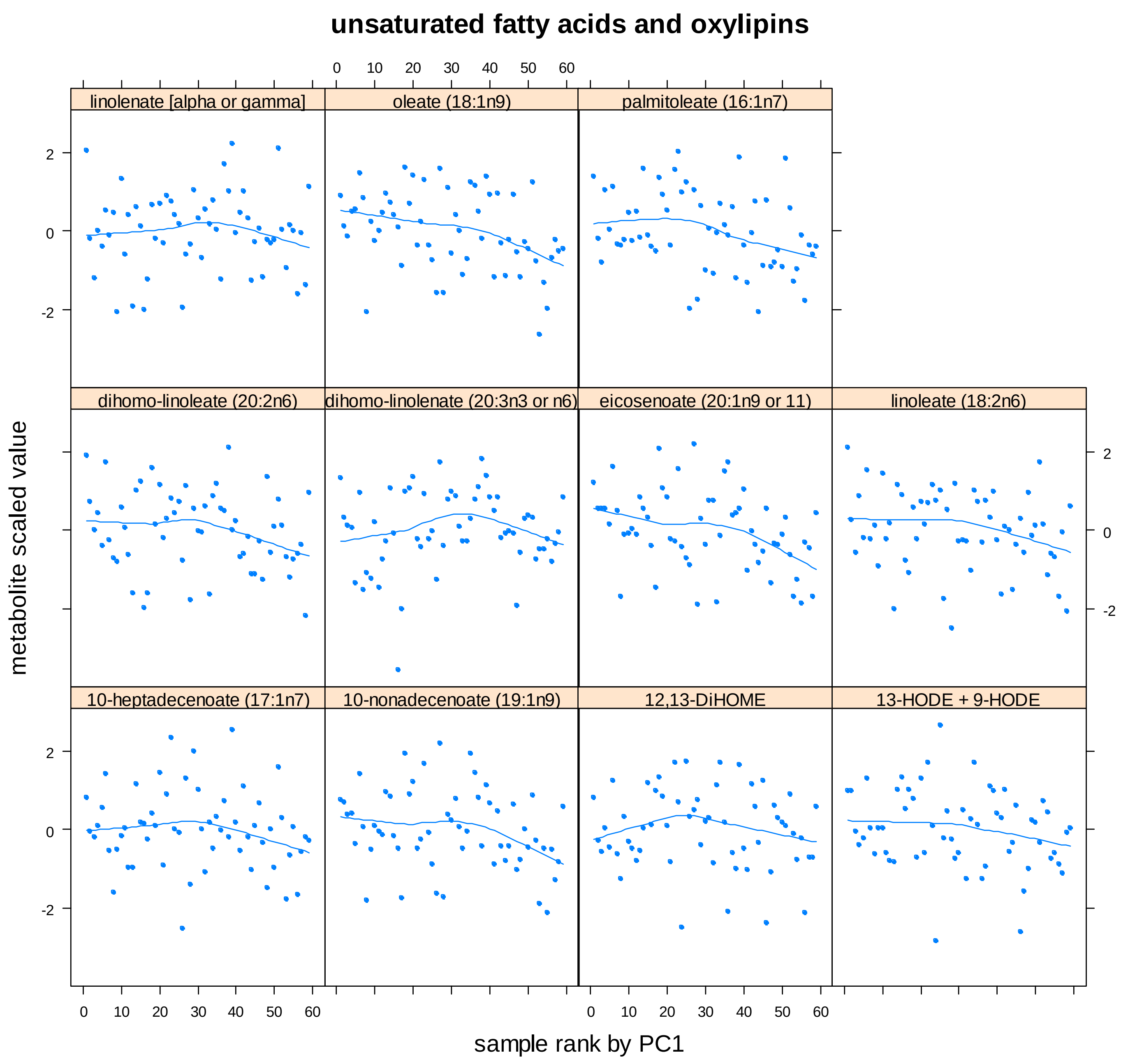
unsaturated fatty acids and oxylipins
linolenate [alpha or gamma]
oleate (18:1n9)
palmitoleate (16:1n7)
dihomo-linoleate (20:2n6)
dihomo-linolenate (20:3n3 or n6)
eicosenoate (20:1n9 or 11)
linoleate (18:2n6)
10-heptadecenoate (17:1n7)
10-nonadecenoate (19:1n9)
12,13-DiHOME
13-HODE + 9-HODE
metabolite scaled value
sample rank by PC1
0
10
20
30
40
50
60
2
0
-2

- · - missing values were assigned a minimum value of -4

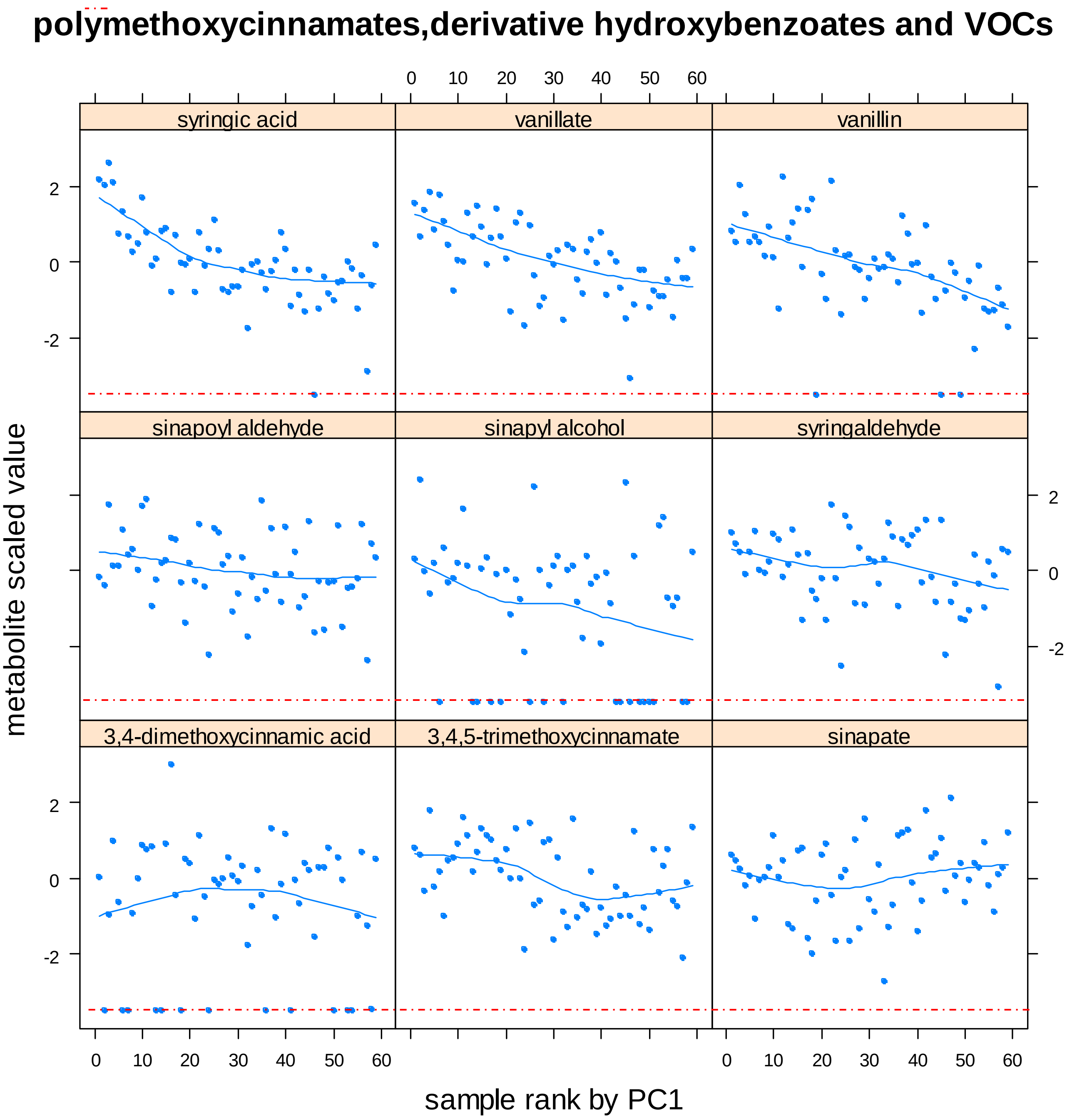

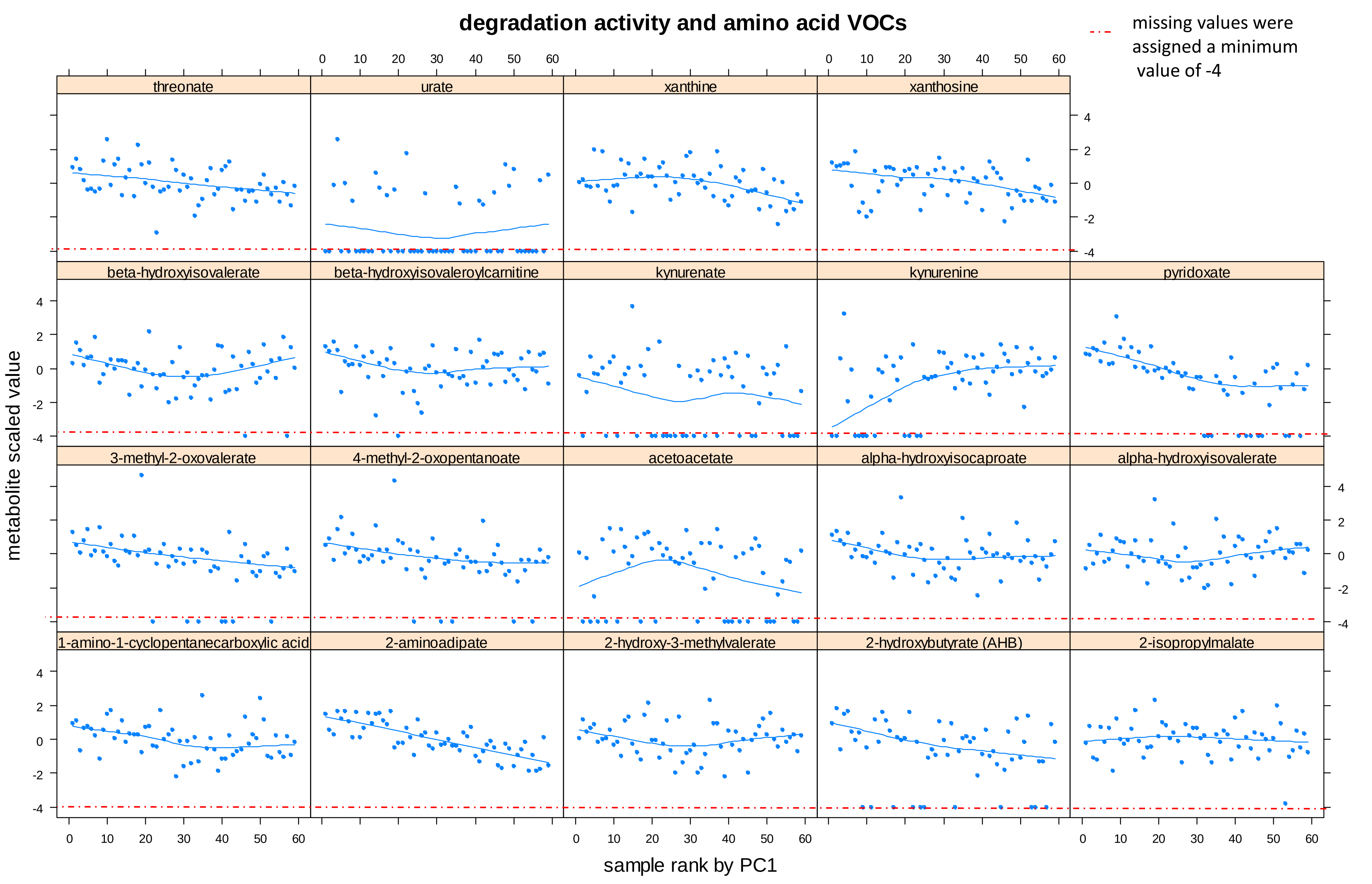
degradation activity and amino acid VOCs
missing values were assigned a minimum value of -4
metabolite scaled value
sample rank by PC1
threonate
urate
xanthine
xanthosine
beta-hydroxyisovalerate
beta-hydroxyisovaleroylcarnitine
kynurenate
kynurenine
pyridoxate
3-methyl-2-oxovalerate
4-methyl-2-oxopentanoate
acetoacetate
alpha-hydroxyisocaproate
alpha-hydroxyisovalerate
1-amino-1-cyclopentanecarboxylic acid
2-aminoadipate
2-hydroxy-3-methylvalerate
2-hydroxybutyrate (AHB)
2-isopropylmalate

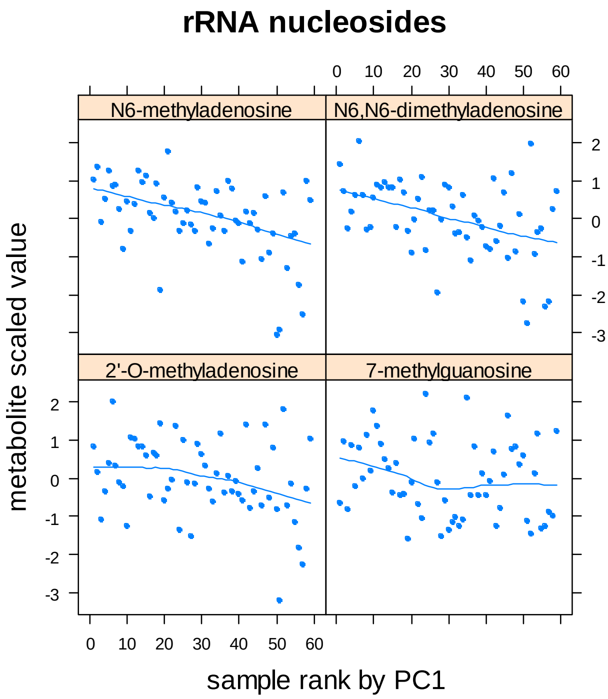
rRNA nucleosides
N6-methyladenosine
N6,N6-dimethyladenosine
2'-O-methyladenosine
7-methylguanosine
metabolite scaled value
sample rank by PC1

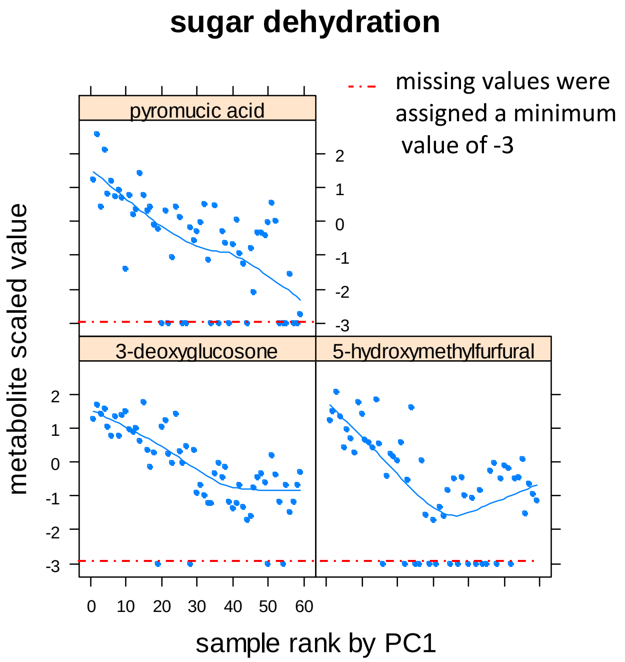
sugar dehydration
missing values were assigned a minimum value of -3
pyromucic acid
3-deoxyglucosone
5-hydroxymethylfurfural
metabolite scaled value
sample rank by PC1

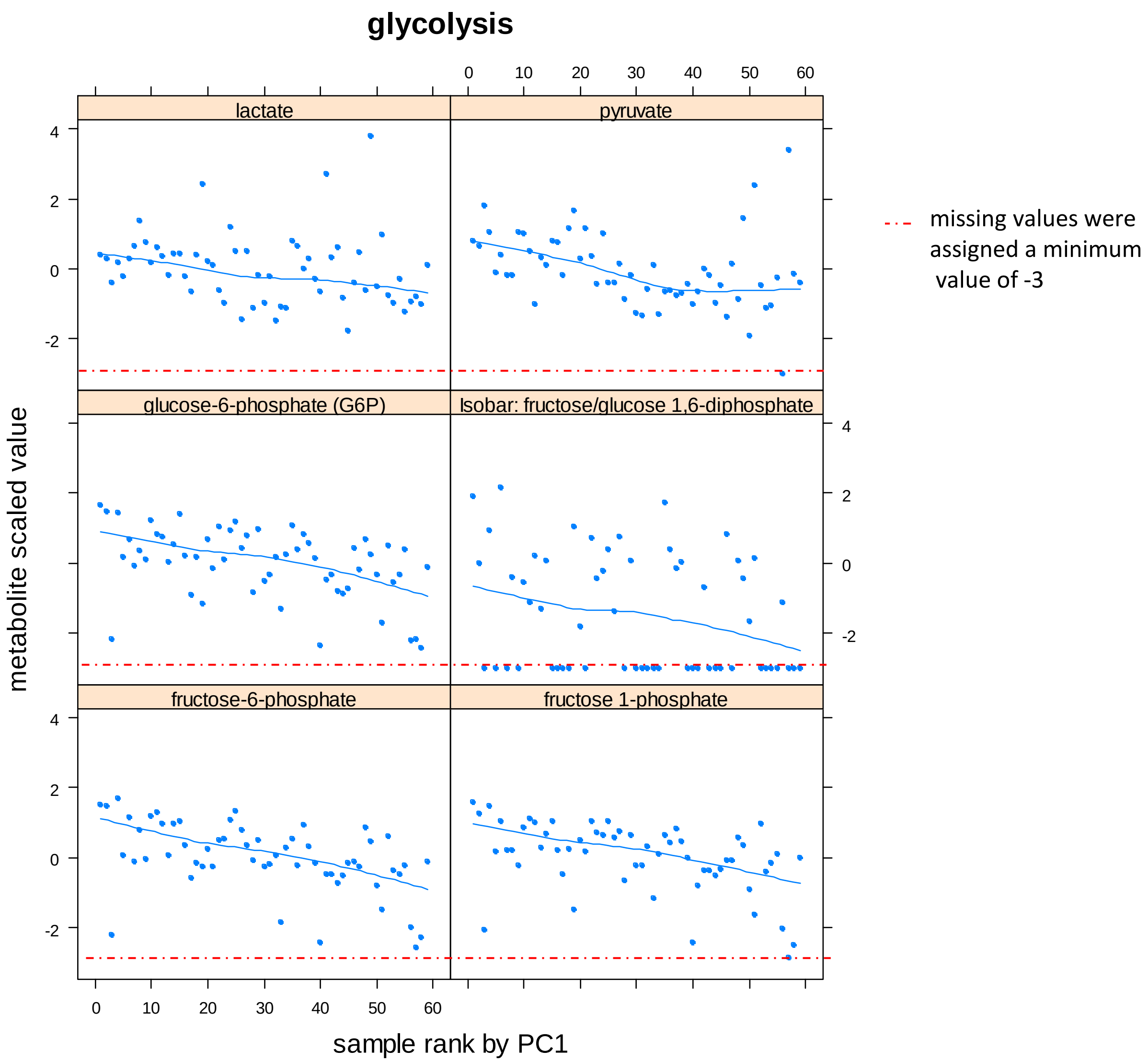
glycolysis
missing values were assigned a minimum value of -3
lactate
pyruvate
glucose-6-phosphate (G6P)
Isobar: fructose/glucose 1,6-diphosphate
fructose-6-phosphate
fructose 1-phosphate
metabolite scaled value
sample rank by PC1
